# Synthesis of Selenium-Decorated *N*-Oxide Isoquinolines: Arylseleninic Acids in Selenocyclization Reactions

**DOI:** 10.1021/acs.joc.4c00944

**Published:** 2024-08-01

**Authors:** João M. Anghinoni, Sabrina S. Ferreira, Jean C. Kazmierczak, Gelson Perin, Filipe Penteado, Eder J. Lenardão

**Affiliations:** † Centro de Ciências Químicas, Farmacêuticas e de Alimentos (CCQFA), Universidade Federal de Pelotas (UFPel), P.O. Box 354, Pelotas, 96010-900 Rio Grande do Sul, Brazil; ‡ Centro de Ciências Exatas e Naturais, Departamento de Química, Universidade Federal de Santa Maria (UFSM), Av. Roraima, Building 18, Santa Maria, 97105-340 Rio Grande do Sul, Brazil

## Abstract

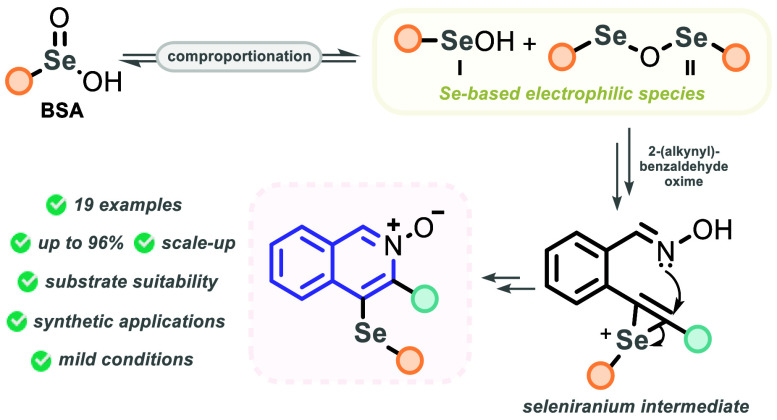

Herein, we describe the use of benzeneseleninic acid derivatives (BSA) as a bench-stable and easy to handle selenium reagent to access 4-(selanyl)isoquinoline-*N*-oxides through the selenocyclization of *o*-alkynyl benzaldehyde oximes. The reaction is conducted in refluxing methanol, allowing the thermal generation of electrophilic selenium species in situ. By this new protocol, a library of 19 selenium-decorated *N*-oxide isoquinolines was accessed in up to 96% yield with an outstanding substrate tolerance and the feasibility to scale it up 10 times (from 0.25 to 2.5 mmol).

## Introduction

**Figure 1:**
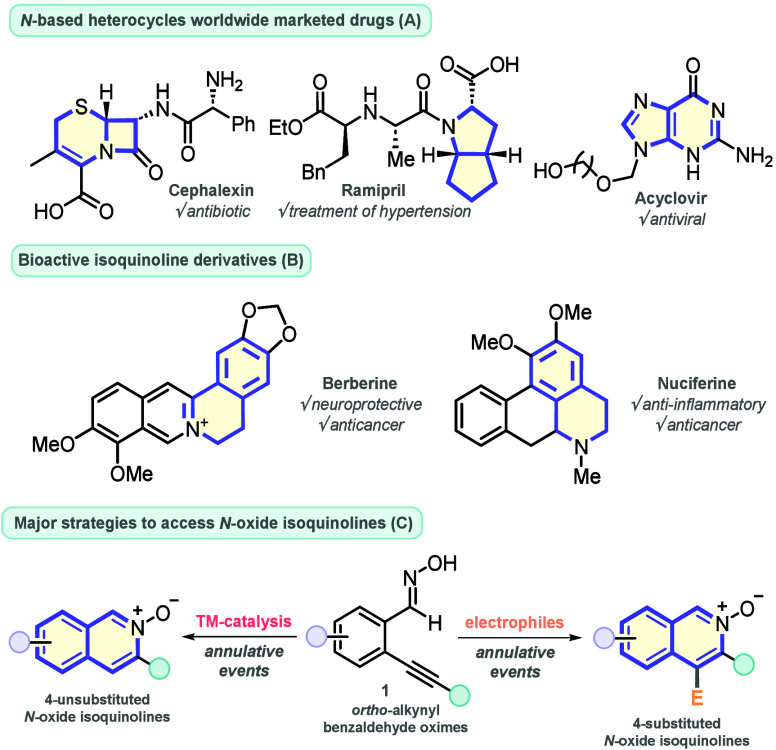
Bioactive *N*-based heterocycles, and synthetic strategies to prepare *N*-oxide isoquinolines.

*N*-Based heterocycles are a remarkable class of compounds widely found in nature, which play a pivotal role in the pharmaceutical industry due to their outstanding bioactivity, high stability, and operational efficiency in the human body.^1^ The importance of these compounds can be summarized by considering that they are broadly found on the main structural core of important worldwide-marketed drugs,^2^ including (1) Cephalexin,^3^ an antibiotic used for the treatment of several bacterial infections, (2) Ramipril,^4^ used for the treatment of hypertension, and (3) Acyclovir,^5^ an antiviral medicine used for the treatment of some sexually transmitted virus, including herpes virus (Figure 1A). Isoquinoline-based alkaloids are particularly interesting compounds, which are present in several plants used in traditional Chinese medicine. For instance, Berberine demonstrated neuroprotective^6^ and anticancer activities,^7^ while Nuciferine has neuroprotective^6^ and anti-inflammatory ones^8^ (Figure 1B). In this context, *N*-oxide heterocycles (derived from pyridine, quinoline, and isoquinoline cores) are a privileged class of compounds with a large scope of applications in materials science.^9^ Additionally, they present relevant pharmacological activities, including antimicrobial, antiviral (HIV), antifungal, and anticancer ones.^10^ Therefore, interest in the development of efficient synthetic protocols to access this class of compounds has been growing steadily in recent years, and several approaches have appeared in the literature.^11^ Considering isoquinoline *N*-oxide derivatives, the major strategies to prepare them are based on the electrophilic cyclization of *o*-alkynyl benzaldehyde oxime derivatives in the presence of transition metal (TM) catalysts or electrophilic species, which in general are generated in situ by oxidative events (Figure 1C).^12^

Another outstanding class of bioactive molecules is the organoselenium compounds, which have demonstrated important pharmacological activities. A notable example is Ebselen, which is widely known as a GPx-mimic antioxidant agent,^13^ which very recently has demonstrated antiviral activity against SARS-CoV-2 virus.^14^ A significant part of the synthetic bioactive organoselenium derivatives are obtained when hybridized with *N*-based heterocycles,^15^ as, for example, in the case of some selenium-decorated quinoline derivatives, which have demonstrated superior anti-inflammatory activity in comparison to Meloxicam.^16^ In this context, the main effective synthetic strategies to install organoselenium groups are through the use of Se-based electrophilic reagents (e.g., PhSeCl and PhSeBr) and by the generation in situ of related species in the presence of TM-based catalyst (e.g., Cu, Fe, and Ag)^17^ or oxidant species (e.g., I_2_, Oxone, and persulfates).^18^

**Scheme 1:**
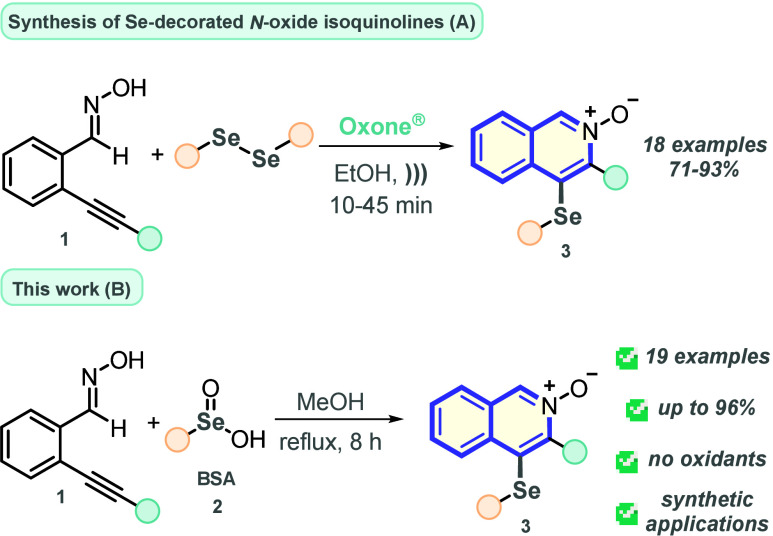
Synthetic Approach for the Synthesis of Se-Decorated *N*-Oxide Isoquinolines

Within this framework, some of us^19^ have reported for the first time the synthesis of 4-(selanyl)isoquinoline-*N*-oxides **3** by reacting *o*-alkynyl benzaldehyde oximes **1** and diorganyl diselenides in the presence of Oxone under ultrasound irradiation. Although exhibiting short reaction times and an outstanding yield range, important drawbacks are still faced in this reaction, like the requirement for using high oxidant loading, increasing the generation of waste at the end of the process (Scheme 1A).

On the other hand, benzeneseleninic acid (BSA, **2**) derivatives are inodorous and easy to handle reagents, besides being bench stable, not requiring special storage conditions. Recently, we have devoted our energy to demonstrate the synthetic applicability of BSA as a Se-based reagent to deliver Se(II)-decorated structures, under thermal and photocatalytic conditions, not requiring the use of strong oxidant species, reaction auxiliaries, or additives.^20^ Another important feature of BSA is that after the reaction water is the only produced waste.

Thus, as a continuation of our efforts to demonstrate the fabulous applicability of BSA as an electrophilic selenium precursor reagent, we depict herein an efficient protocol to prepare 4-(selanyl)isoquinoline-*N*-oxides **3** by reacting *o*-alkynyl benzaldehyde oxime **1** and BSA **2** derivatives under thermal conditions, circumventing the use of strong oxidant conditions (Scheme 1B).

## Results and Discussion

Aiming to establish the optimal reaction conditions, 2-(phenylethynyl)benzaldehyde oxime **1a** and benzeneseleninic acid **2a** were elected as the standard substrates to prepare 4-(phenylselanyl)isoquinoline-*N*-oxide **3a** (Table 1).

**Table 1:**
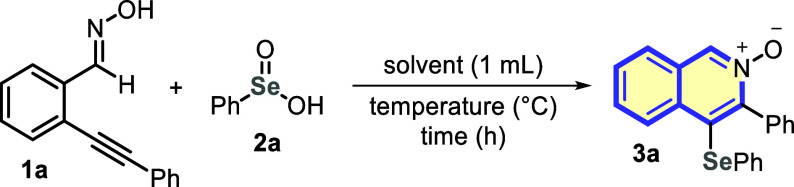
Reaction Optimization Study for the Synthesis of **3a**^a^

no.	solvent	temperature (°C)	time (h)	yield (%)
1	DMF	80	16	27
2	DMF	120	16	6
3	^*t*^BuOH	80	16	55
4	^*i*^PrOH	80	16	75
5	EtOH	78	16	92
6	MeOH	65	16	96
7	glycerol	80	16	ND
8	DCE	84	16	69
9	MeCN	80	16	67
10	MeOH	65	8	96
11	MeOH	65	4	54
12^b^	MeOH	65	8	74
13	MeOH	25	8	ND

a In a round bottomed flask, 2-alkynylbenzaldoxime **1a** (0.25 mmol), benzeneseleninic acid **2a** (0.5 mmol), and the solvent (1.0 mL) were added. The resulting mixture was heated using an oil bath (tabled temperatures), and the system was stirred by the indicated time. After workup, the reaction crude was purified by column chromatography.

b A 0.25 mmol amount of **2a** was used. ND = not detected.

Based on our recent results on the thermal selenylation of heteroarenes,^20a,20d^ we started our studies by stirring a mixture of substrates **1a** (0.25 mmol) and **2a** (0.5 mmol) in DMF at 80 °C for 16 h. Under these conditions, the desired isoquinoline *N*-oxide derivative **3a** was obtained in only 27% yield (Table 1, entry 1). Then, the reaction temperature was increased to 120 °C; however, a huge decrease in reaction efficiency was observed, and the product **3a** was isolated in 6% yield (Table 1, entry 2). Following, an extensive solvent screening study was performed at different temperatures, employing several protic and aprotic polar solvents, like ^*t*^BuOH, ^*i*^PrOH, EtOH, MeOH, glycerol, DCE, and MeCN (Table 1, entries 3–9).

In general, a very expressive improvement was achieved with MeOH presenting outstanding performance when used at reflux temperature, affording the product **3a** in 96% yield (Table 1, entry 6). Following, the reaction time was reduced from 16 h to 8 and 4 h, allowing the obtention of **3a** in 96% and 54% yields, respectively. Thus, 8 h was set as the best reaction time (Table 1, entries 10 and 11). Therefore, the reaction stoichiometry was tuned to 1:1, and the yield of **3a** decreased to 79% yield (Table 1, entry 12). Finally, by conducting the process at room temperature, the formation of product **3a** was completely suppressed and the starting materials were recovered (Table 1, entry 13).

With the best reaction conditions in hand (Table 1, entry 10), a broad reaction scope study was performed, employing several substrates **1a**–**f** and **2a**–**g** to construct a wide library of organoselenium-decorated *N*-oxide isoquinoline derivatives **3a**–**r** (Table 2). Initially, benzeneseleninic acid **2a** reacted with several 2-(phenylethynyl)benzaldehyde oximes **1b**–**d**, bearing electron-rich and chloro-substituted aromatic rings attached to the C≡C bond. All substrates have demonstrated outstanding suitability to the protocol, affording the corresponding products **3b**, **3c**, and **3d** in 83%, 89%, and 85% yields, respectively. Following, the ability of other BSA derivatives **2** to be employed as substrate in the reaction was evaluated. Thus, *p*-methyl- and *p*-fluoro-substituted benzeneseleninic acids **2b** and **2c** were reacted with **1a** to be converted to the respective *N*-oxide isoquinoline derivatives **3e** and **3f**, which were correspondingly isolated in 87% and 90% yields. Notably, the electron-deficient *m*-trifluoromethylbenzeneseleninic acid **2d** reacted smoothly with **1a** to afford the respective product **3g** in 94% yield. Additionally, bulky BSA derivatives **2e** and **2f** (naphthyl and mesityl derivatives) were satisfactorily used to be converted to products **3h** and **3i** in 80% and 81% yields, respectively. These results highlight an important protocol feature, that is, the feasibility of application of sterically hindered substrates, without remarkable loss of efficiency. The chloro-containing *o*-alkynyl benzaldehyde oxime **1d** satisfactorily reacted with *p*-methyl- and *p*-fluoro-substituted benzeneseleninic acids **2b** and **2c** to deliver the corresponding compounds **3j** and **3k** in 72% and 80% yields. These results also emphasize the protocol suitability for reactions among *o*-alkynyl benzaldehyde oxime **1a**–**d** and BSA **2a**–**g** derivatives, overcoming such electronic and steric effects (Table 2).

**Table 2:**
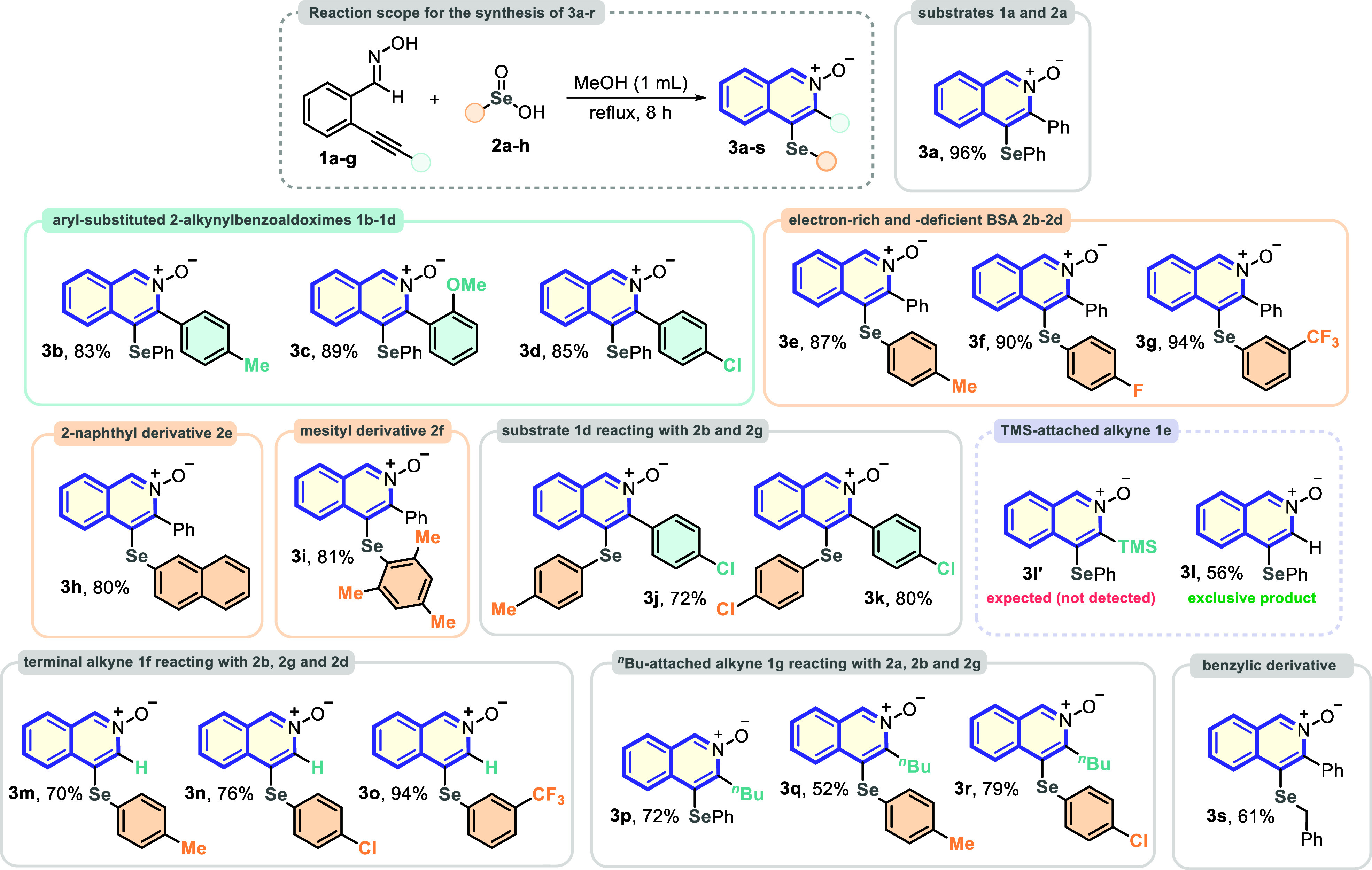
Reaction Scope Study for the Synthesis of the Products **3a**–**s**^a^

a In a round-bottomed flask, a mixture of substrate **1** (0.25 mmol) and **2** (0.5 mmol) in MeOH (1.0 mL) was stirred for 8 h under reflux using an oil bath. All products (**3a**–**s**) were purified by column chromatography.

In the sequence, the C≡C–TMS-derived *o*-alkynyl benzaldehyde oxime **1e** was employed as substrate in the reaction with BSA **2a**. Surprisingly, at the end of the process, the expected TMS-containing product **3l′** was not observed, and the C3-unsubstituted 4-(phenylselanyl)isoquinoline 2-oxide **3l** was exclusively obtained in 56% yield. Therefore, the terminal *o*-alkynyl benzaldehyde oxime **1f** was reacted with electron-rich and -deficient BSA derivatives **2b**, **2g**, and **2d** to be efficiently converted to the C3-unsubstituted products **3m**, **3n**, and **3o** in 70%, 76%, and 94% yields, respectively. These results open synthetic possibilities, allowing the application of these products in further transformations in the C3 reaction site (Table 2).

Finally, the effect of the presence of an alkyl substituent at the C≡C bond was evaluated by using the ^*n*^Bu-attached *o*-alkynyl benzaldehyde oxime **1g** in reactions with BSA derivatives **2a**, **2b**, and **2g** under the optimal conditions. Satisfactorily, C3-alkyl products **3p**, **3q**, and **3r** were afforded in 72%, 52%, and 79% yields, also demonstrating the versatility of the protocol in efficiently delivering 3-alkyl-substituted products (Table 2). Additionally, benzylic seleninic acid **2h** reacted smoothly with *o*-alkynyl benzaldehyde oxime **1a** to afford the corresponding product **3s** in 61% yield. This result indicates that the C(sp^3^)–Se bond remains intact in the reaction.

**Scheme 2:**
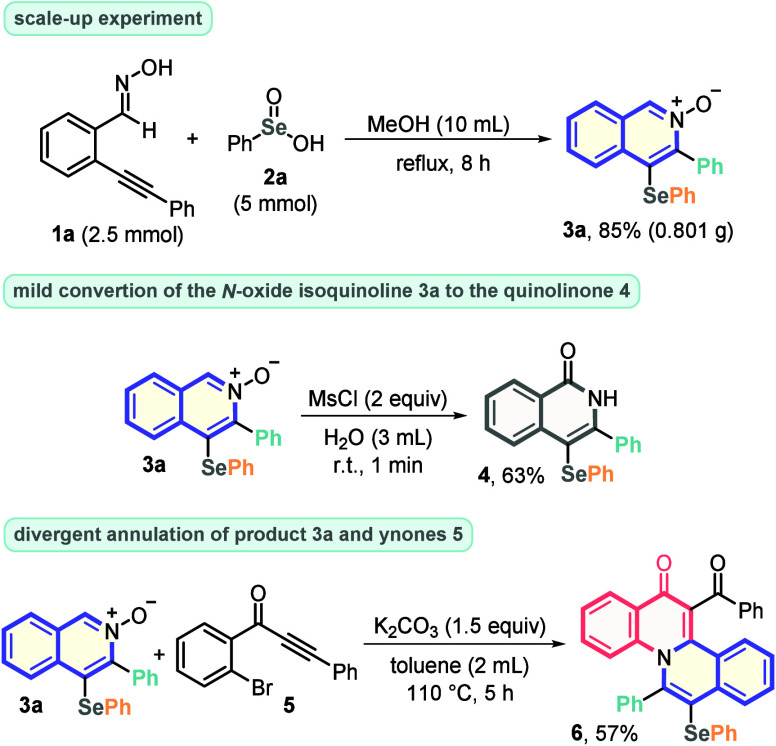
Scale-up Experiments and Synthetic Applications of **3a**

Aiming to investigate the protocol usefulness, a scale-up experiment was performed, increasing the reaction scale by 10 times (from 0.25 to 2.5 mmol). Accordingly, the reaction of **1a** with **2a** was satisfactorily conducted, remarkably affording the expected product **3a** in 85% yield (0.801 g). Furthermore, the Se-decorated *N*-oxide isoquinoline **3a** was employed as substrate in some synthetic transformations. First, **3a** was reacted with MsCl in water to give 3-phenyl-4-(phenylselanyl)isoquinolin-1(2*H*)-one **4** in 63% yield.^21^ Moreover, **3a** also reacted with *o*-bromo ynone **5** in the presence of K_2_CO_3_, under thermal conditions, to be smoothly converted to the polycyclic product **6** by the simultaneous C–N and C–C bond construction (Scheme 2).^22^

**Scheme 3:**
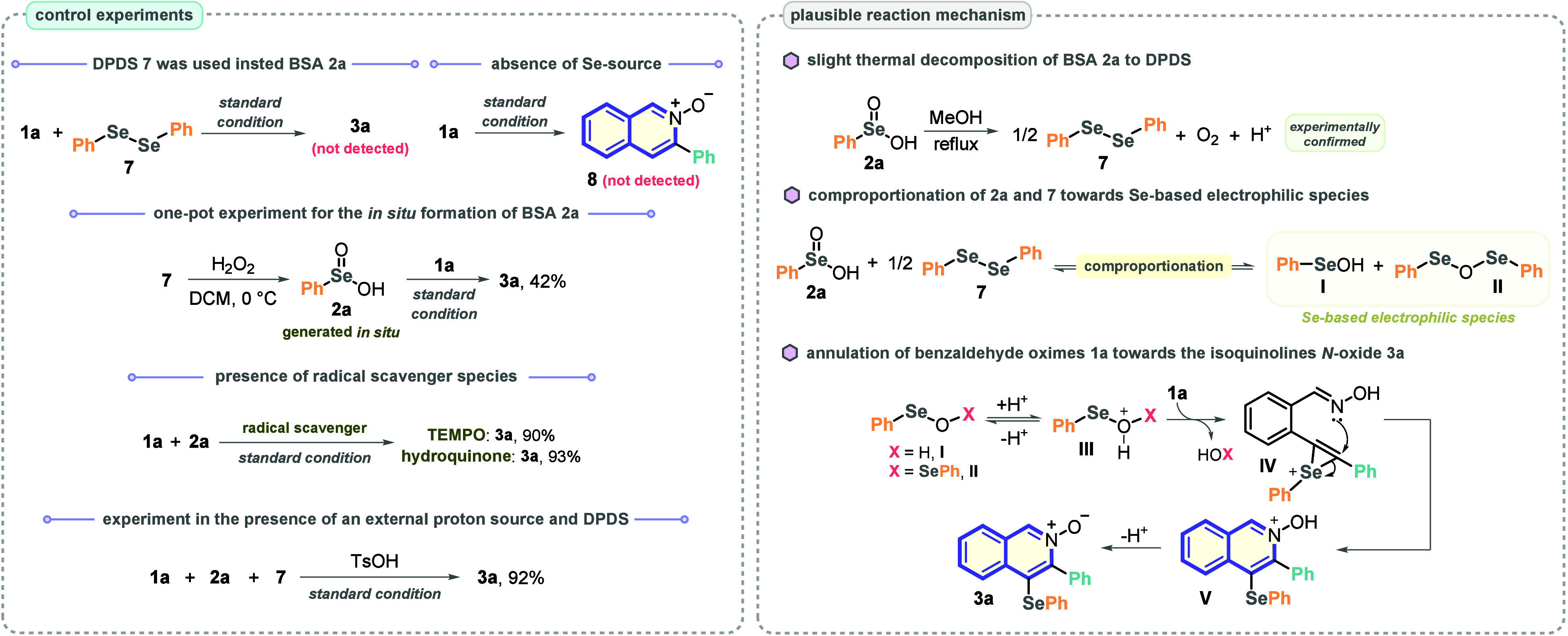
Control Experiments and Plausible Mechanism

In view of the excellent outcomes on the use of BSA **2** as a selenylating agent in the selenocyclization of *o*-alkynyl benzaldehyde oximes **1**, several control experiments were conducted to gain influential mechanistic insights. Initially, the standard reaction condition (Table 1, entry 10) was performed in the presence of diphenyl diselenide (DPDS, **7**) as substrate instead of BSA **2a**, and the formation of product **3a** was not observed, demonstrating that even if PhSeSePh is formed by thermal decomposition of **2a**, it is not reactive enough to trigger the transformation. We also observed that in the absence of BSA, the corresponding product from the intramolecular cyclization of **1a** (compound **8**) was not formed, pointing out that the main reaction pathway does not follow a previous intramolecular annulation of **1a** (Scheme 3).

Following, a one-pot experiment was run by generating BSA **2a** in situ (PhSeSePh + H_2_O_2_) and subsequent addition of **1a** (1 equiv). Under this condition, the desired product **3a** was achieved poorly in 42% yield (vs 74% yield starting from BSA; see Table 1, entry 12). Even though this result shows that a one-pot strategy is not advantageous, in view of the facility to afford BSA **2a** purely, it also highlights the mandatory presence of BSA **2a** as reagent for the success of the transformation. Aiming to verify if the main reaction pathway involves radical events, two experiments were carried out in the presence of 3 equiv of radical scavengers (TEMPO and hydroquinone). In both, no significant change in the reaction efficiency was observed, suggesting that the main reaction events follow ionic pathways (Scheme 3).

Based on these results and on our previous advances in the chemistry of BSA,^20^ a plausible reaction mechanism was proposed. Initially, the process is fully dependent on a thermal decomposition of BSA **2a** toward PhSeSePh **7**. This hypothesis was experimentally confirmed by submitting just **2a** under the optimized reaction conditions. The development of a yellowish color was observed, and subsequently, the presence of a small amount of **7** was confirmed by ^77^Se NMR. Besides, it is supported by the fact that the transformation is completely suppressed when the reaction is conducted at room temperature (Table 1, entry 13), pointing out a pivotal thermal decomposition of **2a**. Following, under heating, **2a** and **7** can undergo a comproportionation process to the selenium electrophilic species **I** and **II**.^23^ Finally, these species are eventually protonated to reach the intermediates **III**, which are more electrophilic to react with the C≡C bond present in **1a**, delivering the seleniranium intermediate **IV**. To verify the reasonableness of these events (the comproportionation in the presence of DPDS **7** and the protonation of intermediates **I** and **II**), **1a** was reacted with a mixture of **2a** and DPDS **7** (2:1 ratio) in the presence of 1 equiv of *p*-toluenesulfonic acid (TsOH), yielding the product **3a** in 92% yield. Finally, the intramolecular annulation of **IV** drives the transformation toward the cyclized intermediate **V**, which is finally deprotonated to be converted to the desired product **3a** (Scheme 3, plausible reaction mechanism).

## Conclusions

An alternative protocol to prepare Se-decorated isoquinoline *N*-oxide derivatives in moderate to excellent yields (52–96%) was developed by reacting *o*-alkynyl benzaldehyde oximes and benzeneseleninic acid derivatives under thermal conditions. The strategy allowed conducting reactions in the absence of oxidant and other auxiliary species, delivering mild, safe, and easy to handle procedures. Besides, the protocol presents outstanding substrate suitability, demonstrating tolerance to electron-donating and -withdrawing groups attached to aromatic systems as well as to alkyl groups linked to the C≡C bond. Taken together, these features point out a new avenue of applications of BSA derivatives as a source of Se(II) electrophilic species under thermal conditions, performing electrophilic cyclization processes under oxidant- and metal-free mild reaction conditions.

## Experimental Section

### General Information

The reactions were monitored by TLC carried out on Merck silica gel (60 F_254_). For visualization, TLC plates were either placed under UV light, stained with iodine vapor and 5% vanillin in 10% H_2_SO_4_ and heat. Column chromatography was performed using Merck Silica Gel (pore size 60 Å, 230–400 mesh). Low-resolution mass spectra (MS) were measured on a Shimadzu GC-MS-QP2010 mass spectrometer. The HRMS analyses were performed in a HESI Quadrupole-Orbitrap (Q extractive focus, Thermo Scientific) spectrometer equipped with an APCI source operating in positive mode. The samples were solubilized in methanol and analyzed by direct infusion at a constant flow rate. The acquisition parameters were: Scan type Full MS; resolution 70000; polarity positive. Ionization conditions HESI: Sheath gas 20; aux gas 10; spray voltage 2.8 kV; capillary temperature 300 °C. The mass-to-charge ratio (*m*/*z*) data were processed and analyzed using Bruker Daltonics softwares: Compass Data Analysis and Isotope Pattern. Hydrogen nuclear magnetic resonance (^1^H NMR), Carbon-13 nuclear magnetic resonance (^13^C{^1^H} NMR), and Selenium-77 nuclear magnetic resonance (^77^Se{^1^H} NMR) were obtained on Bruker Avance III HD spectrometer at 400, 100, 76, and 400 MHz, respectively. Spectra were recorded in CDCl_3_ solutions. Chemical shifts (δ) are reported in ppm, referenced to tetramethyl silane (TMS, δ 0.0 ppm) as the internal reference for ^1^H NMR and the solvent peak of CDCl_3_ (δ 77.23 ppm) for ^13^C{^1^H} NMR. Coupling constants (*J*) are reported in hertz. The ^77^Se{^1^H} NMR chemical shifts are reported in ppm relative to the external reference (C_6_H_5_Se)_2_ (δ 463 ppm). Abbreviations to denote the multiplicity of a particular signal are s (singlet), d (doublet), dd (doublet of doublets), ddd (doublet of doublet of doublets, td (triplet of doublets), tt (triplet of triplets), q (quartet), and m (multiplet). Melting point (mp) values were measured in a Marte PFD III instrument. The *o*-alkynyl benzaldehyde oximes **1a**–**g** were prepared by already described protocols.^24^ The and arylseleninic acids **2a**–**g** were previously prepared as described in the Supporting Information.^25^

### General Procedure for the Synthesis of 3-Organyl-4-(selanyl)isoquinoline-2-oxides (**3a**–**3s**)

To a 10.0 mL glass tube were added the appropriate oxime **1** (0.250 mmol, 1 equiv), arylseleninic acid **2** (0.5 mmol, 2 equiv), and MeOH (1.0 mL, 0.25 M). The resulting solution was stirred under reflux for 8 h. Posteriorly, the solvent was evaporated, and the crude product was purified via column chromatography using a 10:90 MeOH:EtOAc mixture as the eluent, affording the corresponding product **3**.

#### 3-Phenyl-4-(phenylselanyl)isoquinoline-2-oxide (**3a**)^19^

Purified by column chromatography (MeOH/EtOAc = 30:70). Yield: 90.2 mg (96%); amorphous reddish solid. Mp: 213-215 °C. Lit.:^19^ 213–215 °C. ^1^H NMR (CDCl_3_, 400 MHz) δ 8.95 (s, 1H), 8.36 (d, *J* = 8.1 Hz, 1H), 7.73 (d, *J* = 7.8 Hz, 1H), 7.62–7.54 (m, 2H), 7.43–7.39 (m, 3H), 7.30–7.27 (m, 2H), 7.14–7.07 (m, 3H), 7.04–7.00 (m, 2H). ^13^C{^1^H} NMR (101 MHz, CDCl_3_) δ 152.6, 137.9, 134.5, 131.9, 131.6, 130.7, 129.9, 129.8, 129.6, 129.5, 129.14, 129.11, 128.3, 128.1, 127.1, 125.5. ^77^Se{^1^H} NMR (76 MHz, CDCl_3_) δ 339.9.

#### 4-(Phenylselanyl)-3-(4-tolyl)isoquinoline-2-oxide (**3b**)^19^

Purified by column chromatography (MeOH/EtOAc = 30:70). Yield: 80.5 mg (83%); reddish solid. Mp: 148–150 °C. Lit.:^19^ 148–150 °C. ^1^H NMR (CDCl_3_, 400 MHz) δ 8.96 (s, 1H); 8.32 (d, *J* = 7.6 Hz, 1H), 7.74–7.69 (m, 1H), 7.59–7.51 (m, 2H), 7.25–7.18 (m, 5H), 7.11–7.09 (m, 2H), 7.05–7.01 (m, 2H) 2.40 (s, 3H). ^13^C{^1^H} NMR (101 MHz, CDCl_3_) δ 152.4, 139.1, 138.3, 131.9, 131.8, 131.4, 130.5, 130.2, 129.7, 129.5, 129.4, 129.0, 128.9, 128.1, 127.0, 125.8, 21.6. ^77^Se{^1^H} NMR (76 MHz, CDCl_3_) δ 330.8.

#### 3-(2-Methoxyphenyl)-4-(phenylselanyl)isoquinoline-2-oxide (**3c**)

Purified by column chromatography (MeOH/EtOAc = 30:70). Yield: 89.8 mg (89%); amorphous reddish solid. Mp: 148–150 °C. ^1^H NMR (CDCl_3_, 400 MHz) δ 9.01 (s, 1H), 8.27 (d, *J* = 8.4 Hz, 1H), 7.75 (d, *J* = 8.1 Hz, 1H), 7.59 (t, *J* = 7.2 Hz, 1H), 7.52 (t, *J* = 8.0 Hz, 1H), 7.45–7.34 (m, 3H), 7.31 (dd, *J* = 6.5, 3.1 Hz, 2H), 7.10 (td, *J* = 8.2, 1.4 Hz, 1H), 6.75 (d, *J* = 7.9 Hz, 1H), 6.64 (t, *J* = 7.8 Hz, 1H), 6.52 (dd, *J* = 7.7, 1.4 Hz, 1H), 3.74 (s, 3H). ^13^C{^1^H} NMR (101 MHz, CDCl_3_) δ 156.2, 152.8, 138.0, 134.4, 131.8, 129.9, 129.6, 129.5, 129.2, 129.0, 128.9, 128.2, 127.6, 126.7, 125.4, 121.3, 110.5, 55.8. ^77^Se{^1^H} NMR (76 MHz, CDCl_3_) δ 292.0. HRMS *m*/*z*: [M + H]^+^ calcd for C_22_H_18_NO_2_Se, 408.0498; found, 408.0495.

#### 3-(4-Chlorophenyl)-4-(phenylselanyl)isoquinoline-2-oxide (**3d**)^19^

Purified by column chromatography (MeOH/EtOAc = 30:70). Yield: 86.7 mg (85%); amorphous beige solid. Mp: 193–195 °C. Lit.:^19^ 194–196 °C. ^1^H NMR (CDCl_3_, 400 MHz) δ 8.97 (s, 1H), 8.32 (d, *J* = 8.0 Hz, 1H); 7.80 (d, *J* = 7.4 Hz, 1H); 7.64–7.57 (m, 2H); 7.45–7.40 (m, 3H); 7.29–7.25 (m, 2H); 7.07 (d, *J* = 8.4 Hz, 2H); 6.94 (d, *J* = 8.4 Hz, 2H). ^13^C{^1^H} NMR (101 MHz, CDCl_3_) δ 152.6, 138.1, 134.3, 133.4, 132.1, 131.4, 130.1, 129.9, 129.8, 129.7, 129.6, 129.3, 129.2, 128.9, 128.4, 127.8, 125.6. ^77^Se{^1^H} NMR (76 MHz, CDCl_3_) δ 338.6.

#### 3-Phenyl-4-(*p*-tolylselanyl)isoquinoline-2-oxide (**3e**)^19^

Purified by column chromatography (MeOH/EtOAc = 30:70). Yield: 84.4 mg (87%); amorphous orange solid. Mp: 191–192 °C. Lit.:^19^ 191–193 °C. ^1^H NMR (CDCl_3_, 400 MHz) δ 8.94 (s, 1H), 8.37 (d, *J* = 8.2 Hz, 1H), 7.70 (d, *J* = 7.4 Hz, 1H), 7.59–7.52 (m, 2H), 7.43–7.41 (m, 3H), 7.31–7.28 (m, 2H), 6.94–6.89 (m, 4H), 2.22 (s, 3H). ^13^C{^1^H} NMR (101 MHz, CDCl_3_) δ 152.3, 137.7, 137.1, 134.5, 131.5, 130.9, 130.2, 129.9, 129.7, 129.5, 129.1, 129.0, 128.4, 128.2, 127.9, 125.4, 21.1. ^77^Se{^1^H} NMR (76 MHz, CDCl_3_) δ 330.8.

#### 4-((4-Fluorophenyl)selanyl)-3-phenylisoquinoline-2-oxide (**3f**)^19^

Purified by column chromatography (MeOH/EtOAc = 30:70). Yield: 88.2 mg (90%); amorphous orange solid. Mp: 148–150 °C. Lit.:^19^ 146–148 °C. ^1^H NMR (CDCl_3_, 400 MHz) δ 8.93 (s, 1H), 8.40–8.35 (m, 1H), 7.72–7.68 (m, 1H), 7.60–7.55 (m, 2H), 7.43–7.39 (m, 3H), 7.28–7.25 (m, 2H), 7.01–6.96 (m, 2H), 6.81–6.75 (m, 2H). ^13^C{^1^H} NMR (101 MHz, CDCl_3_) δ 162.1 (d, ^1^*J*_C–F_ = 246.1 Hz), 151.2, 137.6, 134.2, 133.2 (d, ^3^*J*_C–F_ = 7.9 Hz), 131.2, 129.9, 129.7, 129.5, 129.0, 128.7, 128.3, 128.1, 125.8 (d, ^4^*J*_C–F_ = 3.3 Hz), 125.3, 116.5 (d, ^2^*J*_C–F_ = 21.7 Hz). ^77^Se{^1^H} NMR (76 MHz, CDCl_3_) δ 332.5.

#### 3-Phenyl-4-((3-(trifluoromethyl)phenyl)selanyl)isoquinoline-2-oxide (**3g**)^19^

Purified by column chromatography (MeOH/EtOAc = 30:70). Yield: 104 mg (94%); amorphous orange solid. Mp: 146–148 °C. Lit.:^19^ 145–147 °C. ^1^H NMR (CDCl_3_, 400 MHz) δ 8.97 (s, 1H), 8.36 (d, *J* = 8.2 Hz, 1H), 7.76 (d, *J* = 7.8 Hz, 1H), 7.66–7.59 (m, 2H), 7.42–7.36 (m, 4H), 7.26–7.22 (m, 3H), 7.20–7.11 (m, 2H). ^13^C{^1^H} NMR (101 MHz, CDCl_3_) δ 152.8, 138.1, 134.2, 134.1, 132.6, 131.6 (q, ^2^*J*_C–F_ = 32.3 Hz), 131.3, 130.1, 129.9, 129.82, 129.79, 129.3, 129.2, 128.7, 128.3, 127.6 (q, ^3^*J*_C–F_ = 3.9 Hz), 125.5, 124.0 (q, ^4^*J*_C–F_ = 3.4 Hz), 123.6 (q, ^1^*J*_C–F_ = 271.1 Hz). ^77^Se{^1^H} NMR (76 MHz, CDCl_3_) δ 345.9.

#### 4-(Naphthalen-2-ylselanyl)-3-phenylisoquinoline-2-oxide (**3h**)

Purified by column chromatography (MeOH/EtOAc = 30:70). Yield: 84.8 mg (80%); amorphous white solid. Mp: 155–158 °C. ^1^H NMR (CDCl_3_, 400 MHz) δ 8.96 (s, 1H), 8.31 (d, *J* = 8.4 Hz, 1H), 7.89 (d, *J* = 8.3 Hz, 1H), 7.80–7.60 (m, 3H), 7.58–7.36 (m, 4H), 7.32–7.24 (m, 3H), 7.21 (dd, *J* = 7.3, 2.3 Hz, 2H), 7.09 (t, *J* = 7.7 Hz, 1H), 6.99 (dd, *J* = 7.3, 0.9 Hz, 1H). ^13^C{^1^H} NMR (101 MHz, CDCl_3_) δ 171.2, 152.0, 137.9, 134.0, 133.9, 132.5, 131.6, 130.8, 130.0, 129.7, 129.6, 129.0, 128.9, 128.6, 128.4, 128.1, 127.8, 127.5, 126.8, 126.4, 126.0, 125.6. ^77^Se{^1^H} NMR (76 MHz, CDCl_3_) δ 295.0. HRMS *m*/*z*: [M + H]^+^ calcd for C_25_H_18_NOSe, 428.0554; found, 428.0545.

#### 4-(Mesitylselanyl)-3-phenylisoquinoline-2-oxide (**3i**)^19^

Purified by column chromatography (MeOH/EtOAc = 30:70). Yield: 84.2 mg (81%); amorphous orange solid. Mp: 140–142 °C. Lit.:^19^ 140–142 °C. ^1^H NMR (CDCl_3_, 400 MHz) δ 8.85 (s, 1H), 8.10 (d, *J* = 8.5 Hz, 1H), 7.68 (d, *J* = 8.2 Hz, 1H), 7.56–7.51 (m, 1H), 7.48–7.43 (m, 1H), 7.39–7.34 (m, 3H), 7.22–7.18 (m, 2H), 6.71 (s, 2H), 2.19 (s, 3H), 2.04 (s, 6H). ^13^C{^1^H} NMR (101 MHz, CDCl_3_) δ 149.3, 141.3, 138.3, 136.4, 134.1, 131.1, 130.5, 129.6, 129.3, 129.20, 129.17, 129.1, 128.7, 128.3, 127.3, 125.5, 24.0, 21.0. ^77^Se{^1^H} NMR (76 MHz, CDCl_3_) δ 270.3.

#### 3-(4-Chlorophenyl)-4-(*p*-tolylselanyl)isoquinoline-2-oxide (**3j**)

Purified by column chromatography (MeOH/EtOAc = 30:70). Yield: 119.5 mg (72%); amorphous reddish solid. Mp: 134–135 °C. ^1^H NMR (CDCl_3_, 400 MHz) δ 9.31 (s, 1H), 8.48 (d, *J* = 8.5 Hz, 1H), 8.01 (d, *J* = 8.0 Hz, 1H), 7.73 (t, *J* = 7.7 Hz, 1H), 7.63 (t, *J* = 7.2 Hz, 1H), 7.50 (d, *J* = 8.4 Hz, 2H), 7.36 (d, *J* = 8.4 Hz, 2H), 6.97–6.83 (m, 4H), 2.22 (s, 3H). ^13^C{^1^H} NMR (101 MHz, CDCl_3_) δ 157.1, 153.3, 140.6, 138.8, 136.4, 134.2, 133.1, 132.0, 131.4, 130.2, 130.0, 129.0, 128.8, 128.3, 128.3, 127.9, 127.8, 126.5, 122.1, 21.0. ^77^Se{^1^H} NMR (76 MHz, CDCl_3_) δ 293.3. HRMS *m*/*z*: [M + H]^+^ calcd for C_22_H_17_ClNOSe, 426.0164; found, 426.0158.

#### 3-(4-Chlorophenyl)-4-((4-chlorophenyl)selanyl)isoquinoline-2-oxide (**3k**)^19^

Purified by column chromatography (MeOH/EtOAc = 30:70). Yield: 88.8 mg (80%); amorphous beige solid. Mp: 156–159 °C. Lit.:^16^ 154–157 °C. ^1^H NMR (CDCl_3_, 400 MHz) δ 9.34 (s, 1H), 8.42 (d, J = 8.4 Hz, 1H), 8.04 (d, *J* = 8.0 Hz, 1H), 7.84–7.71 (m, 1H), 7.71–7.63 (m, 1H), 7.48 (d, J = 8.4 Hz, 2H), 7.37 (d, *J* = 8.4 Hz, 2H), 7.05 (d, *J* = 8.5 Hz, 2H), 6.92 (d, *J* = 8.5 Hz, 2H). ^13^C{^1^H} NMR (101 MHz, CDCl_3_) δ 157.3, 153.7, 140.4, 138.6, 134.5, 132.6, 132.2, 131.4, 131.2, 131.0, 129.5, 128.4, 128.4, 128.0, 121.6. ^77^Se{^1^H} NMR (76 MHz, CDCl_3_) δ 300.2.

#### 4-(Phenylselanyl)isoquinoline-22-oxide (**3l**)^19^

Purified by column chromatography (MeOH/EtOAc = 30:70). Yield: 42 mg (56%); amorphous yellow solid. Mp: 112–115 °C. Lit.:^19^ 110–113 °C. ^1^H NMR (CDCl_3_, 400 MHz) δ 8.64 (s, 1H), 8.17–8.03 (m, 1H), 7.97 (d, *J* = 1.6 Hz, 1H), 7.78–7.68 (m, 1H), 7.66–7.61 (m, 2H), 7.58 (dd, *J* = 7.8, 1.5 Hz, 2H), 7.44–7.32 (m, 3H). ^13^C{^1^H} NMR (101 MHz, CDCl_3_) δ 138.4, 135.3, 135.0, 130.8, 130.3, 130.0, 129.6, 129.4, 129.3, 129.2, 126.7, 126.0, 125.8. ^77^Se{^1^H} NMR (76 MHz, CDCl_3_) δ 349.9.

#### 4-(*p*-Tolylselanyl)isoquinoline-2-oxide (**3m**)

Purified by column chromatography (MeOH/EtOAc = 30:70). Yield: 54.6 mg (70%); amorphous reddish solid. Mp: 134–137 °C. ^1^H NMR (CDCl_3_, 400 MHz) δ 8.80 (s, 1H), 8.62 (s, 1H), 8.16 (d, *J* = 6.9 Hz, 1H), 8.12–7.97 (m, 1H), 7.84 (d, *J* = 1.6 Hz, 1H), 7.51 (d, *J* = 8.1 Hz, 2H), 7.19 (d, *J* = 7.7 Hz, 2H), 2.37 (s, 3H). ^13^C{^1^H} NMR (101 MHz, CDCl_3_) δ 140.0, 137.3, 135.7, 135.0, 131.2, 129.9, 129.6, 125.8, 125.6, 124.5, 122.3, 21.4. ^77^Se{^1^H} NMR (76 MHz, CDCl_3_) δ 345.4. HRMS *m*/*z*: [M + H]^+^ calcd for C_16_H_14_NOSe, 316.0235; found, 316.0233.

#### 4-((4-Chlorophenyl)selanyl)isoquinoline-2-oxide (**3n**)

Purified by column chromatography (MeOH/EtOAc = 30:70). Yield: 63 mg (76%); amorphous reddish solid. Mp: 154–157 °C. ^1^H NMR (CDCl_3_, 400 MHz) δ 8.67 (s, 1H), 8.14–7.92 (m, 2H), 7.73 (d, *J* = 5.1 Hz, 1H), 7.68–7.58 (m, 2H), 7.49 (d, *J* = 8.4 Hz, 2H), 7.32 (d, *J* = 8.4 Hz, 2H). ^13^C{^1^H} NMR (101 MHz, CDCl_3_) δ 138.8, 135.7, 130.5, 130.1, 129.8, 129.3, 129.3, 129.3, 126.0, 125.9, 125.2 ^77^Se{^1^H} NMR (76 MHz, CDCl_3_) δ 344.4. HRMS *m*/*z*: [M + H]^+^ calcd for C_15_H_11_ClNOSe, 335.9689; found, 335.9691.

#### 4-((3-(Trifluoromethyl)phenyl)selanyl)isoquinoline2-oxide (**3o**)

Purified by column chromatography (MeOH/EtOAc = 30:70). Yield: 86.5 mg (94%); amorphous reddish oil. ^1^H NMR (CDCl_3_, 400 MHz) δ 8.71 (s, 1H), 8.20–8.00 (m, 2H), 7.82 (s, 1H), 7.75 (d, *J* = 5.4 Hz, 1H), 7.70–7.56 (m, 1H), 7.44 (t, *J* = 7.8 Hz, 1H). ^13^C{^1^H} NMR (101 MHz, CDCl_3_) δ 139.9, 137.1, 136.3, 130.6, 131.6 (q, ^2^*J*_C–F_ = 32.3 Hz), 131.3, 130.1, 129.9, 129.8, 129.8, 129.3, 129.2, 128.7, 128.3, 127.6 (q, ^3^*J*_C–F_ = 3.9 Hz), 125.5, 124.0 (q, ^4^*J*_C–F_ = 3.4 Hz), 123.6 (q, ^1^*J*_C–F_ = 271.1 Hz). ^77^Se{^1^H} NMR (76 MHz, CDCl_3_) δ 351.2. HRMS *m*/*z*: [M + H]^+^ calcd for C_16_H_11_F_3_NOSe, 369.9953; found, 369.9952.

#### 3-Butyl-4-(phenylselanyl)isoquinoline-2-oxide (**3p**)

Purified by column chromatography (MeOH/EtOAc = 30:70). Yield: 64 mg (72%); orange oil. ^1^H NMR (CDCl_3_, 400 MHz) δ 8.89 (s, 1H), 8.34–8.29 (m, 1H), 7.69–7.64 (m, 1H), 7.54–7.49 (m, 2H), 7.17–7.14 (m, 5H), 3.57–3.52 (m, 2H), 1.68–1.60 (m, 2H), 1.47 (sext, *J* = 7.2 Hz, 2H), 0.93 (t, *J* = 7.2 Hz, 3H). ^13^C{^1^H} NMR (101 MHz, CDCl_3_) δ 155.7, 137.9, 131.9, 131.7, 129.9, 129.74, 129.68, 128.8, 128.7, 128.2, 126.9, 125.8, 125.3, 32.2, 29.9, 23.1, 14.0. ^77^Se{^1^H} NMR (76 MHz, CDCl_3_) δ 303.7. HRMS *m*/*z*: [M + H]^+^ calcd for C_19_H_20_NOSe, 358.0705; found, 358.0703.

#### 3-Butyl-4-(*p*-tolylselanyl)isoquinoline-2-oxide (**3q**)

Purified by column chromatography (MeOH/EtOAc = 30:70). Yield: 47.9 mg (52%); orange oil. ^1^H NMR (CDCl_3_, 400 MHz) δ 8.80 (d, *J* = 10.3 Hz, 1H), 8.36–8.20 (m, 1H), 7.72–7.51 (m, 1H), 7.45 (dd, *J* = 9.2, 6.1 Hz, 2H), 7.00 (d, *J* = 8.0 Hz, 2H), 6.90 (d, *J* = 8.0 Hz, 2H), 3.67–3.26 (m, 2H), 3.03–2.88 (m, 2H), 2.17 (s, 3H), 1.57 (dd, *J* = 15.1, 7.2 Hz, 2H), 0.86 (t, *J* = 7.3 Hz, 3H). ^13^C{^1^H} NMR (101 MHz, CDCl_3_) δ 155.4, 137.8, 137.0, 131.9, 130.4, 130.1, 129.8, 128.8, 126.1, 125.2, 124.6, 122.2, 32.1, 29.9, 29.1, 23.1, 14.0. ^77^Se{^1^H} NMR (76 MHz, CDCl_3_) δ 297.7. HRMS *m*/*z*: [M + H]^+^ calcd for C_20_H_22_NOSe, 372.0867; found, 372.0856.

#### 3-Butyl-4-((4-chlorophenyl)selanyl)isoquinoline-2-oxide (**3r**)

Purified by column chromatography (MeOH/EtOAc = 30:70). Yield: 76.6 mg (79%); orange oil. ^1^H NMR (CDCl_3_, 400 MHz) δ 8.99 (s, 1H), 8.34 (dd, *J* = 6.1, 3.3 Hz, 1H), 7.89–7.65 (m, 1H), 7.62 (dd, *J* = 6.3, 3.1 Hz, 2H), 7.33–6.84 (m, 4H), 3.78–3.41 (m, 2H), 1.86–1.66 (m, 2H), 1.56 (q, *J* = 7.3 Hz, 2H), 1.02 (t, *J* = 7.3 Hz, 3H). ^13^C{^1^H} NMR (101 MHz, CDCl_3_) δ 155.7, 138.0, 133.0, 131.5, 130.9, 130.0, 129.8, 128.8, 128.4, 128.2, 125.3, 122.1, 32.1, 29.9, 23.0, 13.9. ^77^Se{^1^H} NMR (76 MHz, CDCl_3_) δ 303.6. HRMS *m*/*z*: [M + H]^+^ calcd for C_19_H_19_ClNOSe, 392.0320; found, 392.0318.

#### 4-(Benzylselanyl)-3-phenylisoquinoline-2-oxide (**3s**)

Purified by column chromatography (MeOH/EtOAc = 30:70). Yield: 59.6 mg (61%) red oil. ^1^H NMR (CDCl_3_, 400 MHz) δ 8.90 (s, 1H), 8.52–8.26 (m, 1H), 7.78–7.68 (m, 1H), 7.69–7.54 (m, 2H), 7.43–7.44 (m, 4H), 7.12–7.09 (m, 6H), 6.75 (d, *J* = 7.7 Hz, 2H). ^13^C{^1^H} NMR (101 MHz, CDCl_3_) δ 152.6, 137.5, 134.6, 131.9, 130.1, 129.7, 129.4, 128.9, 128.9, 128.8, 128.8, 128.5, 128.16, 127.3, 125.5, 33.4. ^77^Se{^1^H} NMR (76 MHz, CDCl_3_) δ 304.9. HRMS *m*/*z*: [M + H]^+^ calcd for C_22_H_18_NOSe, 392.0554; found, 392.0559.

#### 3-Phenyl-4-(phenylselanyl)isoquinolin-1(2*H*)-one (**4**)

Purified by column chromatography (hexanes/EtOAc = 70:30). Yield: 59.2 mg (63%); amorphous pale white solid. Mp: 130–133 °C. ^1^H NMR (CDCl_3_, 400 MHz) δ 7.94 (dd, *J* = 8.1, 1.4 Hz, 2H), 7.70–7.64 (m, 2H), 7.64–7.57 (m, 3H), 7.56–7.44 (m, 2H), 7.43–7.22 (m, 6H). ^13^C{^1^H} NMR (101 MHz, CDCl_3_) δ 157.6, 142.9, 139.6, 136.9, 136.6, 135.7, 134.9, 134.6, 134.5, 134.1, 133.3, 127.4, 133.1, 132.1, 130.5. ^77^Se{^1^H} NMR (76 MHz, CDCl_3_) δ 215.0. HRMS *m*/*z*: [M + H]^+^ calcd for C_21_H_16_NOSe, 378.0392; found, 378.0389.

#### 12-Benzoyl-6-phenyl-7-(phenylselanyl)-13*H*-isoquinolino[2,1-*a*]quinolin-13-one (**6**)

Purified by column chromatography (hexanes/EtOAc = 75:25). Yield: 82.6 mg (57%); orange oil. ^1^H NMR (DMSO-*d*_6_, 400 MHz) δ 9.49 (s, 1H), 8.33 (d, *J* = 8.5 Hz, 1H), 8.24 (d, *J* = 8.1 Hz, 1H), 7.87–7.79 (m, 1H), 7.74 (q, *J* = 7.1 Hz, 2H), 7.58 (dd, *J* = 7.2, 2.3 Hz, 3H), 7.56–7.51 (m, 1H), 7.47–7.39 (m, 5H), 7.12 (q, *J* = 6.0 Hz, 4H), 7.00 (dd, *J* = 7.7, 1.7 Hz, 3H). ^13^C{^1^H} NMR (101 MHz, DMSO-*d*_6_) δ 158.0, 153.5, 146.5, 141.8, 137.5, 133.2, 132.6, 132.1, 129.9, 129.5, 129.0, 128.6, 128.0, 127.9, 127.7, 127.4, 126.2, 120.2. ^77^Se{^1^H} NMR (76 MHz, DMSO-*d*_6_) δ 293.9. HRMS *m*/*z*: [M + H]^+^ calcd for C_36_H_24_NO_2_Se, 582.0967; found, 582.0964.

## Data Availability

The data underlying this study are available in the published article and its Supporting Information.

## Supporting Information Available

Copies of ^1^H, ^13^C{^1^H}, and ^77^Se{^1^H} spectra of the synthesized compounds (PDF)
FAIR data, including the primary NMR FID files, for compounds **3a**–**s**, **4**, and **6** (ZIP)
