# Supplementary material for: Synthesis of Selenium-Decorated N-Oxide Isoquinolines: Arylseleninic Acids in Selenocyclization Reactions
Source: J Org Chem. 2024 Aug 1;89(16):11272–80. doi: 10.1021/acs.joc.4c00944 (PMC11334173; doi:10.1021/acs.joc.4c00944)
Supplement: Supplementary file 1 — jo4c00944_si_001.pdf [file jo4c00944_si_001.pdf]

SUPPORTING INFORMATION

Synthesis of selenium-decorated *N*-oxide isoquinolines: Arylseleninic acids in selenocyclization reactions

João M. Anghinoni,[a] Sabrina S. Ferreira,[a] Jean C. Kazmierczak,[b] Gelson Perin,[a] Filipe Penteado,*[,b] and Eder J. Lenardão*[,c]

[a] *Centro de Ciências Químicas, Farmacêuticas e de Alimentos – CCQFA, Universidade Federal de Pelotas – UFPel. P. O. box 354, 96010-900, Pelotas – RS (Brazil). E-mail: lenardao@ufpel.edu.br*

[b] *Centro de Ciências Exatas e Naturais, Departamento de Química, Universidade Federal de Santa Maria Av. Roraima, Building 18, 97105-340, Santa Maria – RS (Brazil). E-mail: filipe.penteado@ufsm.br*

Table of Contents

1. General Information……..S2

2. General procedures for the synthesis of starting materials **1a-g** and **2a-h**..........................S3

3. References...S3

4. Copies of ^{1}H, $^{13}C\{1H\}$, and $^{77}Se\{^{1}H\}$ NMR spectra..S5

1. General information

The reactions were monitored by TLC carried out on precoated TLC sheets ALUGRAM® Xtra SIL G/UV254 by using UV light as visualization agent. Merck silica gel (particle size 63-200 μm) was used to flash chromatography, and PTLC Glass Plates L × W 20 cm × 20 cm, silica gel 60 F254, 1 mm, was used in the preparative thin layer chromatography. Hydrogen nuclear magnetic resonance spectra (^{1}H NMR) were obtained at 400 MHz on A Bruker Ascend 400 spectrometer. The spectra were recorded in $CDCl_3$ solutions. The chemical shifts are reported in ppm, referenced to tetramethysilane (TMS) as the external reference. Hydrogen coupling patterns are described as singlet (s), doublet (d), triplet (t), doublet of doublets (dd) and multiplet (m). Coupling constants (J) are reported in Hertz. Carbon-13 nuclear magnetic resonance spectra (^{13}C NMR) were obtained at 100 MHz on Bruker Nuclear Ascend 400 spectrometer. The chemical shifts are reported in ppm, referenced to the solvent peak of $CDCl_3$. Selenium-77 nuclear magnetic resonance spectra (^{77}Se NMR) were obtained at 76 MHz on Bruker Nuclear Ascend 400 spectrometer. The HRMS analyses were performed in a HESI Quadrupole-Orbitrap (Q extractive focus, Thermo Scientific) spectrometer equipped with an APCI source operating in positive mode. The samples were solubilized in methanol and analyzed by direct infusion at a constant flow rate. The acquisition parameters were: Scan type Full MS; resolution 70000; polarity positive. Ionization conditions HESI: Sheath gas 20; aux gas 10; spray voltage 2.8kV; capillary temperature 300 °C. The mass-to-charge ratio (m/z) data were processed and analyzed using Bruker Daltonics softwares: Compass Data Analysis and Isotope Pattern.

2. General procedures for the Synthesis of Starting Materials 1a-g and 2a-h.[1,2]

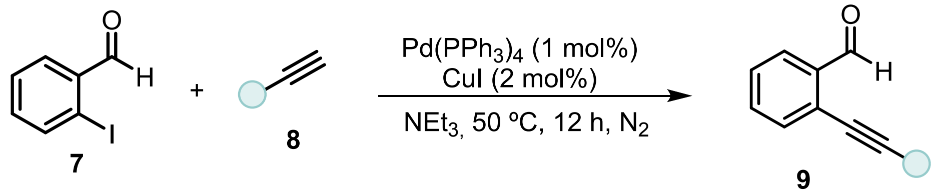

Under a nitrogen atmosphere, 2′-iodobenzaldehyde **7** (2.46 g, 10 mmol), arylacetylene **8** (1.12 g, 11 mmol), $Pd(PPh_3)_4$ (116 mg, 0.1 mmol), CuI (38 mg, 0.2 mmol), and triethylamine (20.0 mL) were added in sequence, and then stirred at 50 ℃ under oil bath for 12 h. Subsequently, the reaction mixture was quenched with an aqueous solution of saturated NH_4Cl and extracted with ethyl acetate. The organic layer was washed with water and brine, then dried over Na_2SO_4, filtered, and concentrated under reduced pressure. The crude product **9** was further purified by column chromatography.

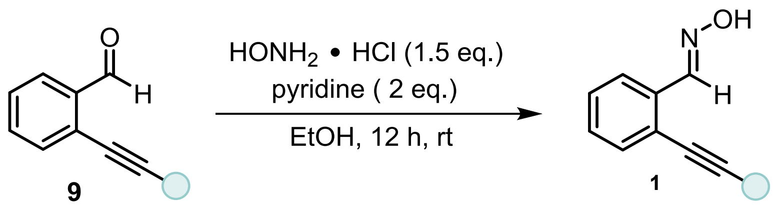

(*E*)-2-(Phenylethynyl)benzaldehyde oxime **9** (4 mmol) was first dissolved in ethanol (8.0 mL). Then, pyridine (633 mg, 8 mmol) and hydroxylamine hydrochloride (417 mg, 6 mmol) were added to the resulting solution subsequently and the mixture was stirred at room temperature for 12 h. Subsequently, the reaction mixture was extracted with ethyl acetate. The organic layer was washed with water and brine, then dried over Na_2SO_4, filtered, and concentrated under reduced pressure. The crude product **1** was further purified through column chromatography.

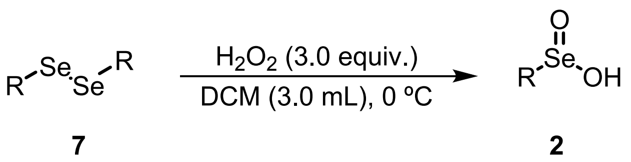

In a round-bottomed flask were added the diphenyl diselenide derivatives **7** (0.6 mmol) and DCM (3.0 mL), being the resulting mixture cooled with an ice bath (0 °C). Then, H_2O_2 (3.0 equiv) was added dropwise. The resulting mixture was stirred at 0 °C until the

formation of a white suspension and the disappearance of the yellowish solution. After, the solvent was evaporated, removing DCM and residual water, being the precipitate washed several times with hexanes and dried in the oven. The freshly dried white solid was directly employed in the next reaction step. WARNING: EXPLOSIVE CONTENT IF SUBMITTED TO VACUUM PUMP.

3. References

1. Zhang, L.; Xiong, W.; Yao, B.; Liu, H.; Li, M.; Qin, Y.; Yu, Y.; Li, X.; Chen, M.; Wu, W.; Li, J.; Wang, J.; Jiang, H. Facile Synthesis of Isoquinolines and Isoquinoline N-Oxides via a Copper-catalyzed Intramolecular Cyclization in Water, *RSC Adv.* **2022**, *12*, 30248-30252.
2. Syper; L.; Młochowski, J. A Convenient Oxidation of Halomethylarenes and Alcohols to Aldehydes with Dimethyl Selenoxide and Potassium Benzeneselenite, *Synthesis 1984*, *1984*, 747-752.

4. Copies of ^{1}H, ^{13}C{^{1}H}, and ^{77}Se{^{1}H} NMR spectra

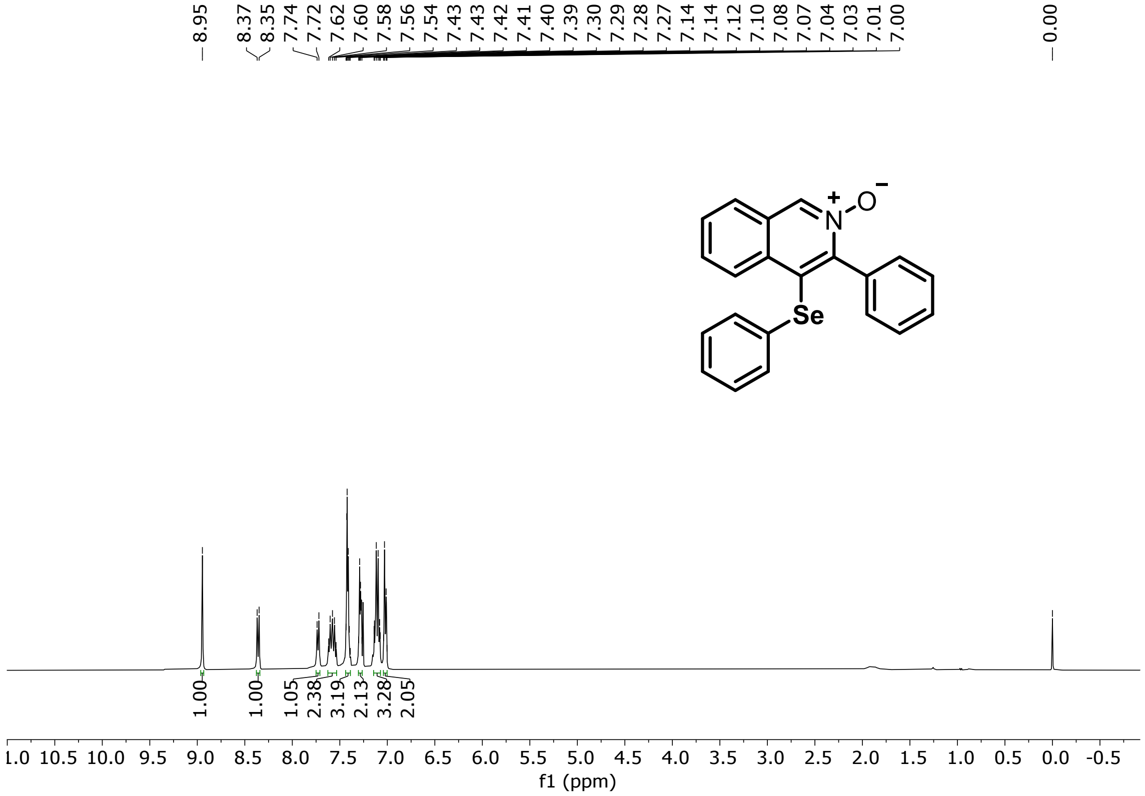

Figure S1. ^{1}H NMR (400 MHz, $CDCl_3$) spectrum of the compound **3a**.

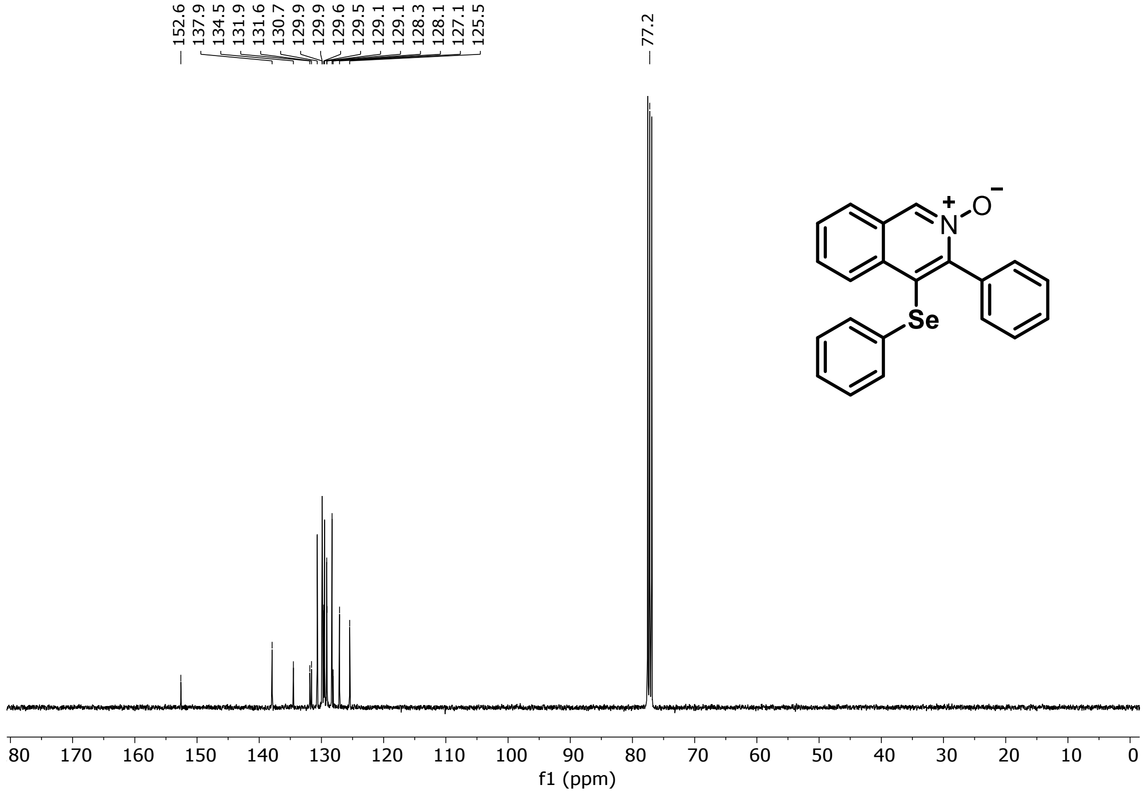

Figure S2. ^{13}C{^{1}H} NMR (100 MHz, $CDCl_3$) spectrum of the compound **3a**.

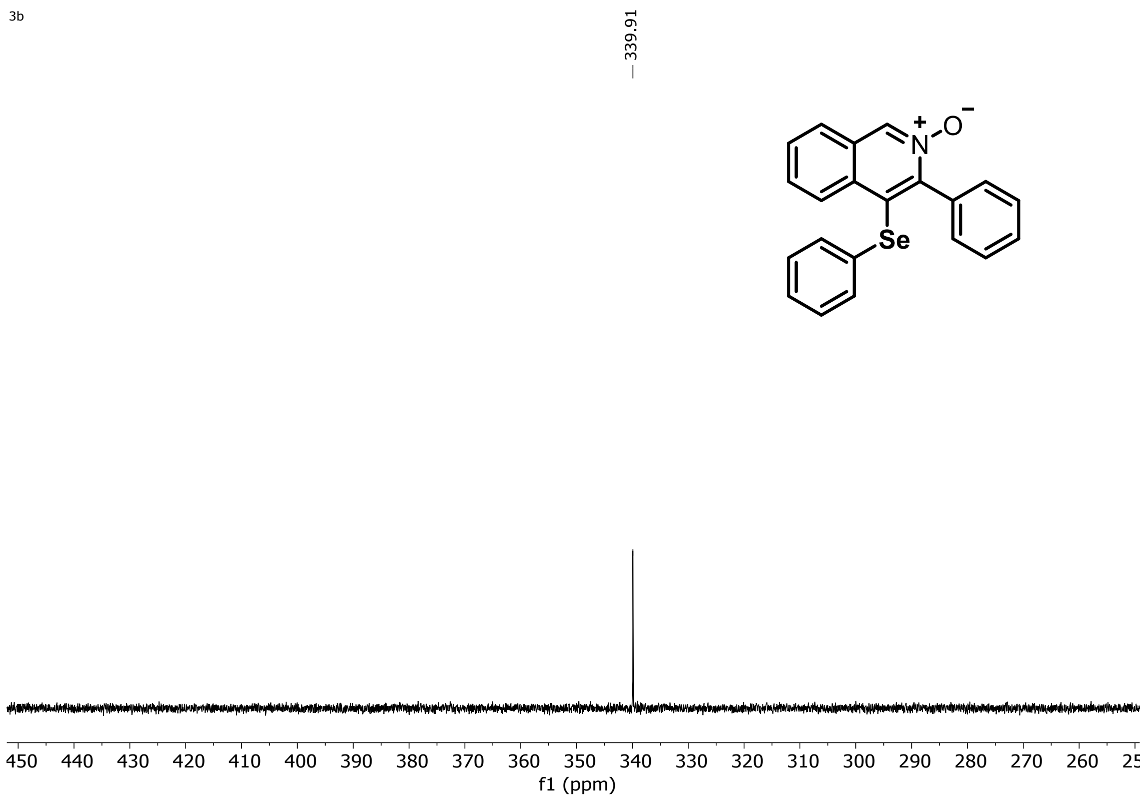

Figure S3. $^{77}Se\{^{1}H\}$ NMR (76 MHz, $CDCl_3$) spectrum of the compound **3a**.

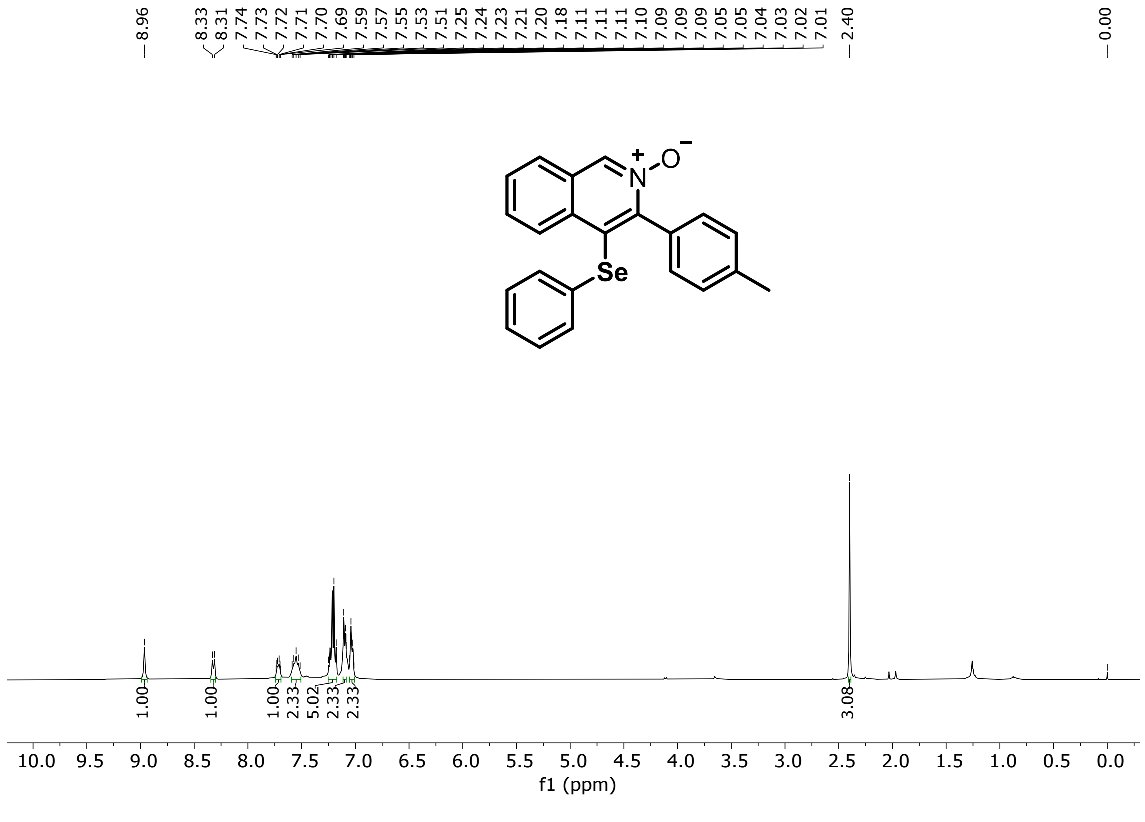

Figure S4. ^{1}H NMR (400 MHz, $CDCl_3$) spectrum of the compound **3b**.

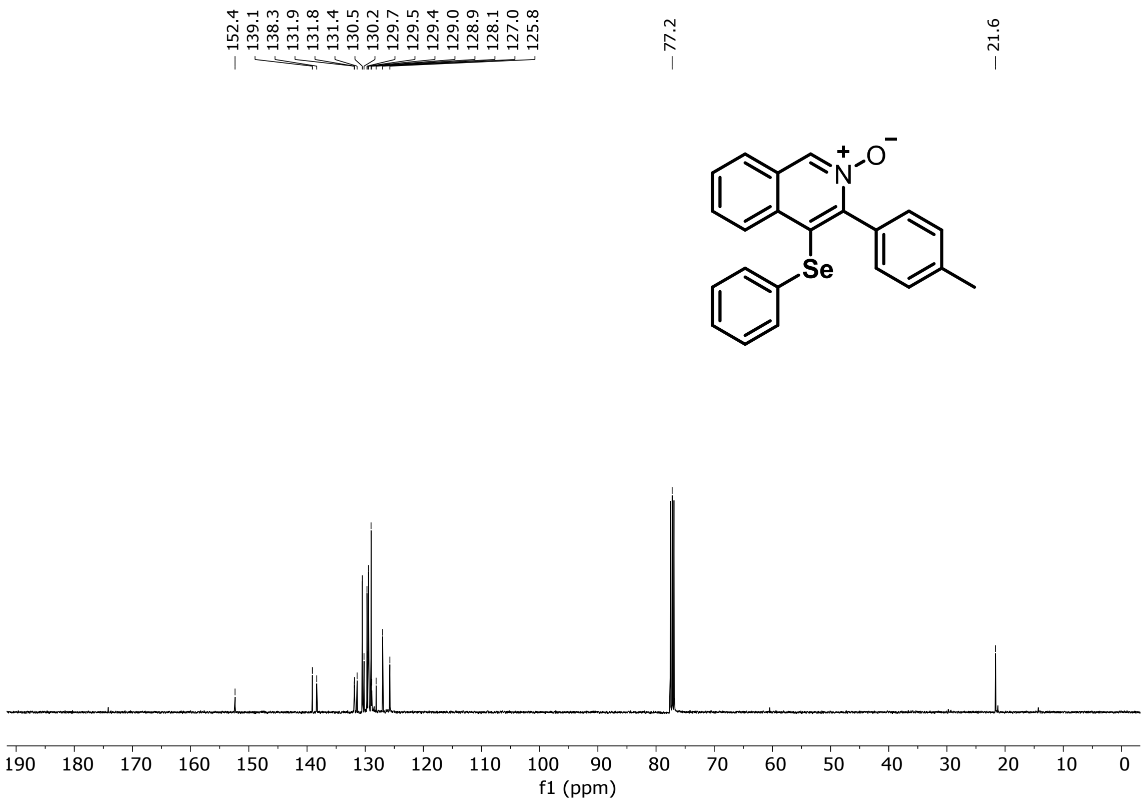

Figure S5. $^{13}C\{^{1}H\}$ NMR (100 MHz, $CDCl_3$) spectrum of the compound **3b**.

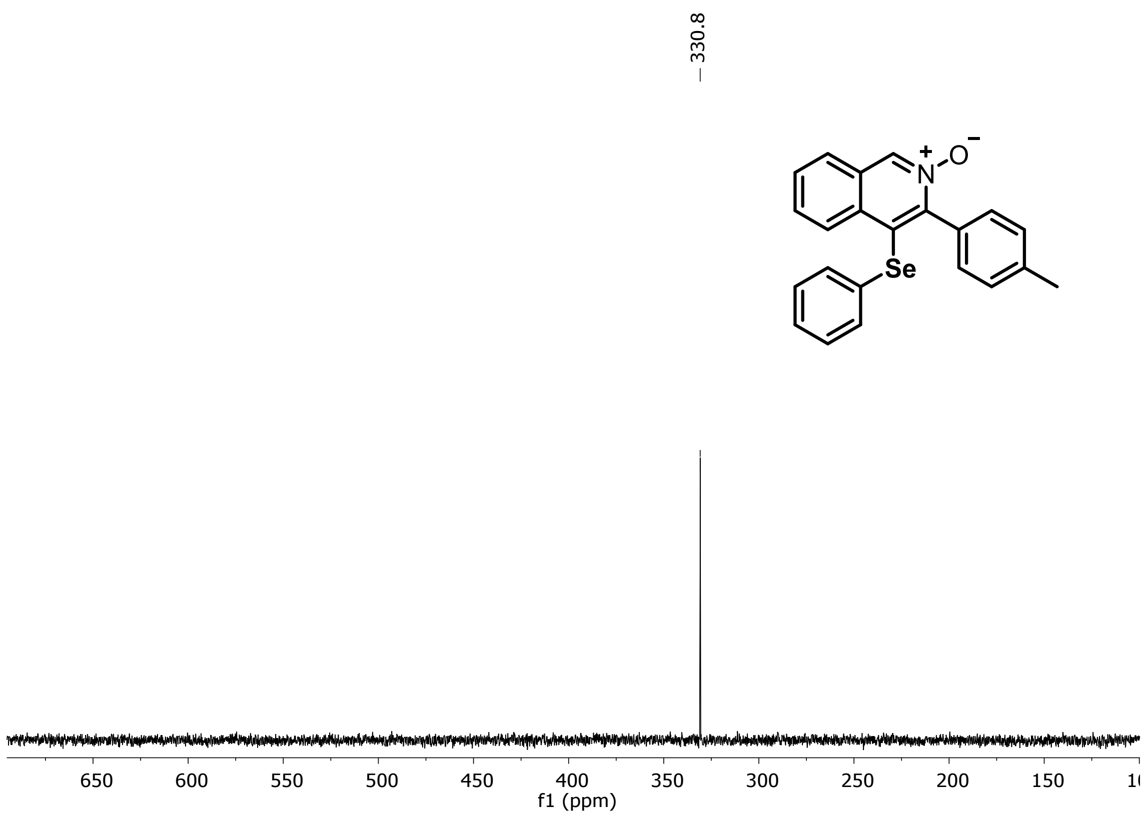

Figure S6. $^{77}Se\{^{1}H\}$ NMR (76 MHz, $CDCl_3$) spectrum of the compound **3b**.

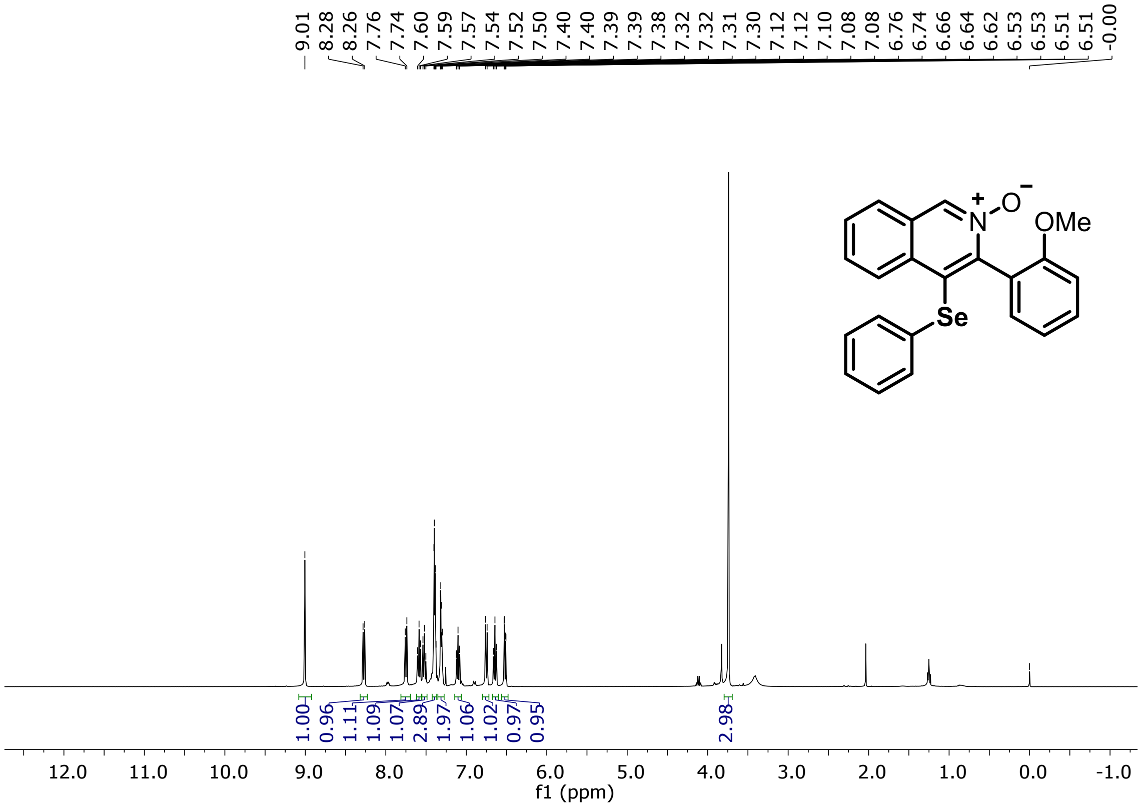

Figure S7. ^{1}H NMR (400 MHz, $CDCl_3$) spectrum of the compound **3c**.

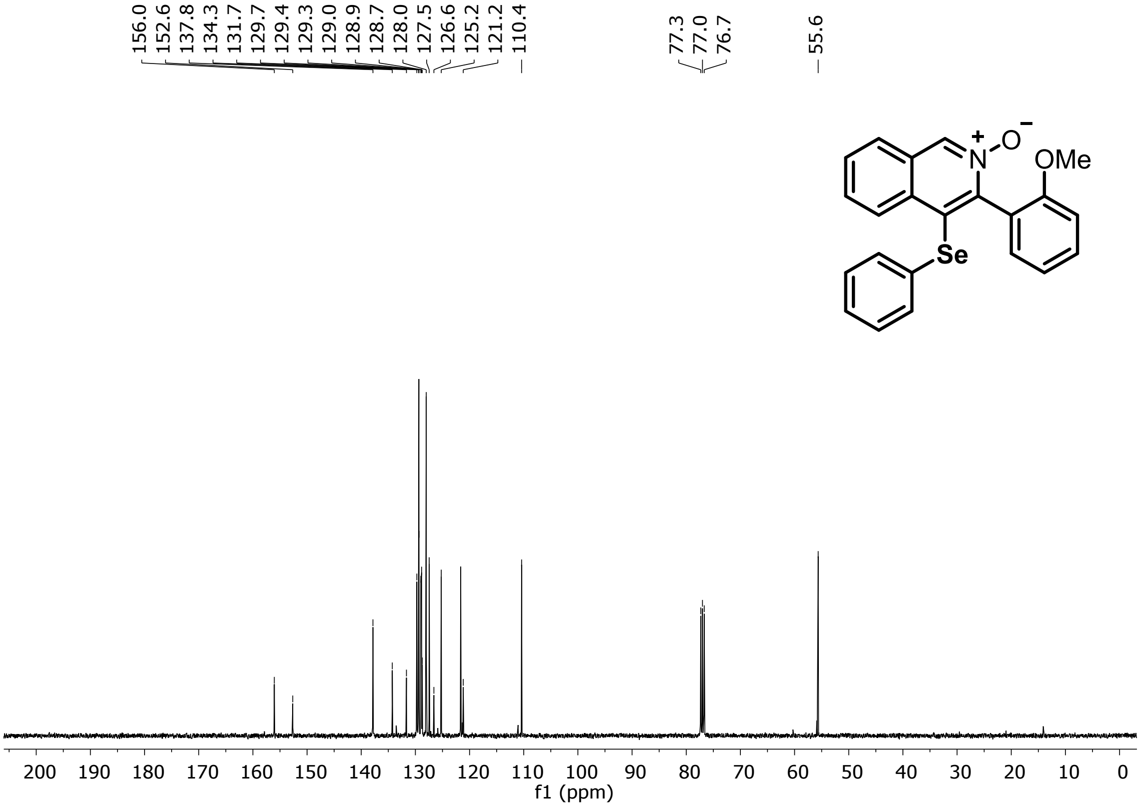

Figure S8. $^{13}C\{^{1}H\}$ NMR (100 MHz, $CDCl_3$) spectrum of the compound **3c**.

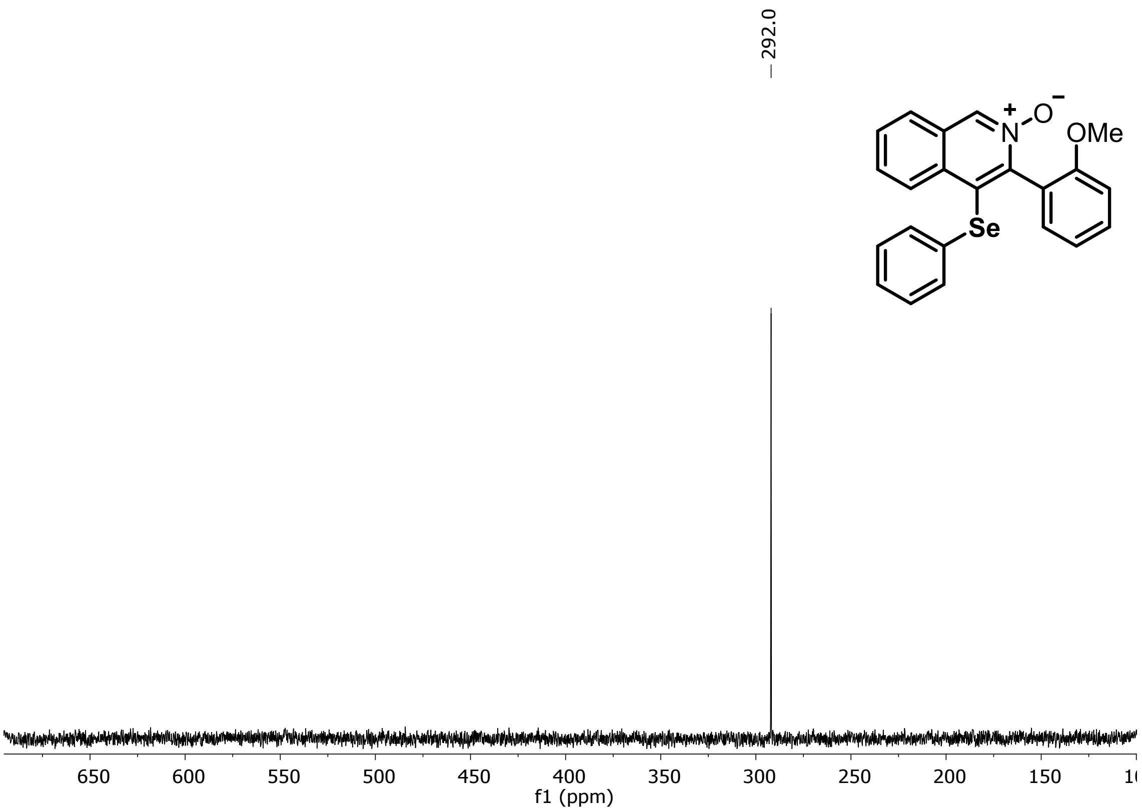

Figure S9. $^{77}Se\{^1H\}$ NMR (76 MHz, $CDCl_3$) spectrum of the compound **3c**.

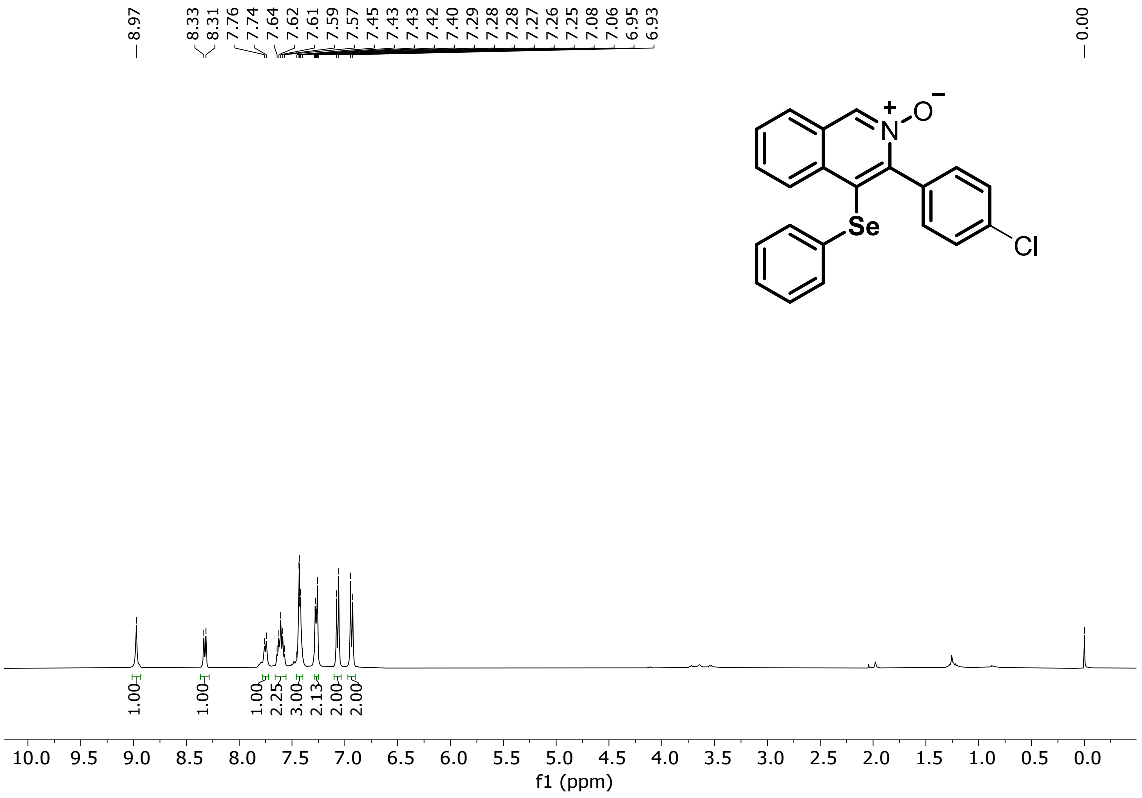

Figure S10. ^1H NMR (400 MHz, $CDCl_3$) spectrum of the compound **3d**.

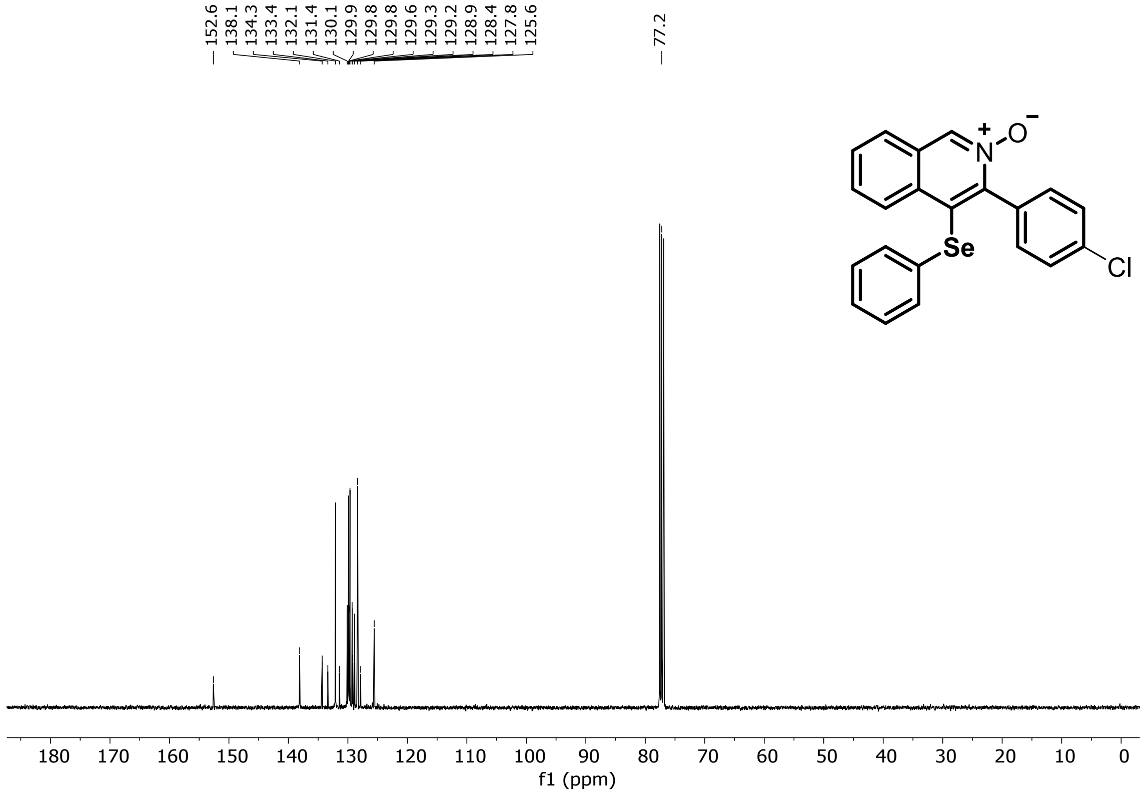

Figure S11. $^{13}C\{^{1}H\}$ NMR (100 MHz, $CDCl_3$) spectrum of the compound **3d**.

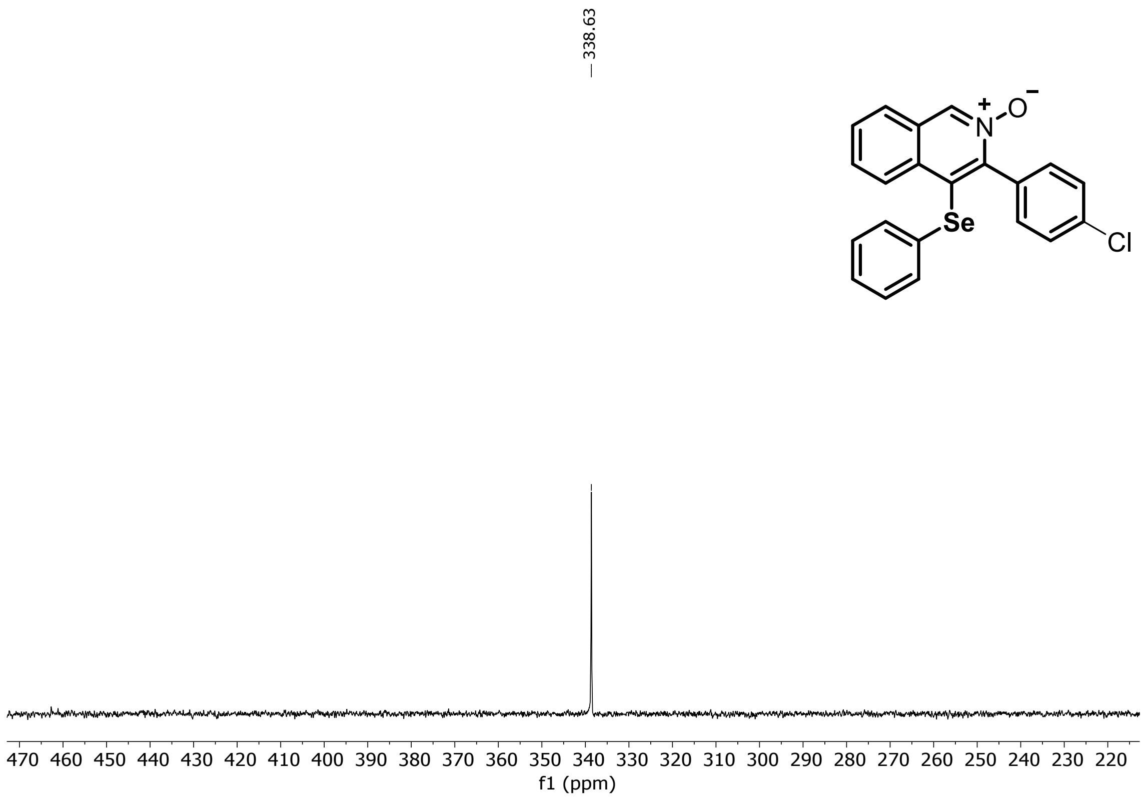

Figure S12. $^{77}Se\{^{1}H\}$ NMR (76 MHz, $CDCl_3$) spectrum of compound **3d**.

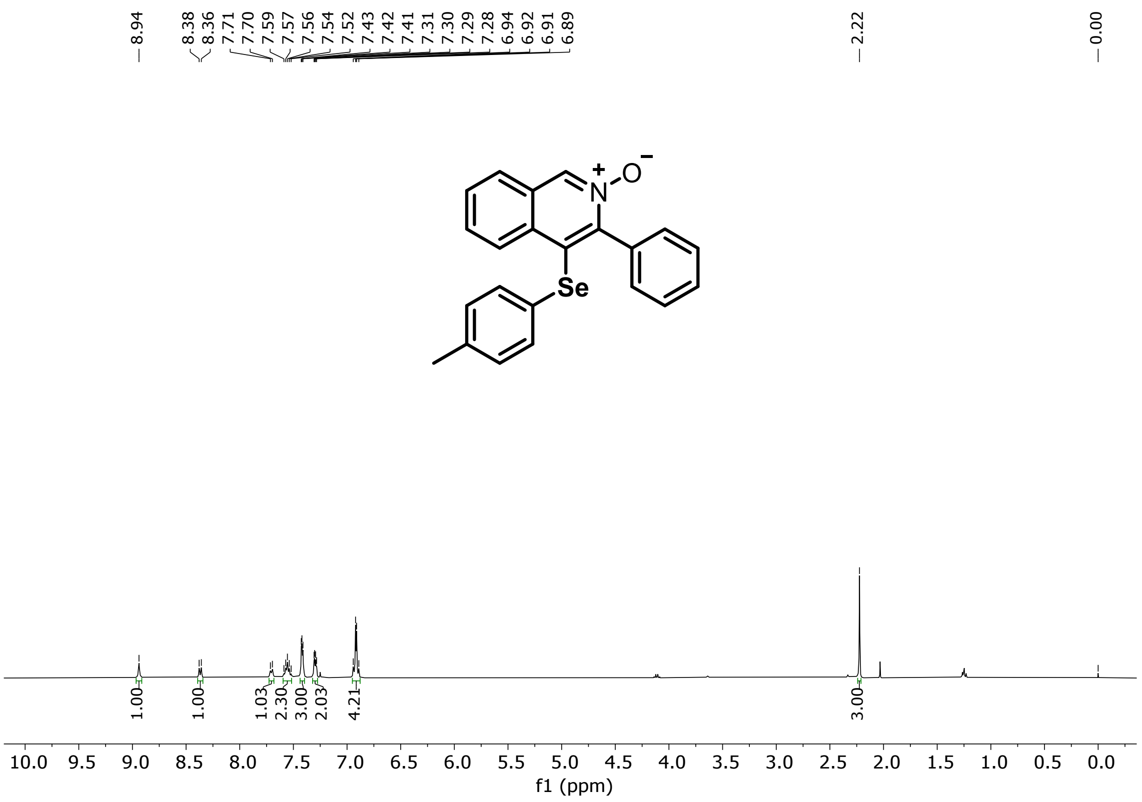

Figure S13. ^{1}H NMR (400 MHz, $CDCl_3$) spectrum of the compound **3e**.

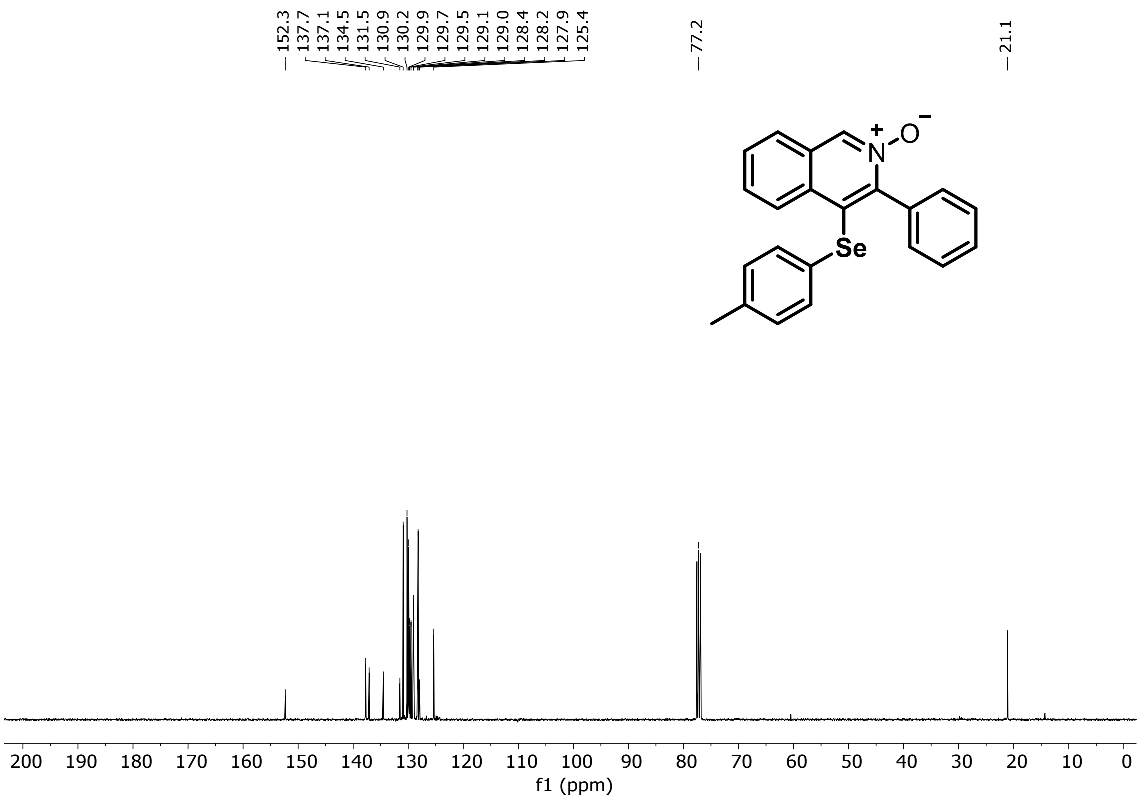

Figure S14. $^{13}C\{^{1}H\}$ NMR (100 MHz, $CDCl_3$) spectrum of the compound **3e**.

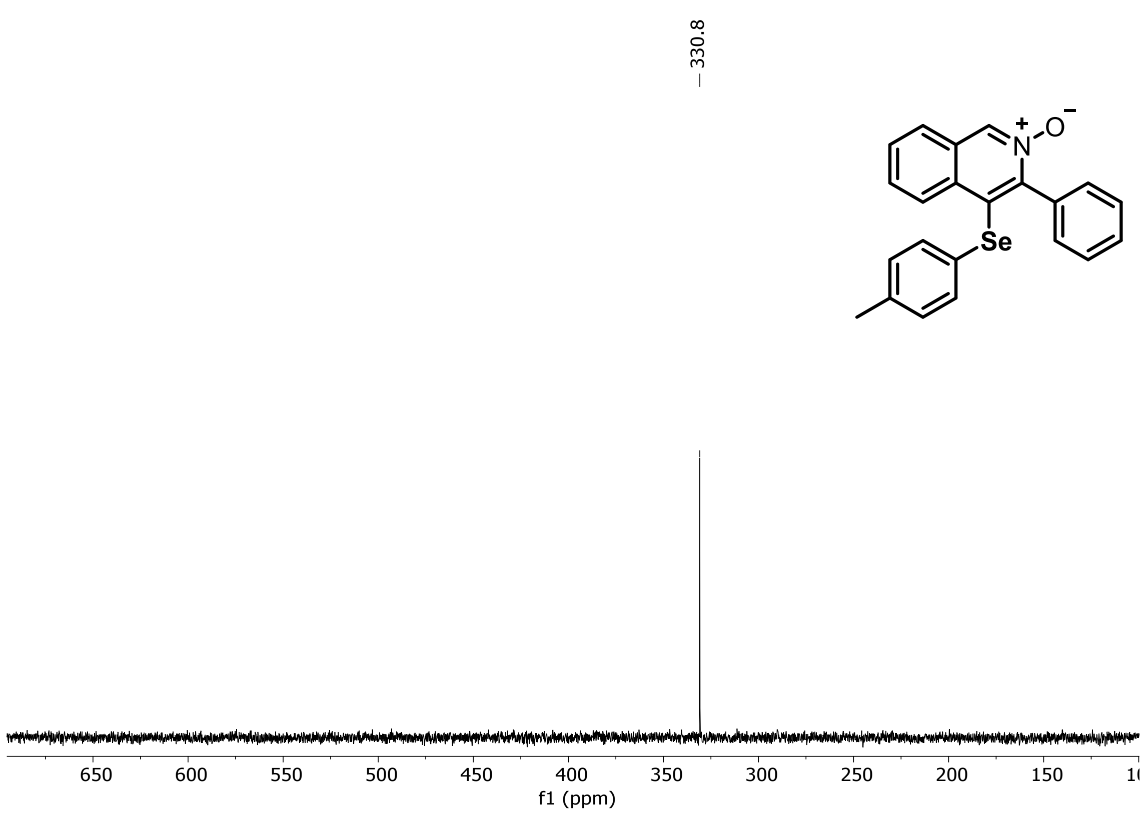

Figure S15. $^{77}Se\{^1H\}$ NMR (76 MHz, $CDCl_3$) spectrum of the compound **3e**.

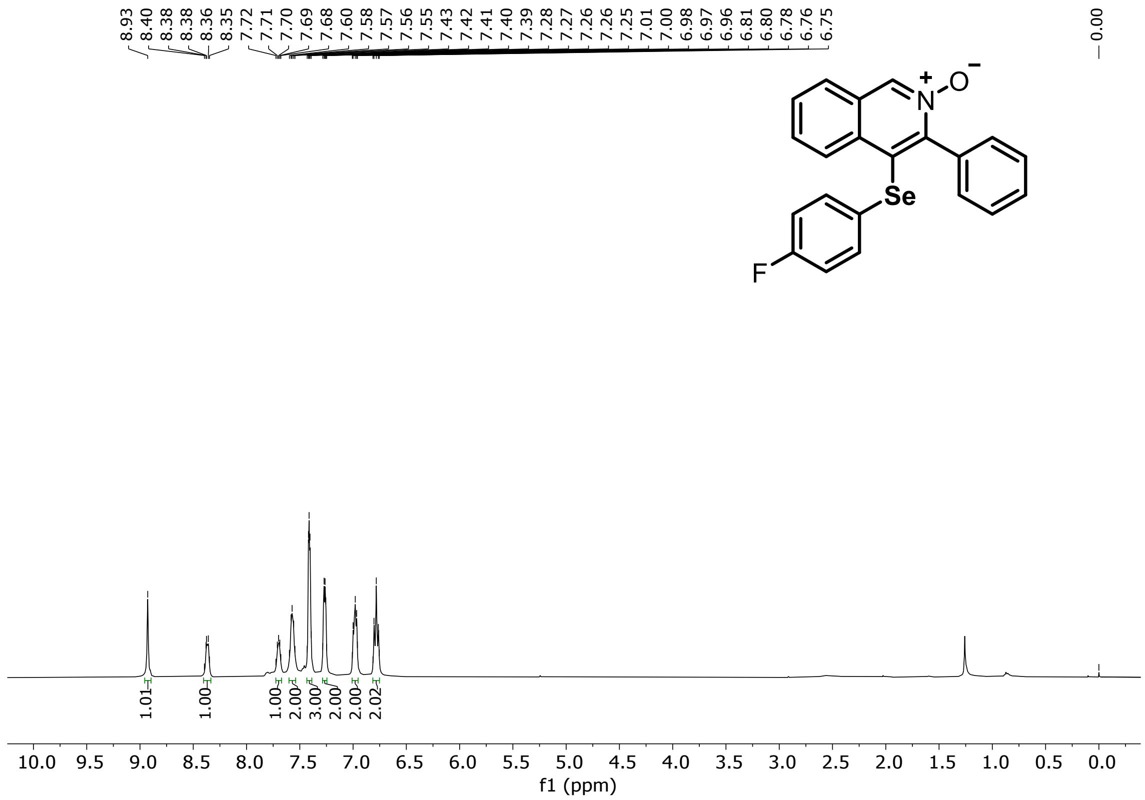

Figure S16. ^1H NMR (400 MHz, $CDCl_3$) spectrum of the compound **3f**.

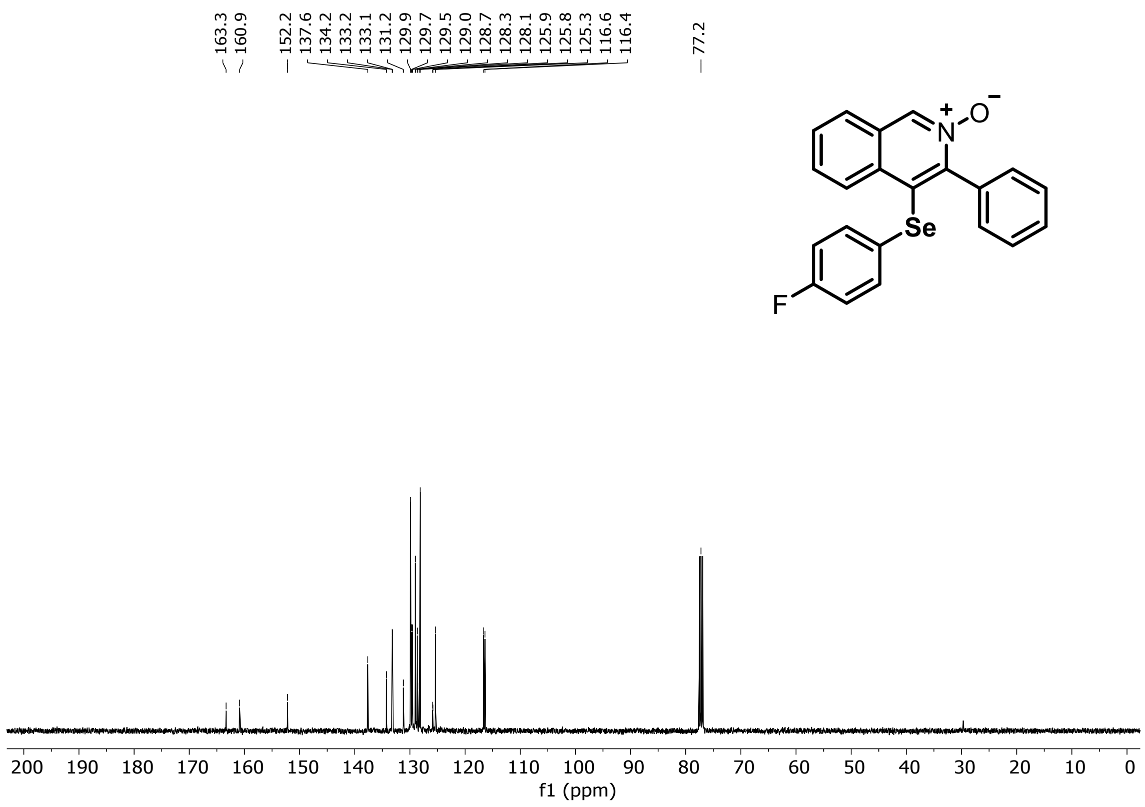

Figure S17. $^{13}C\{^{1}H\}$ NMR (100 MHz, $CDCl_3$) spectrum of the compound **3f**.

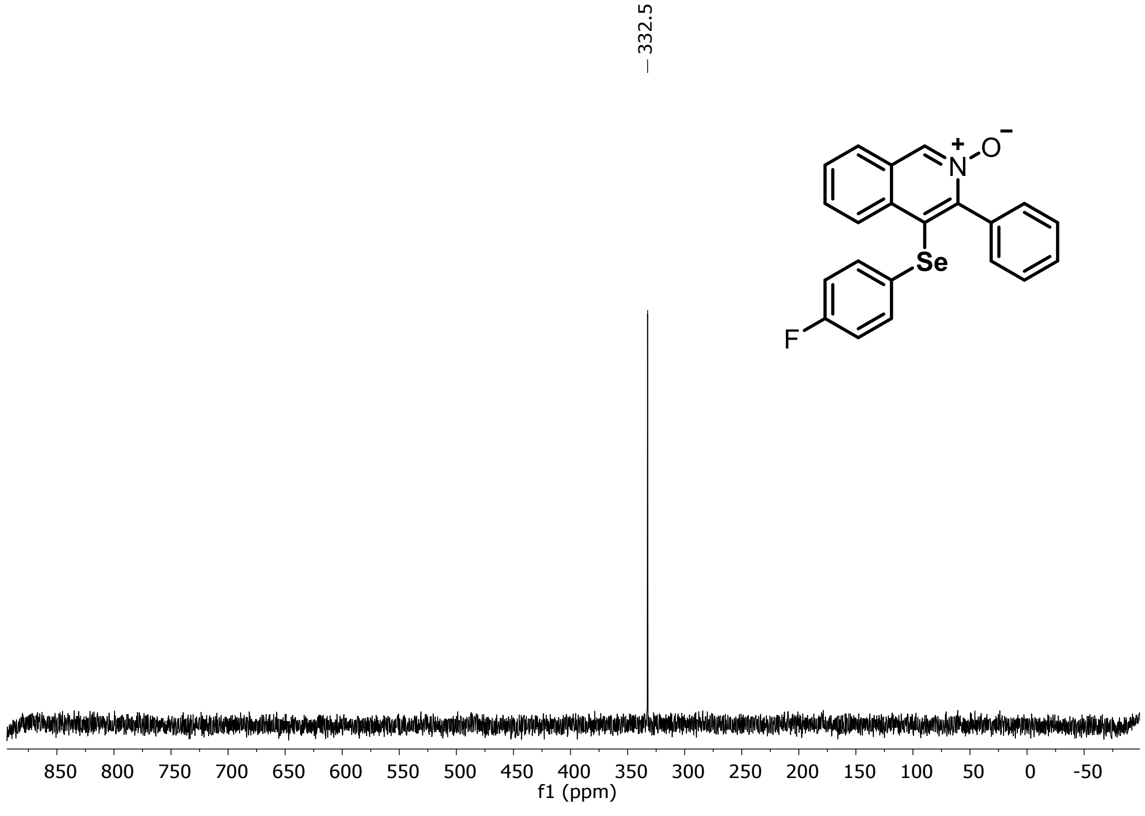

Figure S18. $^{77}Se\{^{1}H\}$ NMR (76 MHz, $CDCl_3$) spectrum of the compound **3f**.

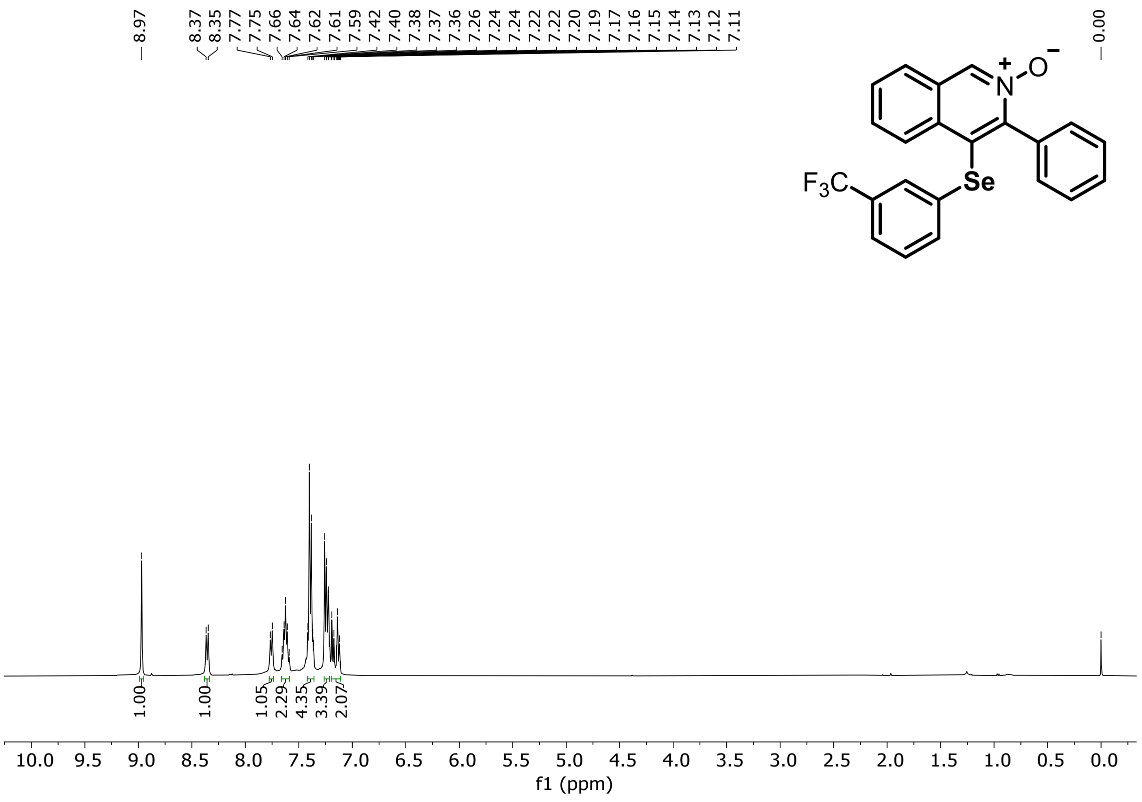

Figure S19. ^{1}H NMR (400 MHz, $CDCl_3$) spectrum of the compound **3g**.

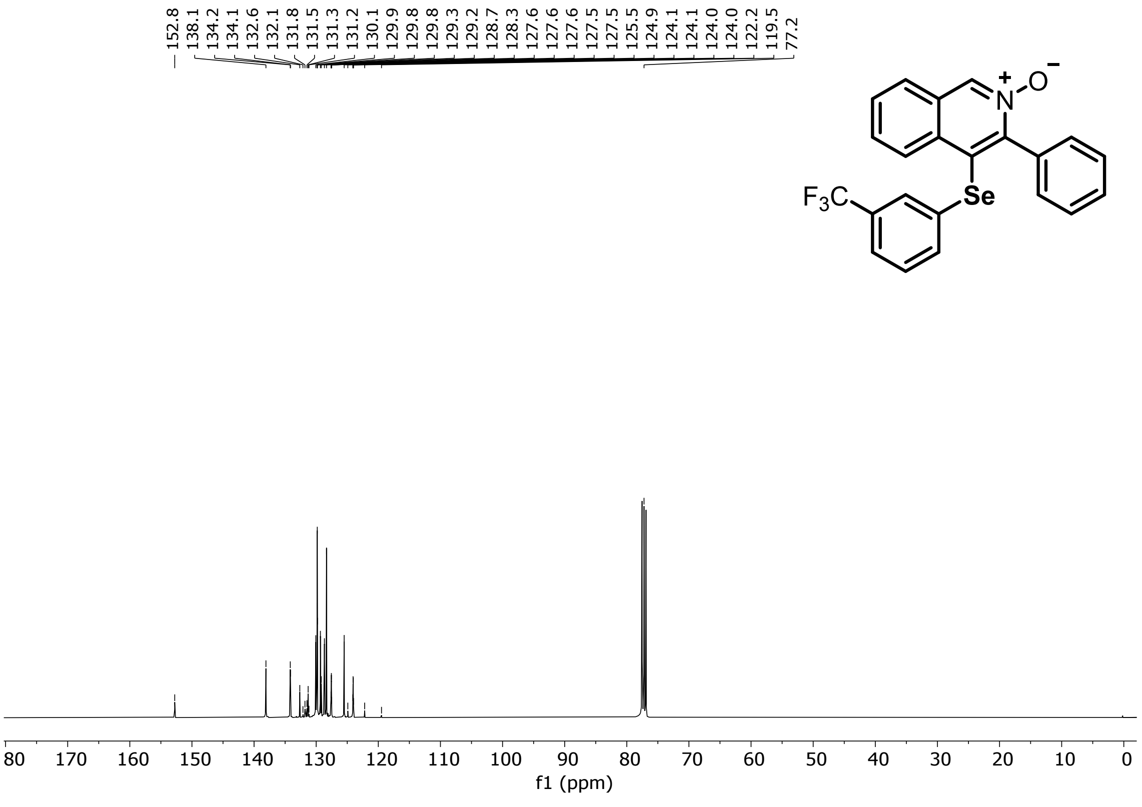

Figure S20. $^{13}C\{^{1}H\}$ NMR (100 MHz, $CDCl_3$) spectrum of the compound **3g**.

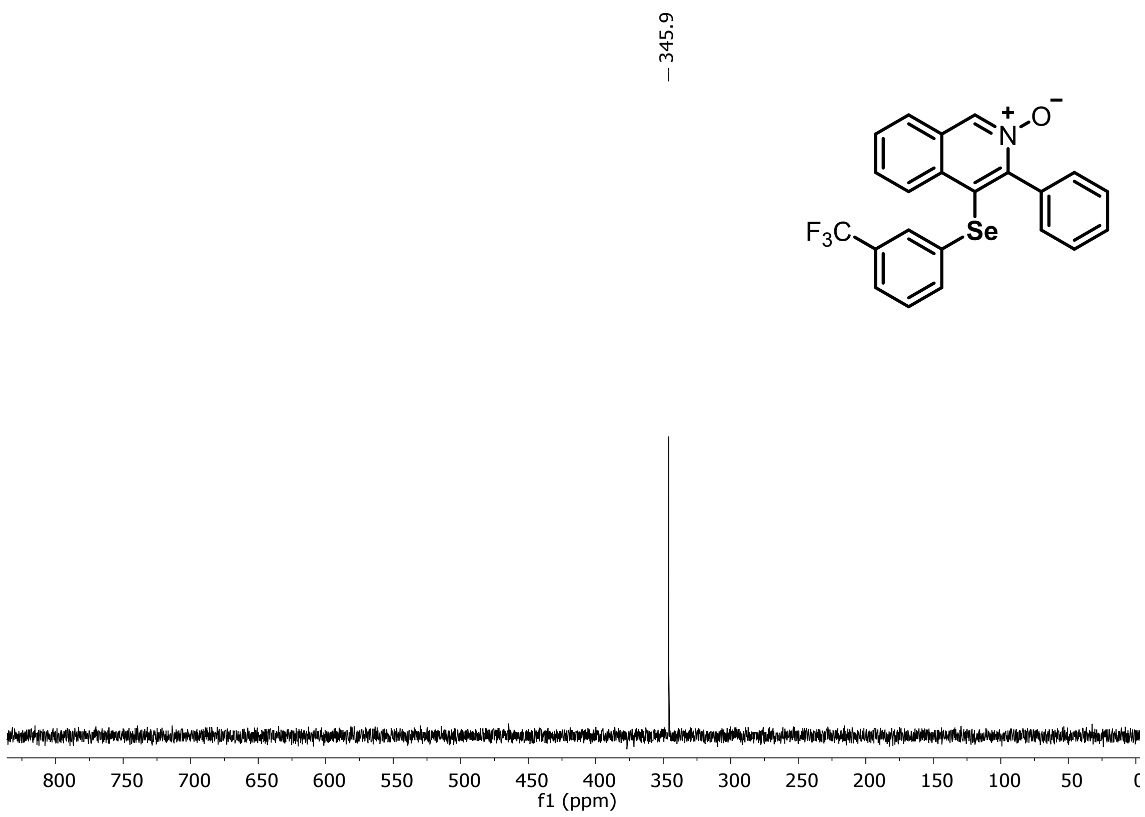

Figure S21. $^{77}Se\{^{1}H\}$ NMR (76 MHz, $CDCl_3$) spectrum of the compound **3g**.

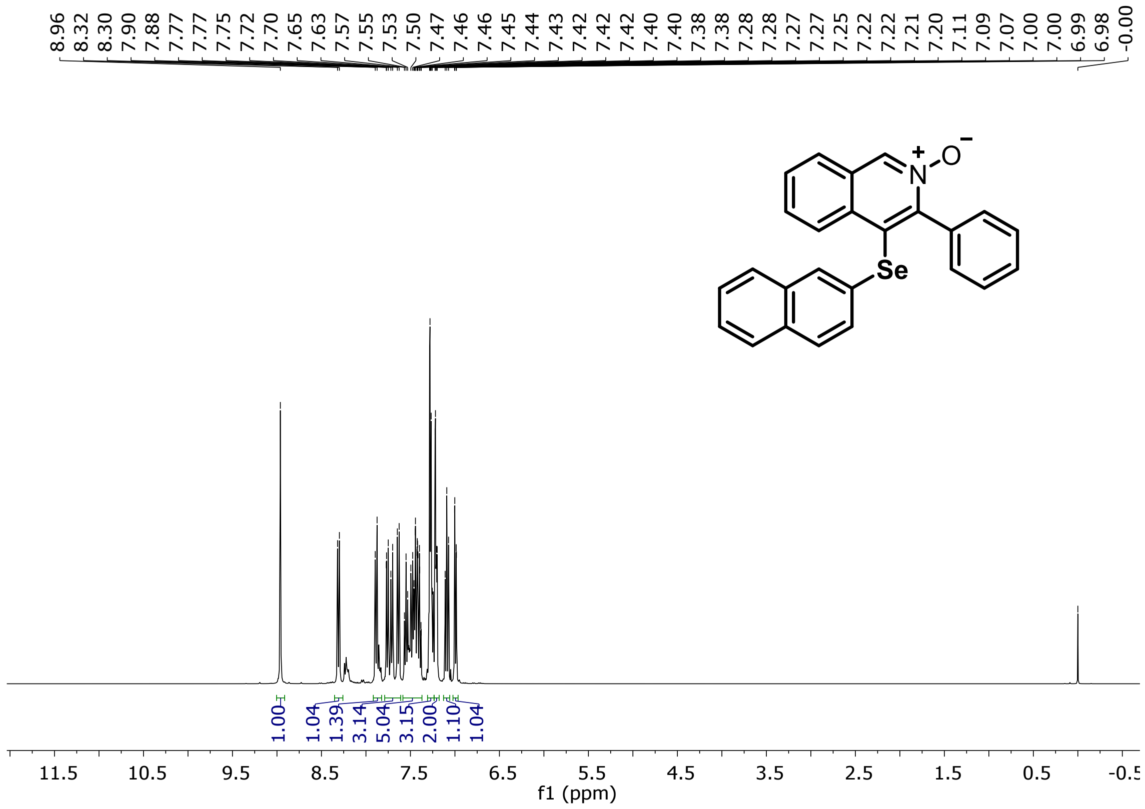

Figure S22. ^{1}H NMR (400 MHz, $CDCl_3$) spectrum of the compound **3h**.

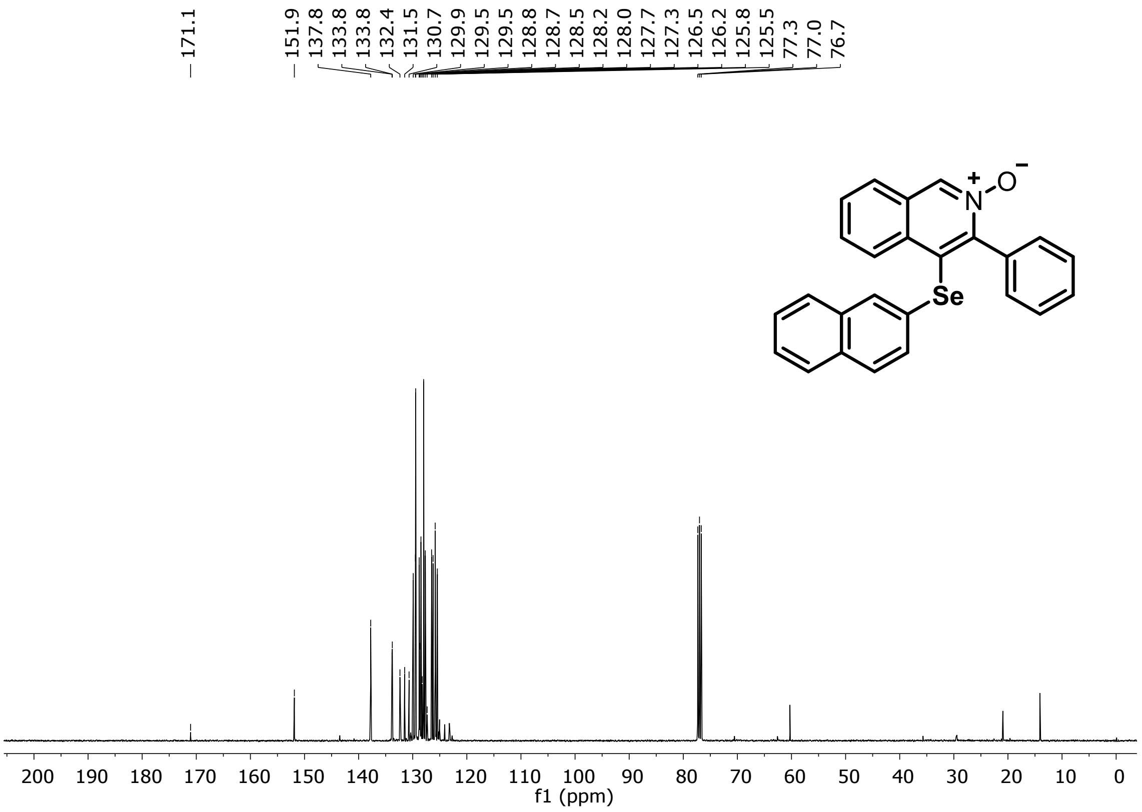

Figure S23. $^{13}C\{^1H\}$ NMR (100 MHz, $CDCl_3$) spectrum of the compound **3h**.

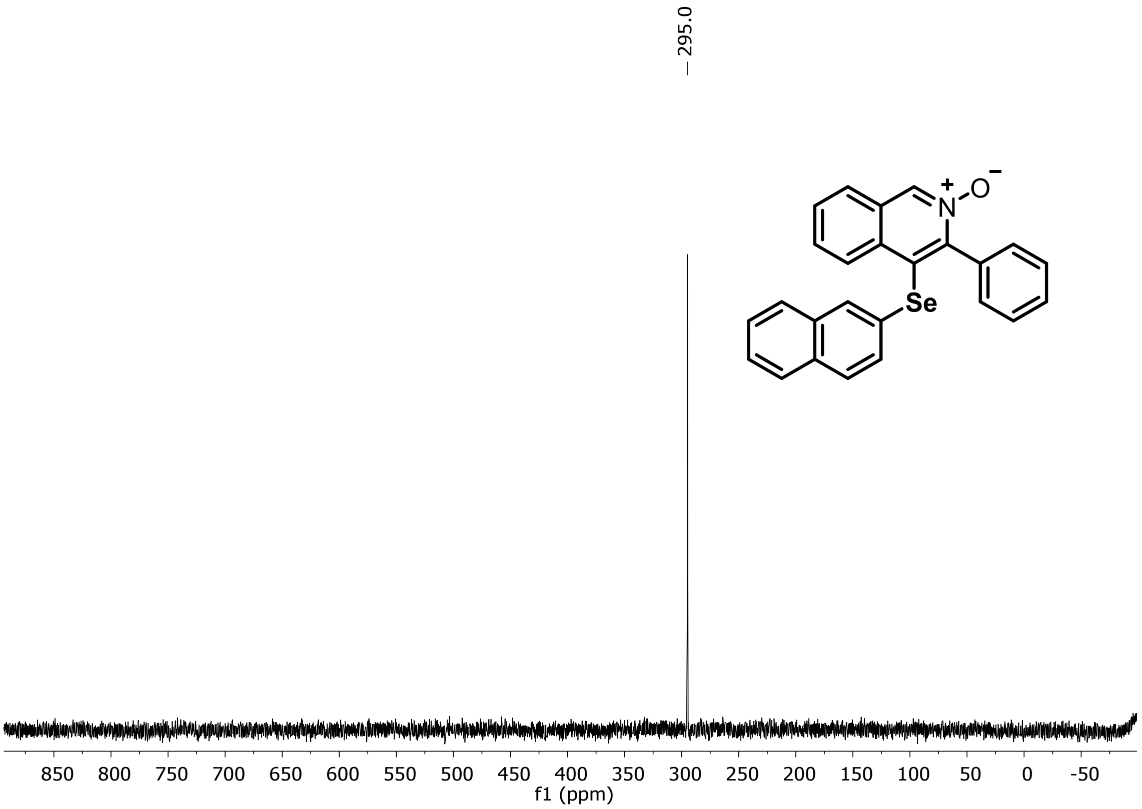

Figure S24. $^{77}Se\{^1H\}$ NMR (76 MHz, $CDCl_3$) spectrum of the compound **3h**.

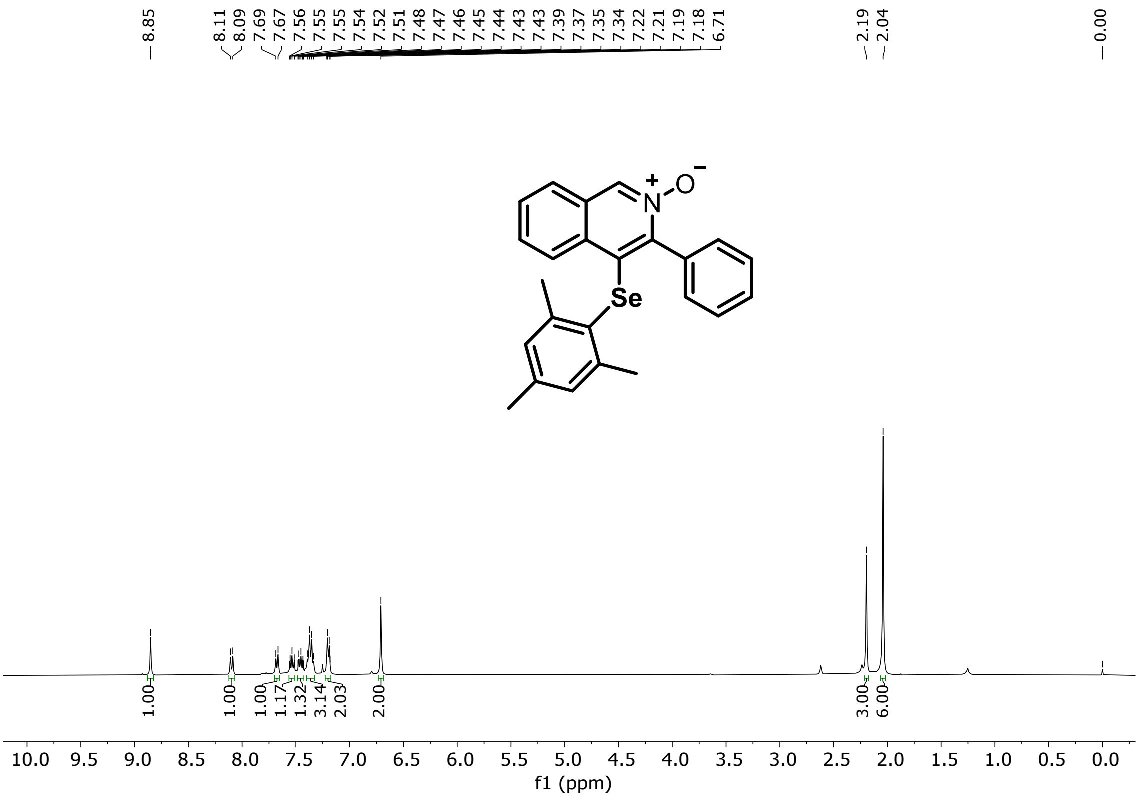

Figure S25. ^{1}H NMR (400 MHz, $CDCl_3$) spectrum of the compound **3i**.

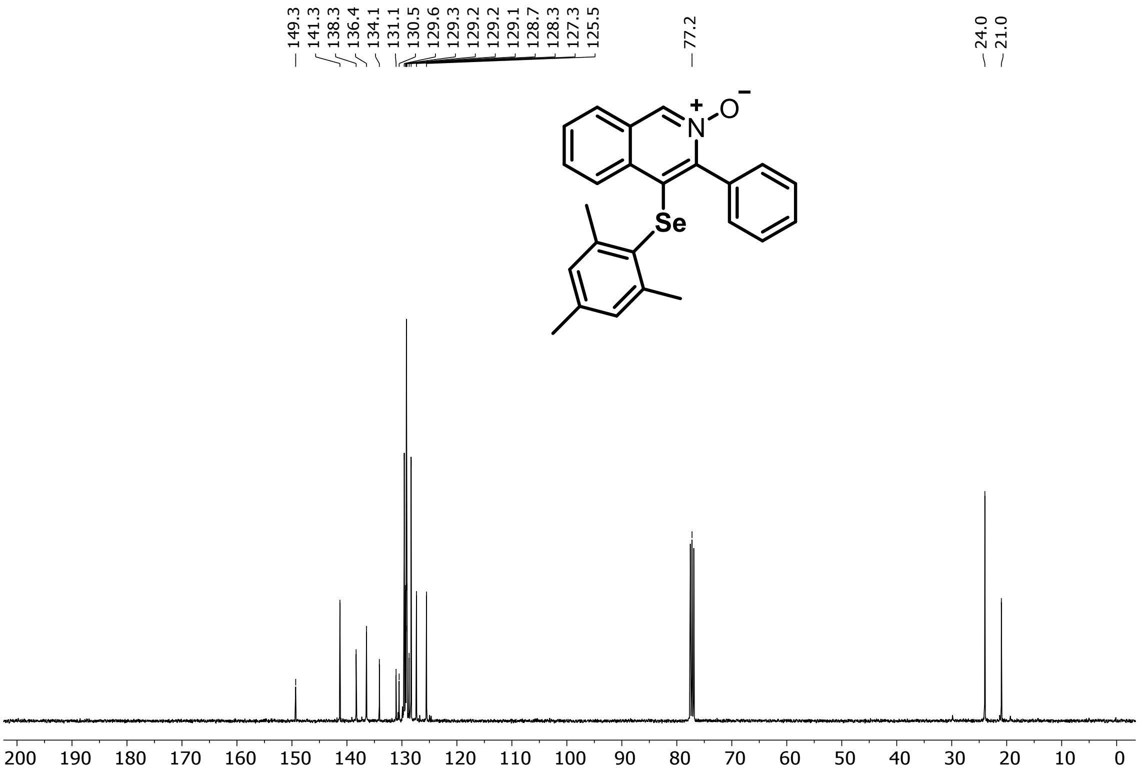

Figure S26. $^{13}C\{^{1}H\}$ NMR (100 MHz, $CDCl_3$) spectrum of the compound **3i**.

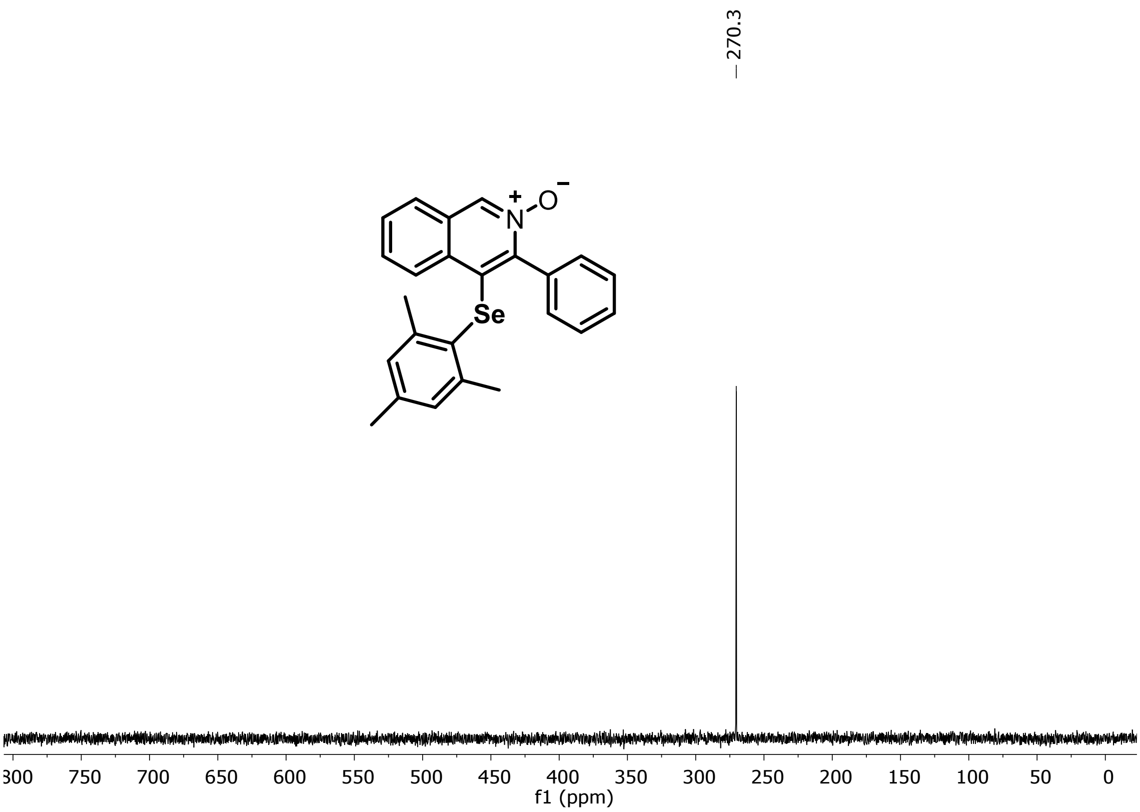

Figure S27. $^{77}Se\{^{1}H\}$ NMR (76 MHz, $CDCl_3$) spectrum of the compound **3i**.

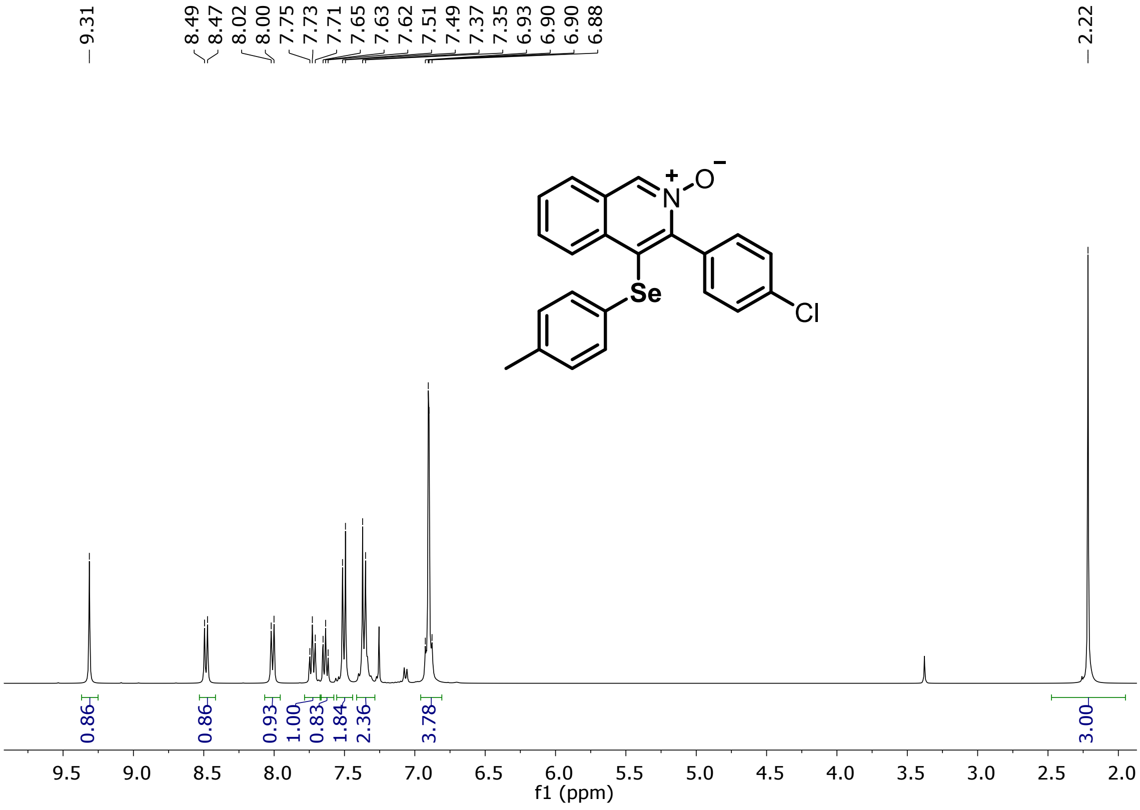

Figure S28. ^{1}H NMR (400 MHz, $CDCl_3$) spectrum of the compound **3j**.

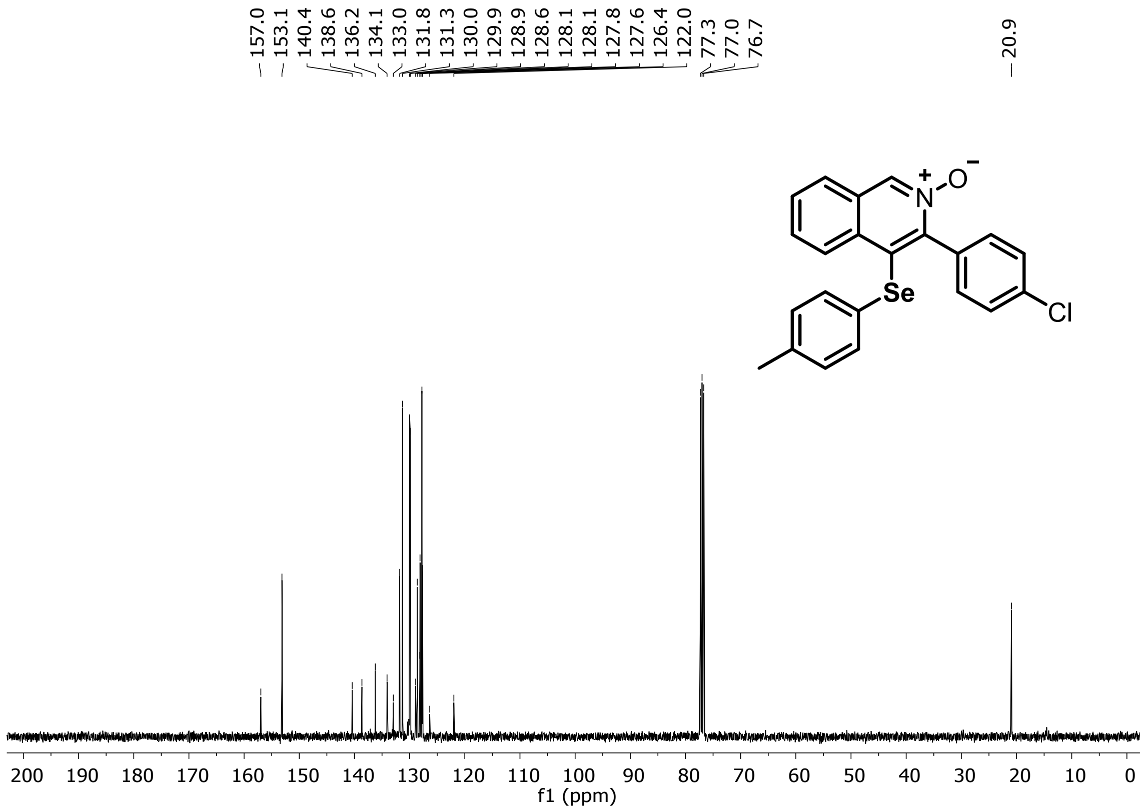

Figure S29. $^{13}C\{^{1}H\}$ NMR (100 MHz, $CDCl_3$) spectrum of the compound **3j**.

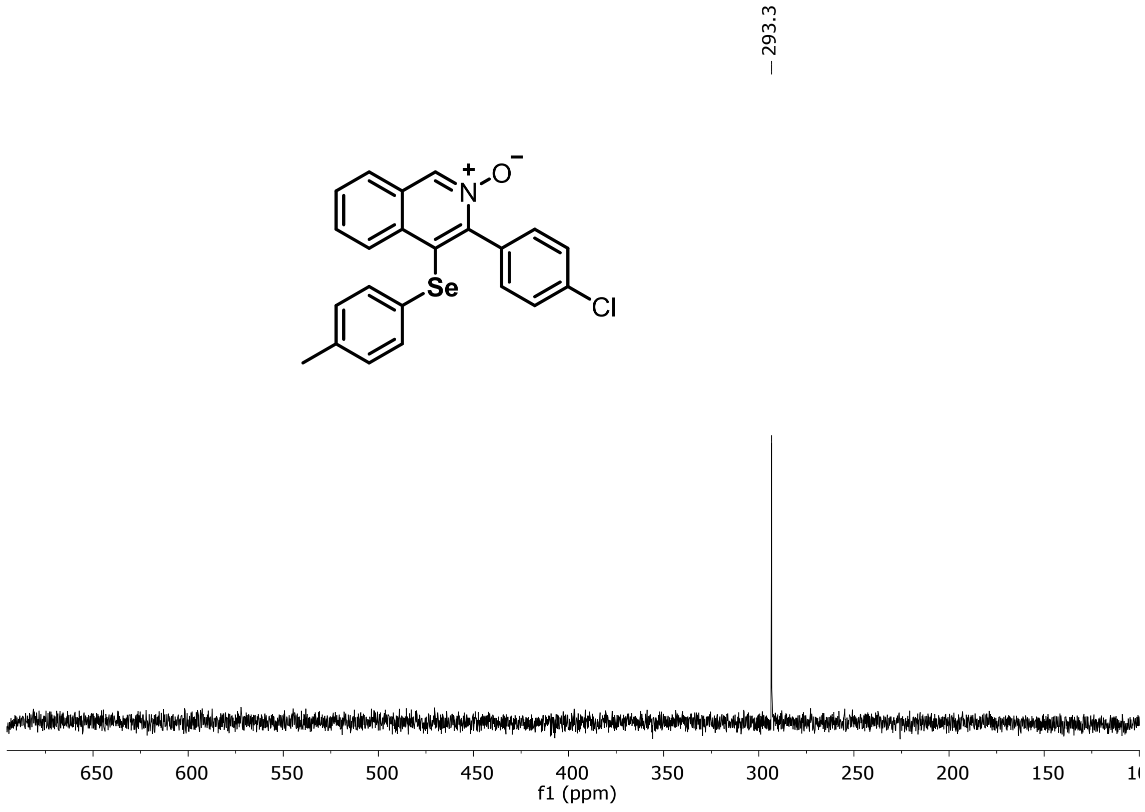

Figure S30. $^{77}Se\{^{1}H\}$ NMR (76 MHz, $CDCl_3$) spectrum of the compound **3j**.

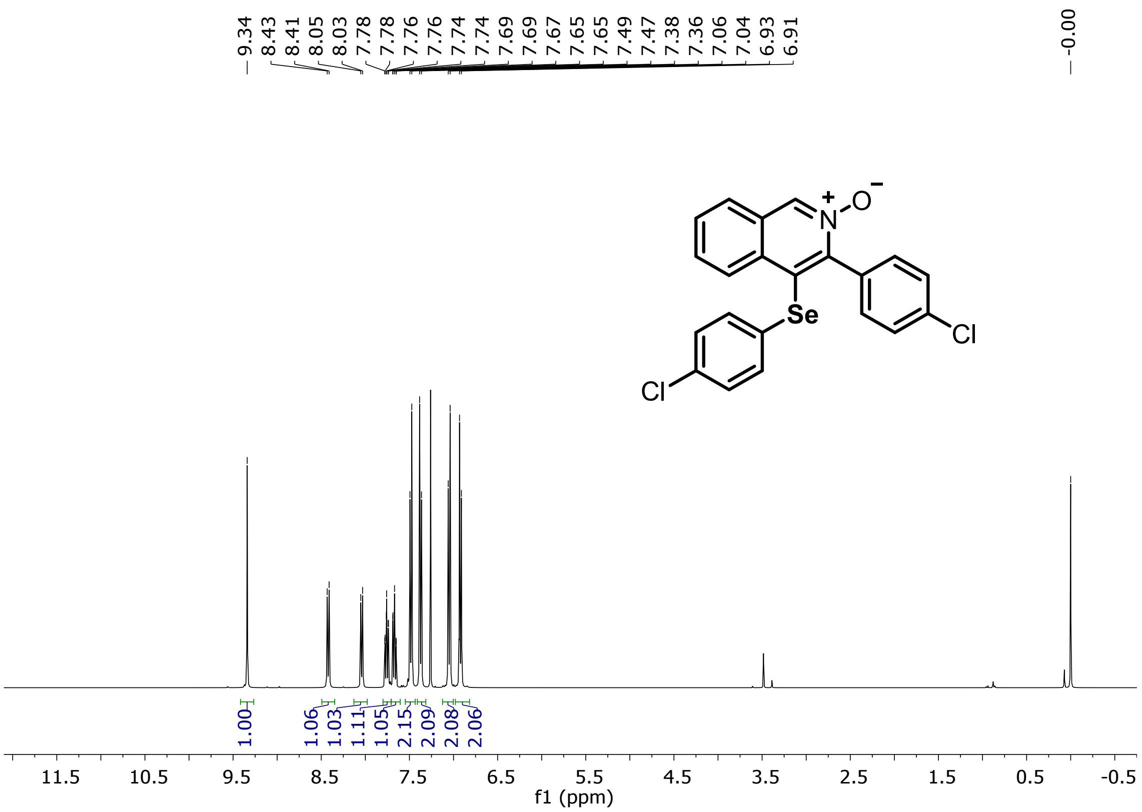

Figure S31. ^{1}H NMR (400 MHz, $CDCl_3$) spectrum of the compound **3k**.

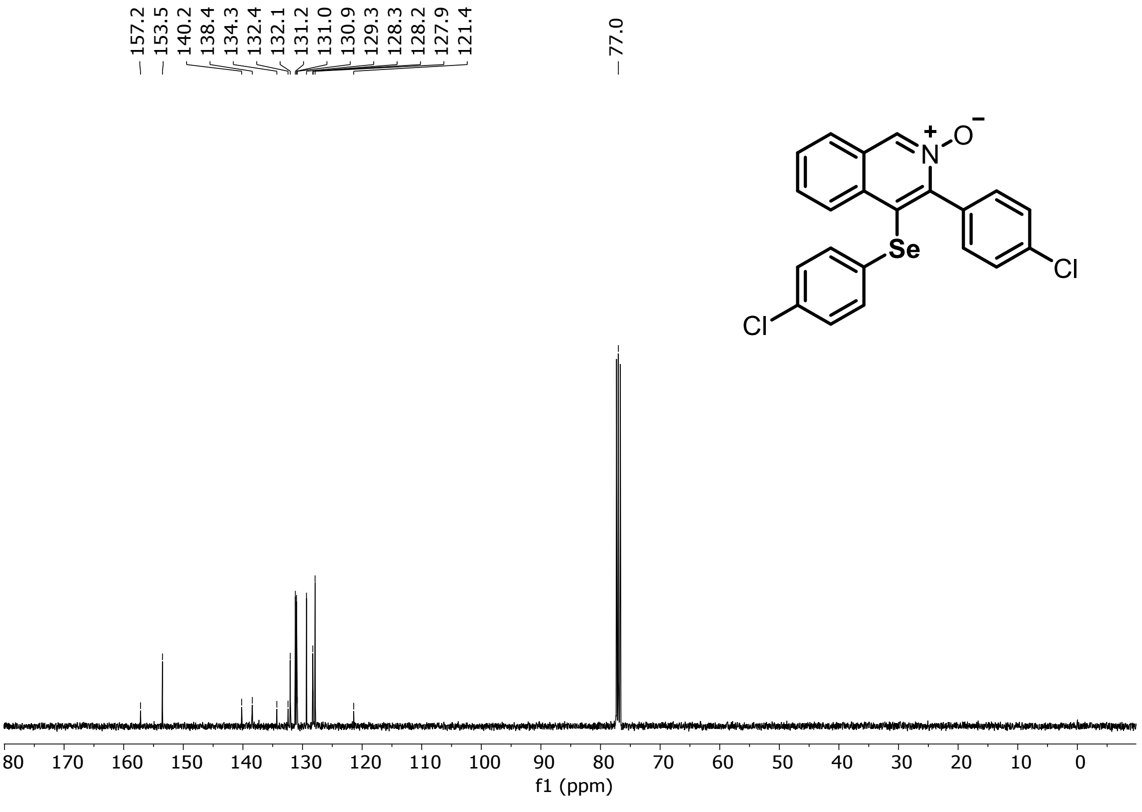

Figure S32. $^{13}C\{^{1}H\}$ NMR (100 MHz, $CDCl_3$) spectrum of the compound **3k**.

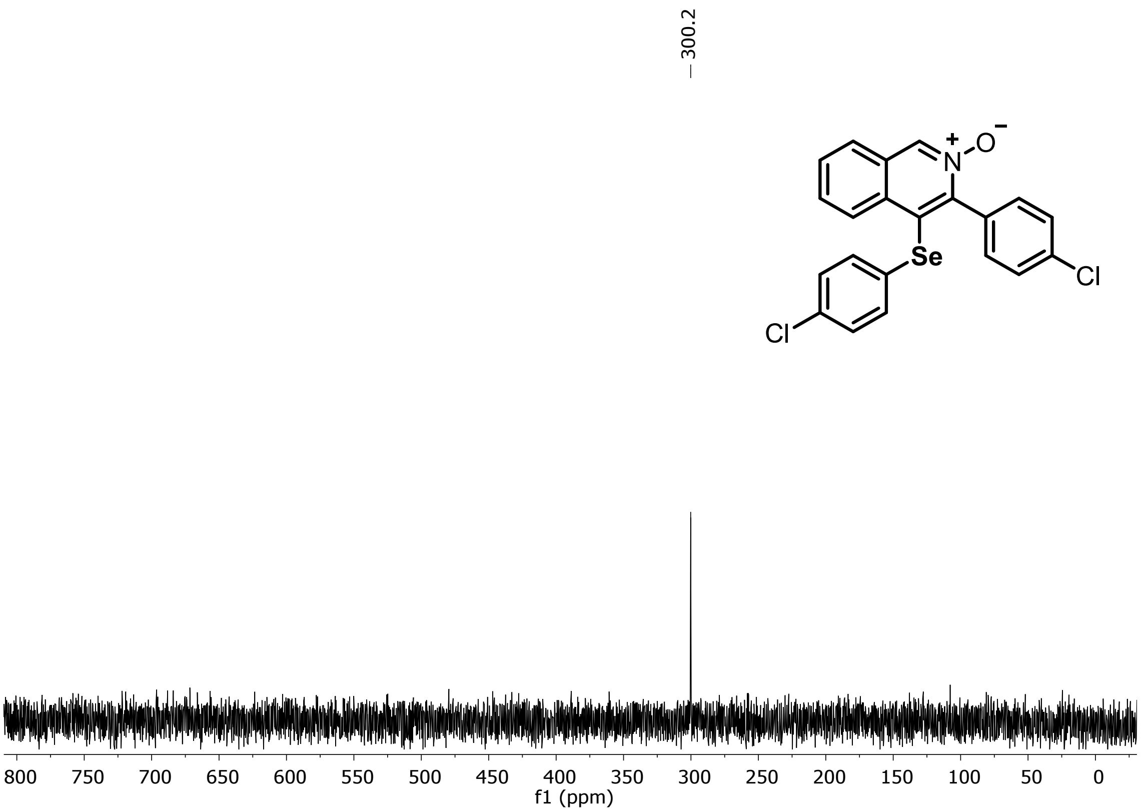

Figure S33. ^{77}Se{^{1}H} NMR (76 MHz, $CDCl_3$) spectrum of the compound **3k**.

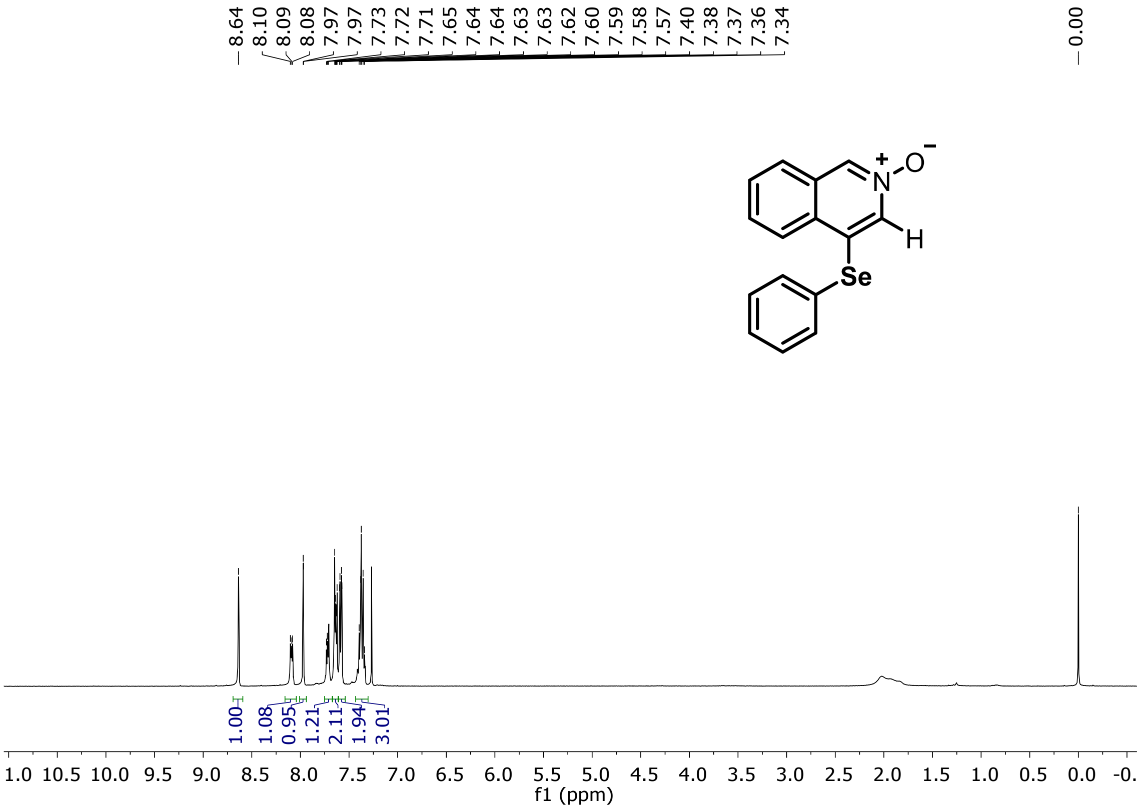

Figure S34. ^{1}H NMR (400 MHz, $CDCl_3$) spectrum of the compound **3l**.

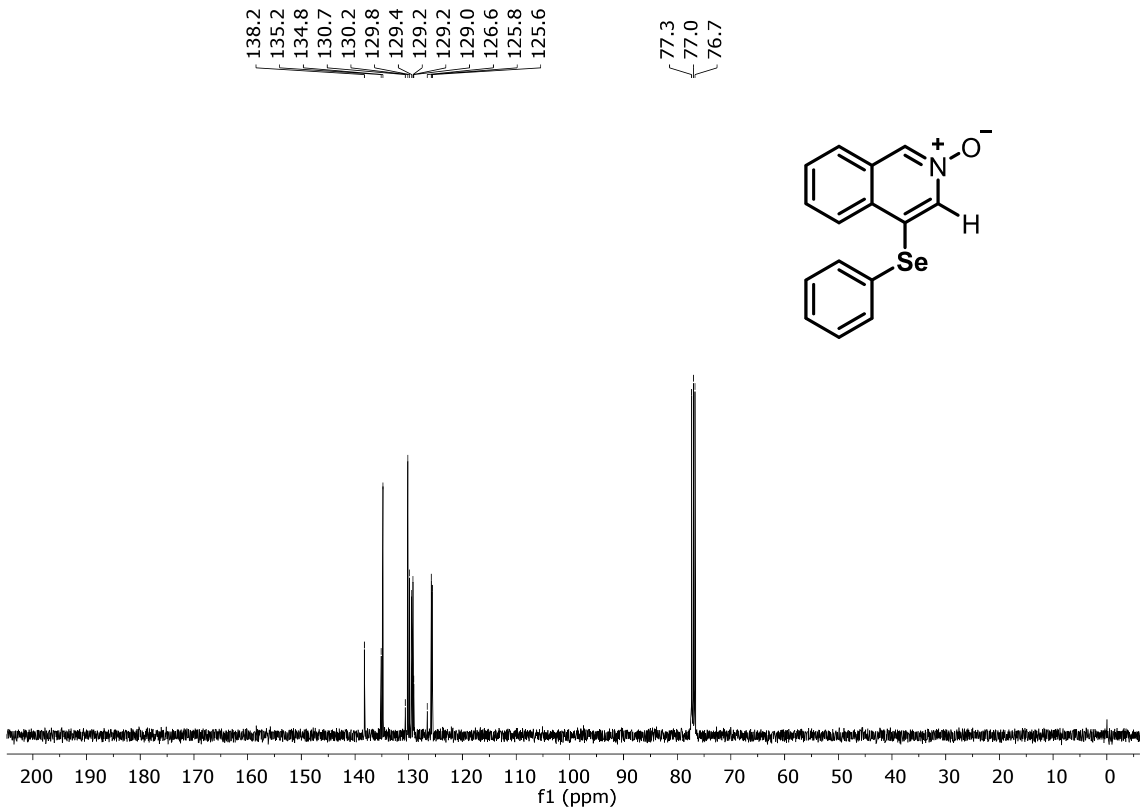

Figure S35. $^{13}C\{^1H\}$ NMR (100 MHz, $CDCl_3$) spectrum of the compound **3l**.

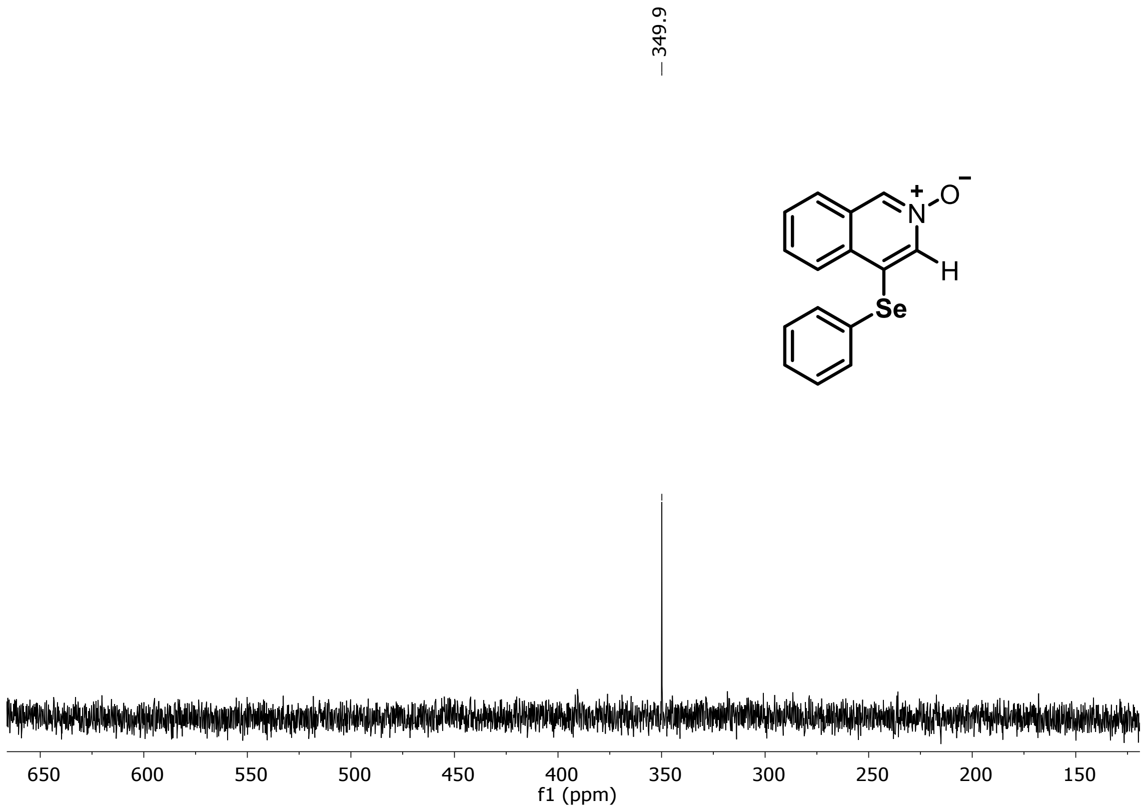

Figure S36. $^{77}Se\{^1H\}$ NMR (76 MHz, $CDCl_3$) spectrum of the compound **3l**.

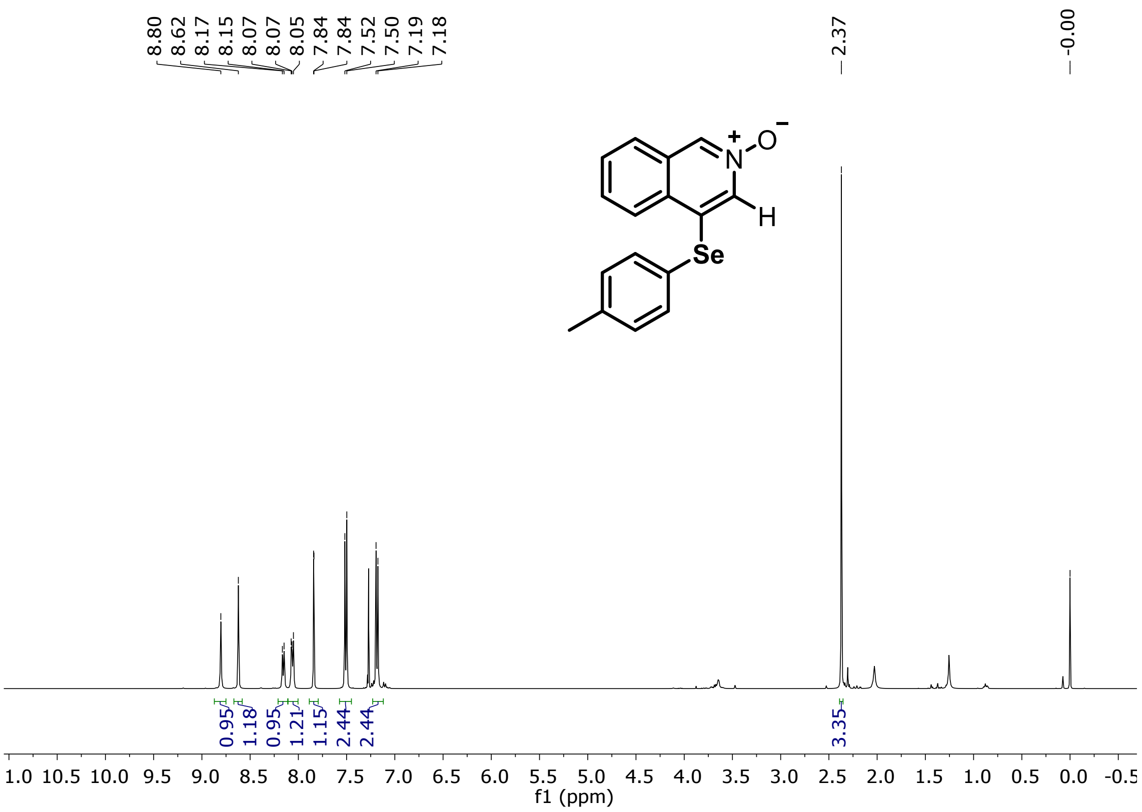

Figure S37. ^{1}H NMR (400 MHz, $CDCl_3$) spectrum of the compound **3m**.

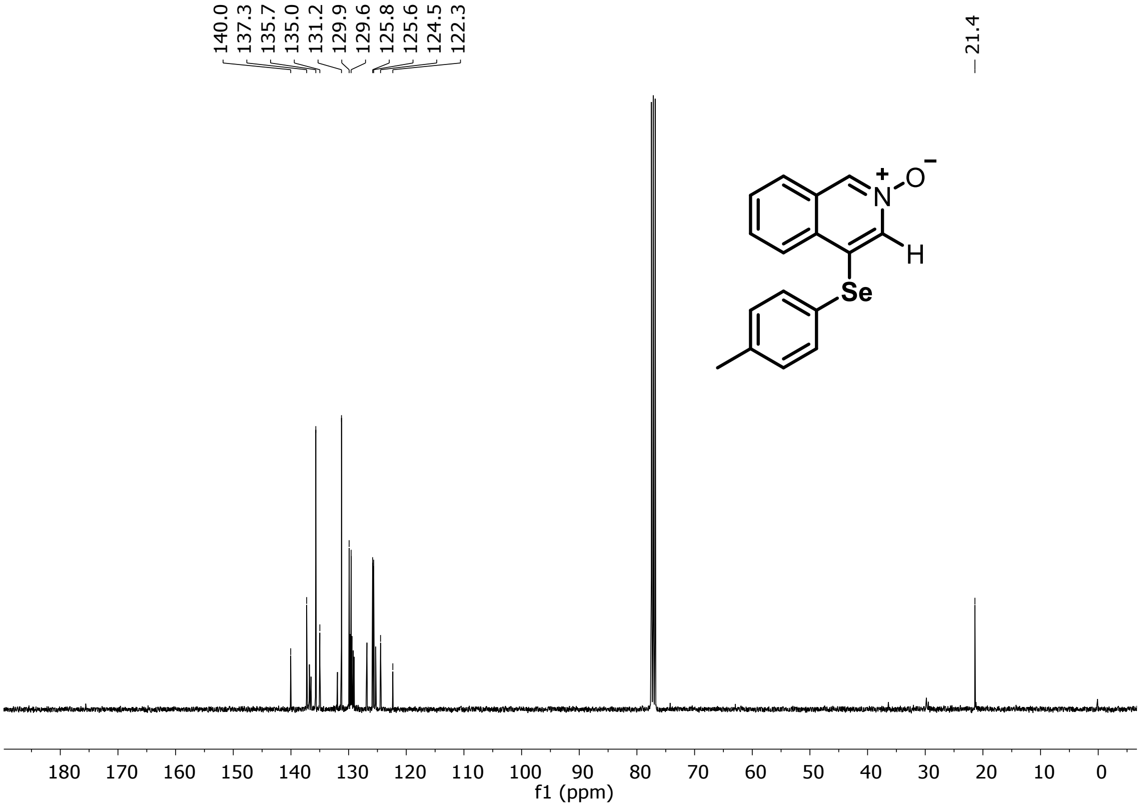

Figure S38. $^{13}C\{^{1}H\}$ NMR (100 MHz, $CDCl_3$) spectrum of the compound **3m**.

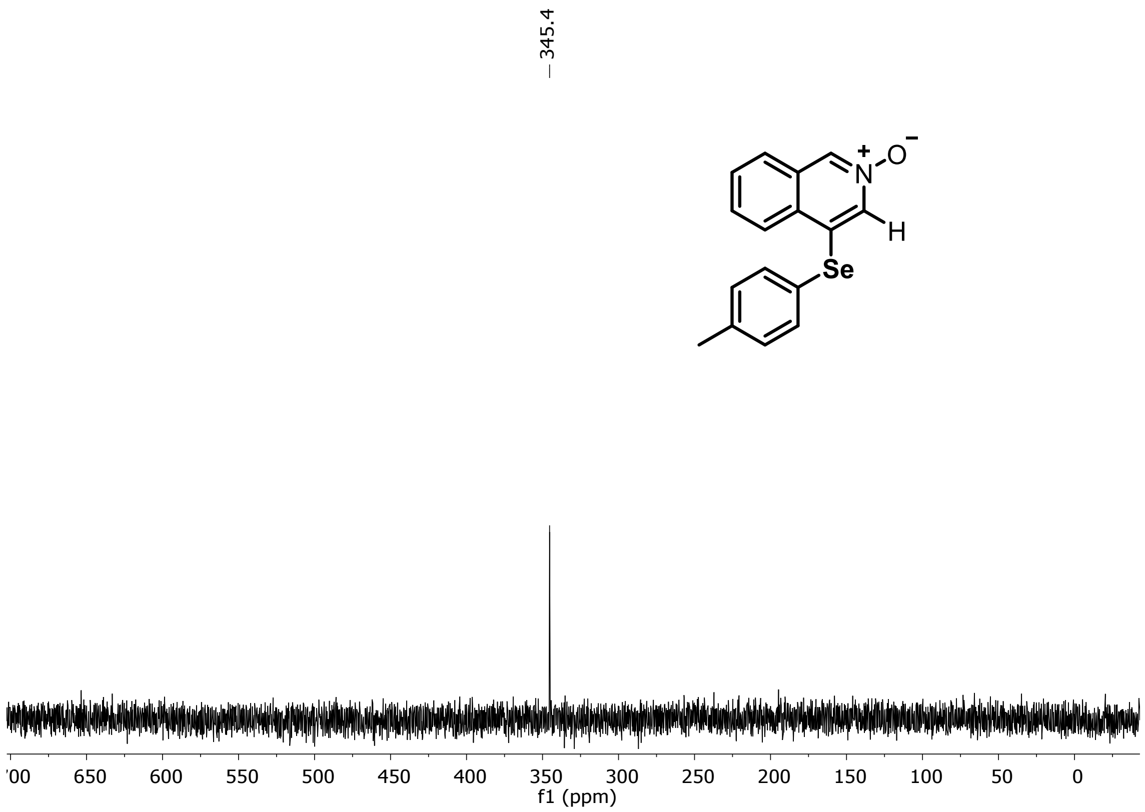

Figure S39. ^{77}Se{^{1}H} NMR (76 MHz, $CDCl_3$) spectrum of the compound **3m**.

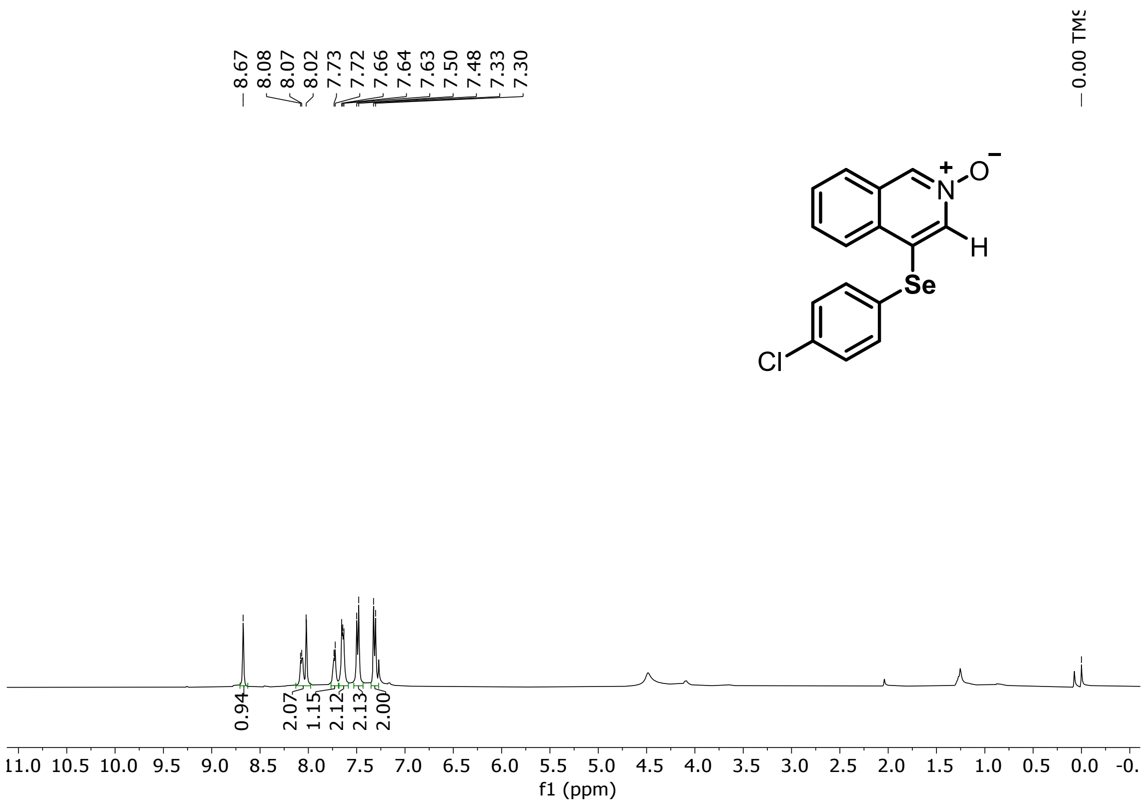

Figure S40. ^{1}H NMR (400 MHz, $CDCl_3$) spectrum of the compound **3n**.

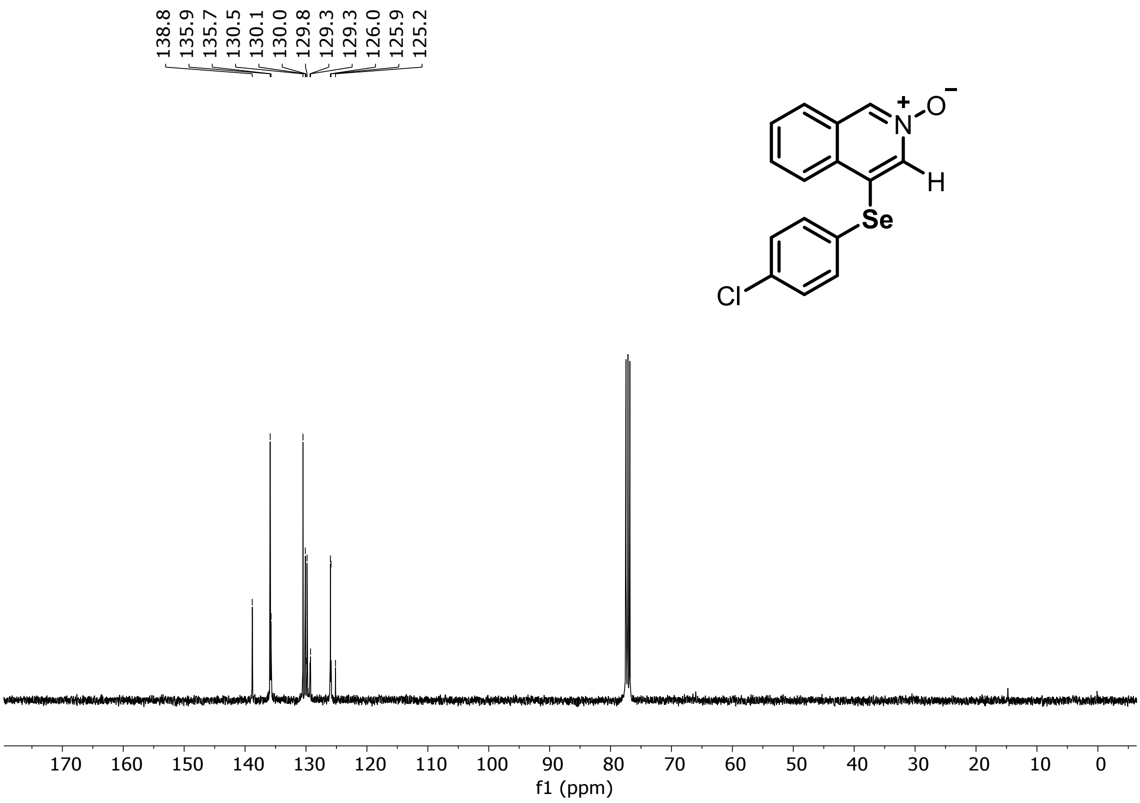

Figure S41. $^{13}C\{^1H\}$ NMR (100 MHz, $CDCl_3$) spectrum of the compound **3n**.

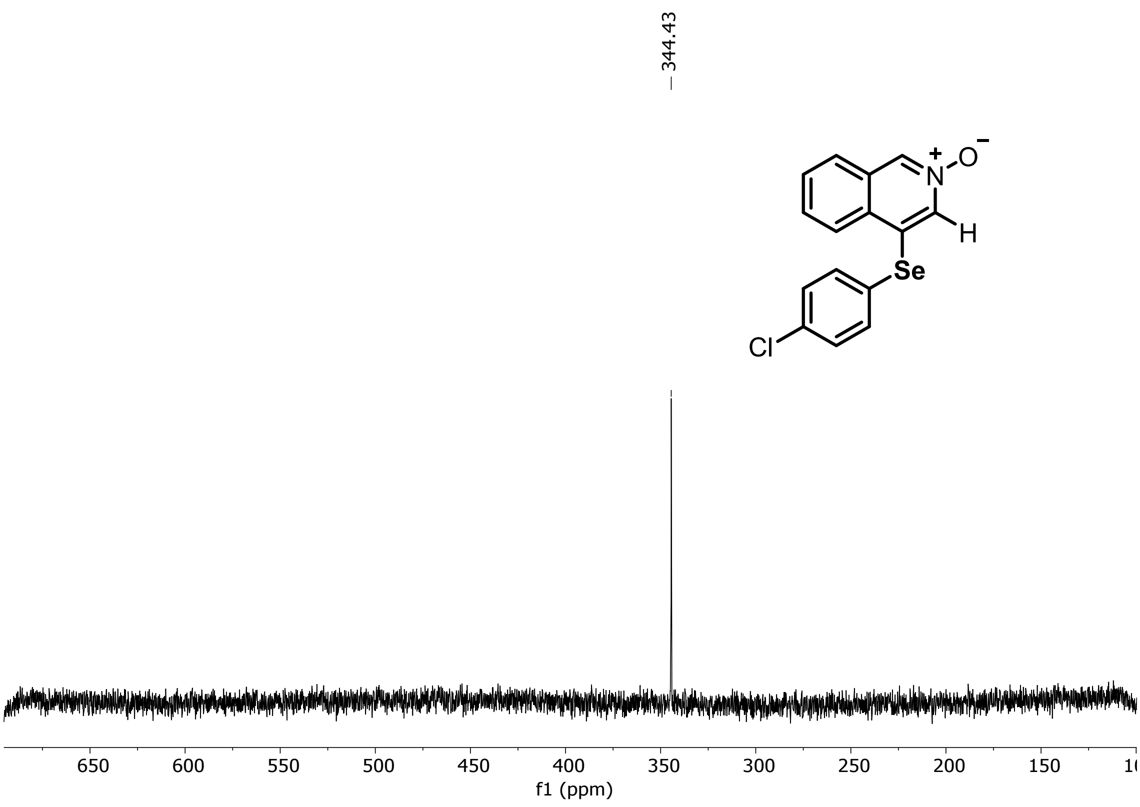

Figure S42. $^{77}Se\{^1H\}$ NMR (76 MHz, $CDCl_3$) spectrum of the compound **3n**.

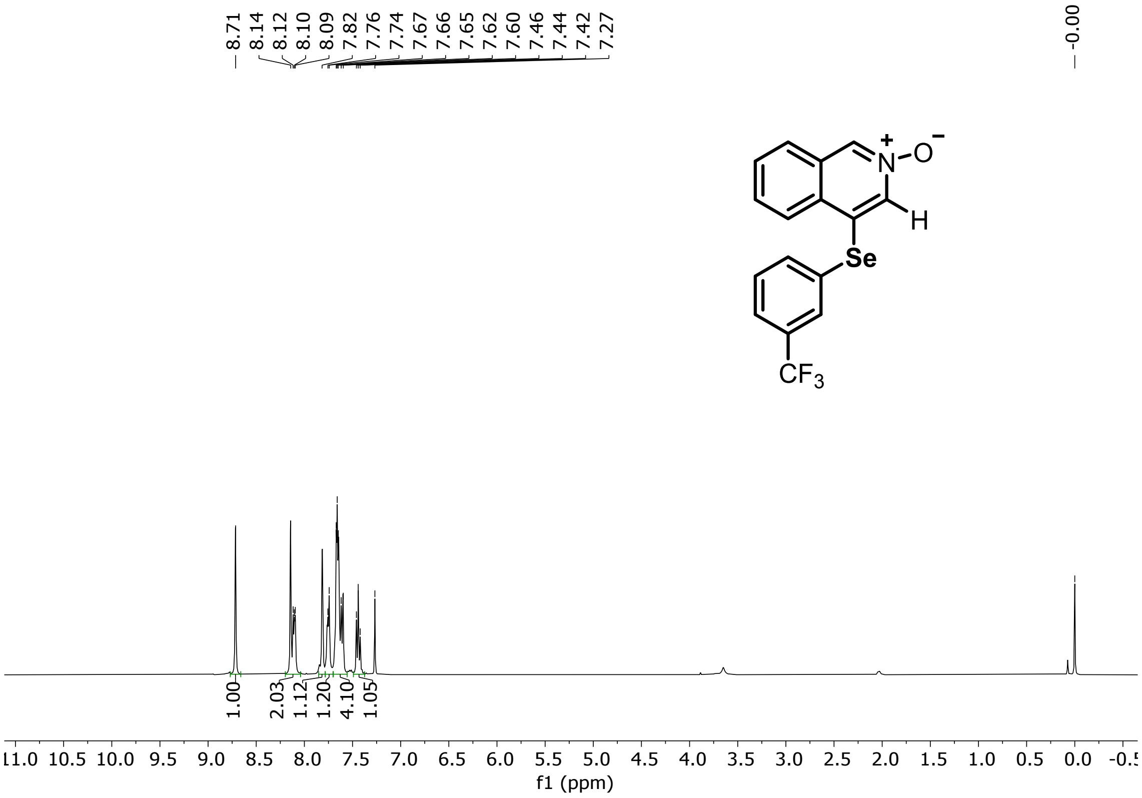

Figure S43. ^{1}H NMR (400 MHz, $CDCl_3$) spectrum of the compound **3o**.

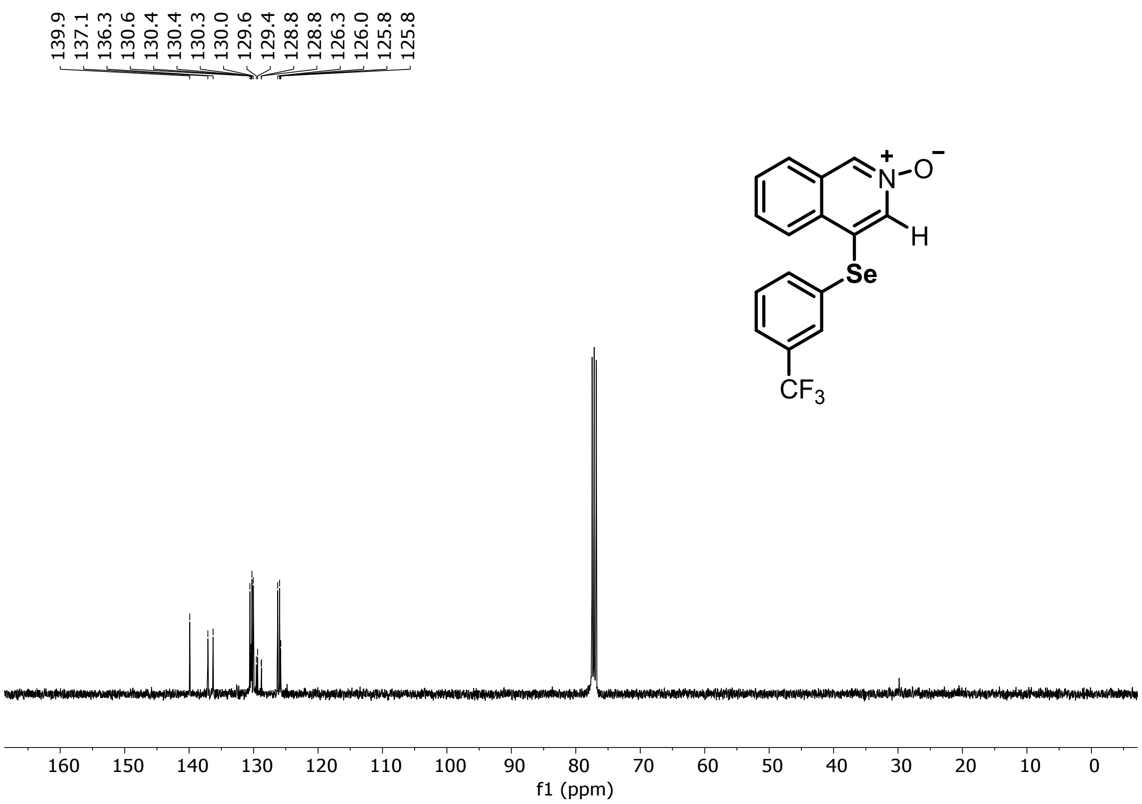

Figure S44. $^{13}C\{^{1}H\}$ NMR (100 MHz, $CDCl_3$) spectrum of the compound **3o**.

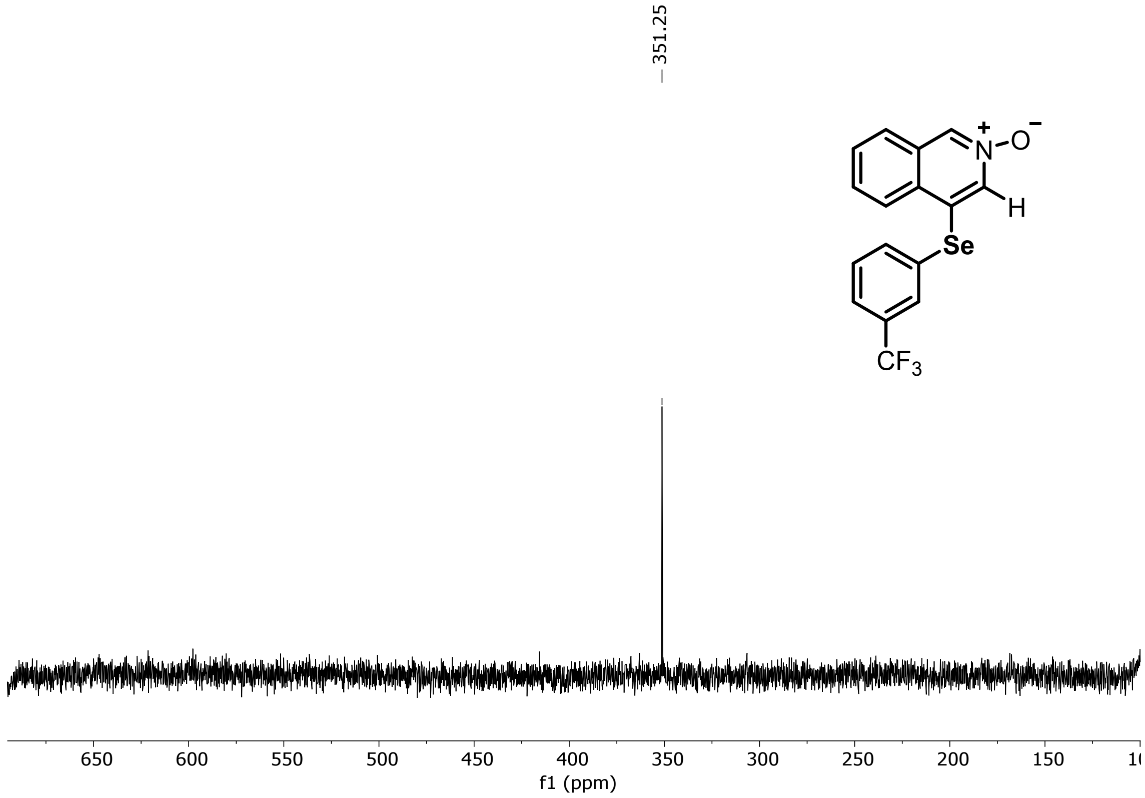

Figure S45. $^{77}Se\{^{1}H\}$ NMR (76 MHz, $CDCl_3$) spectrum of the compound **3o**.

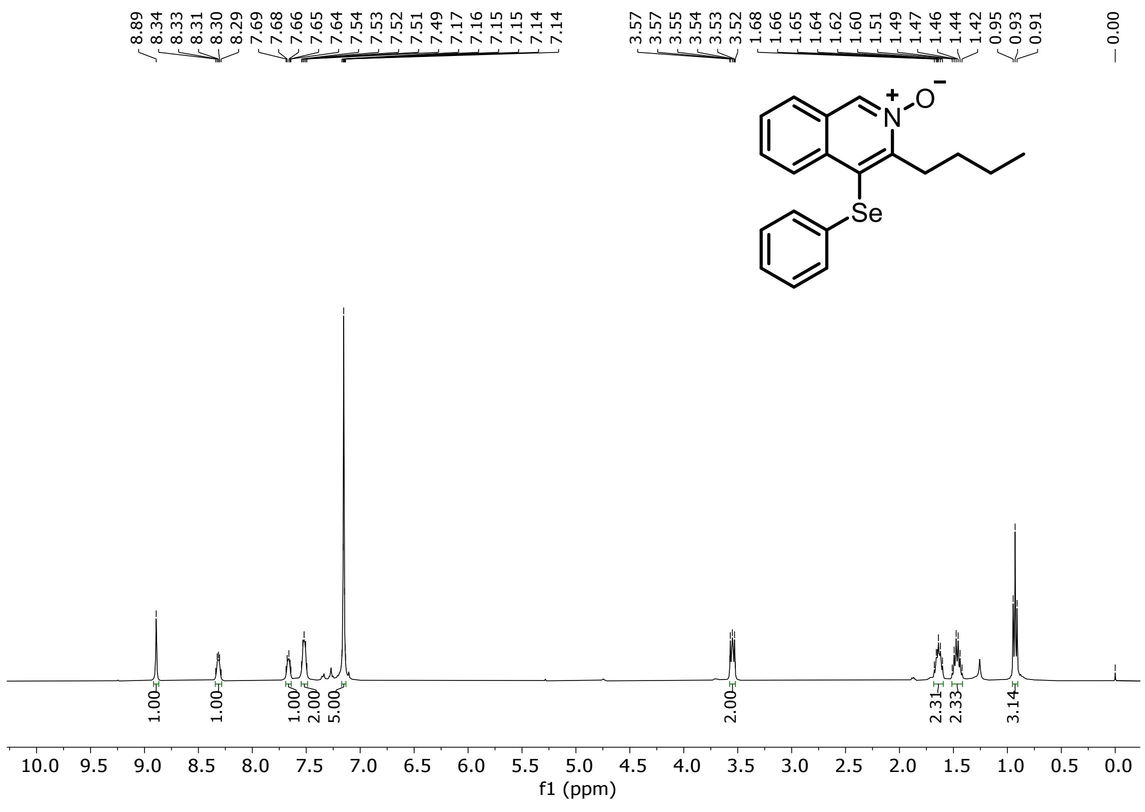

Figure S46. ^{1}H NMR (400 MHz, $CDCl_3$) spectrum of the compound **3p**.

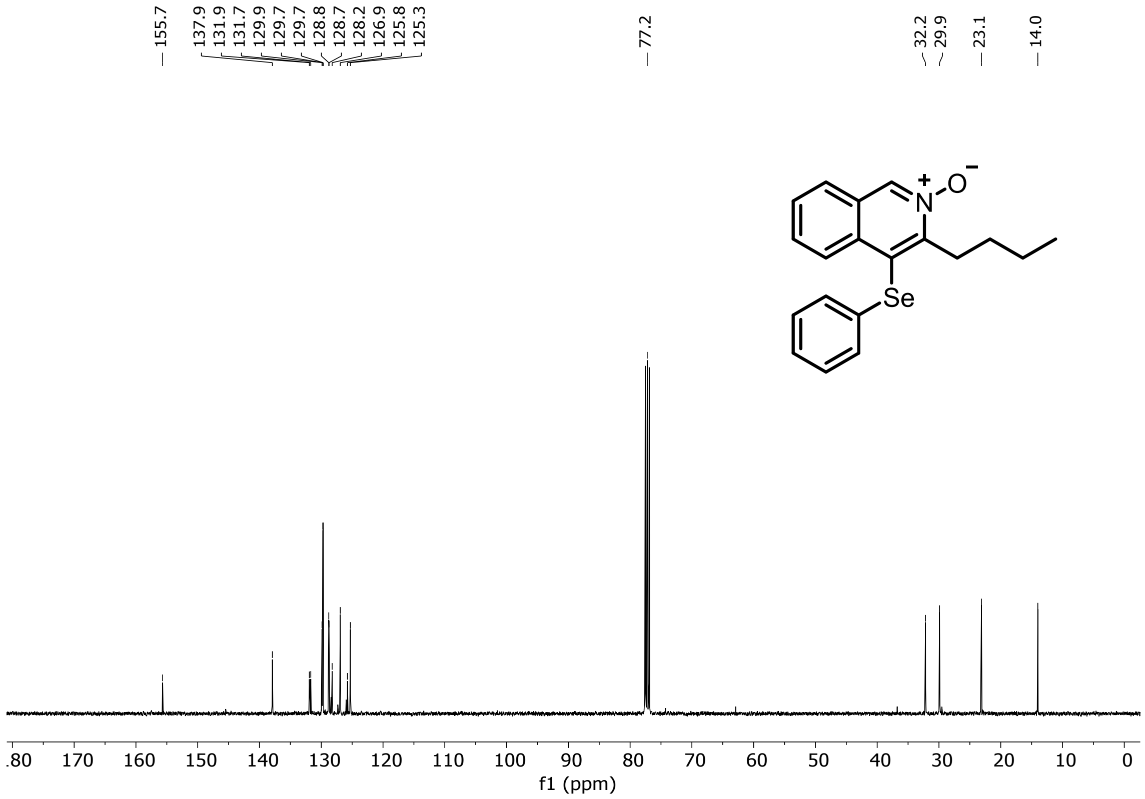

Figure S47. $^{13}C\{^1H\}$ NMR (100 MHz, $CDCl_3$) spectrum of the compound **3p**.

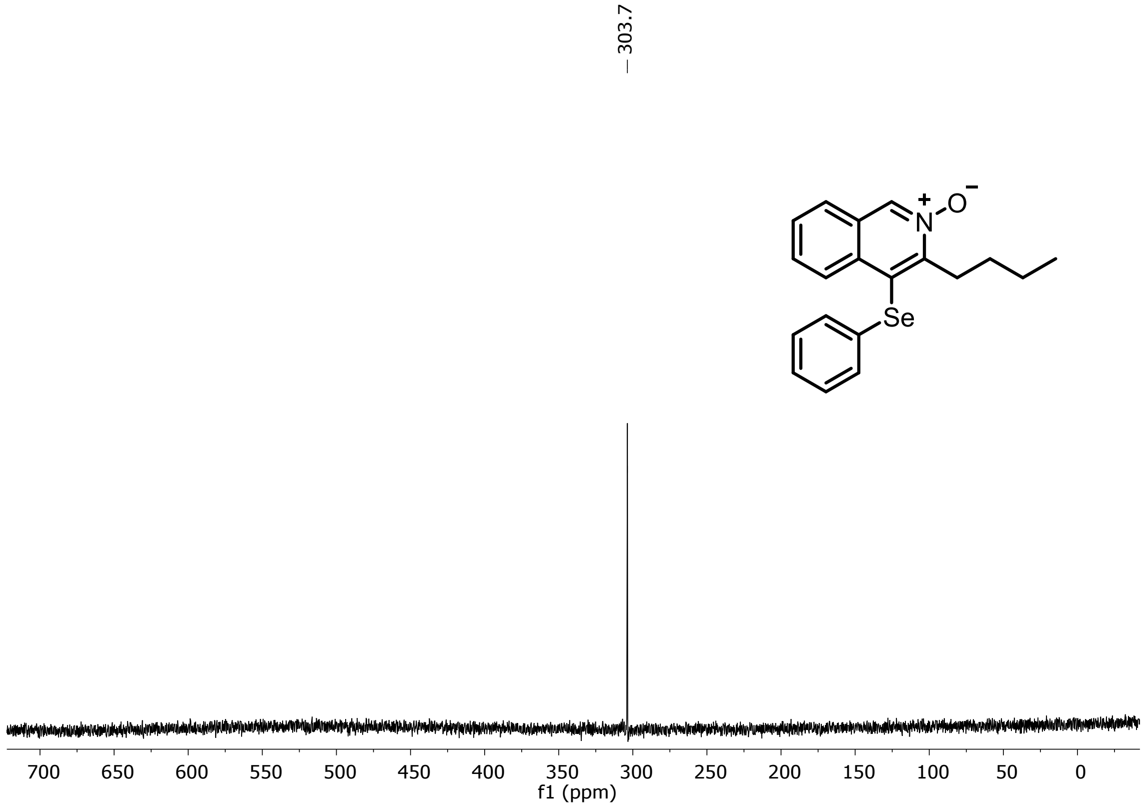

Figure S48. $^{77}Se\{^1H\}$ NMR (76 MHz, $CDCl_3$) spectrum of the compound **3p**.

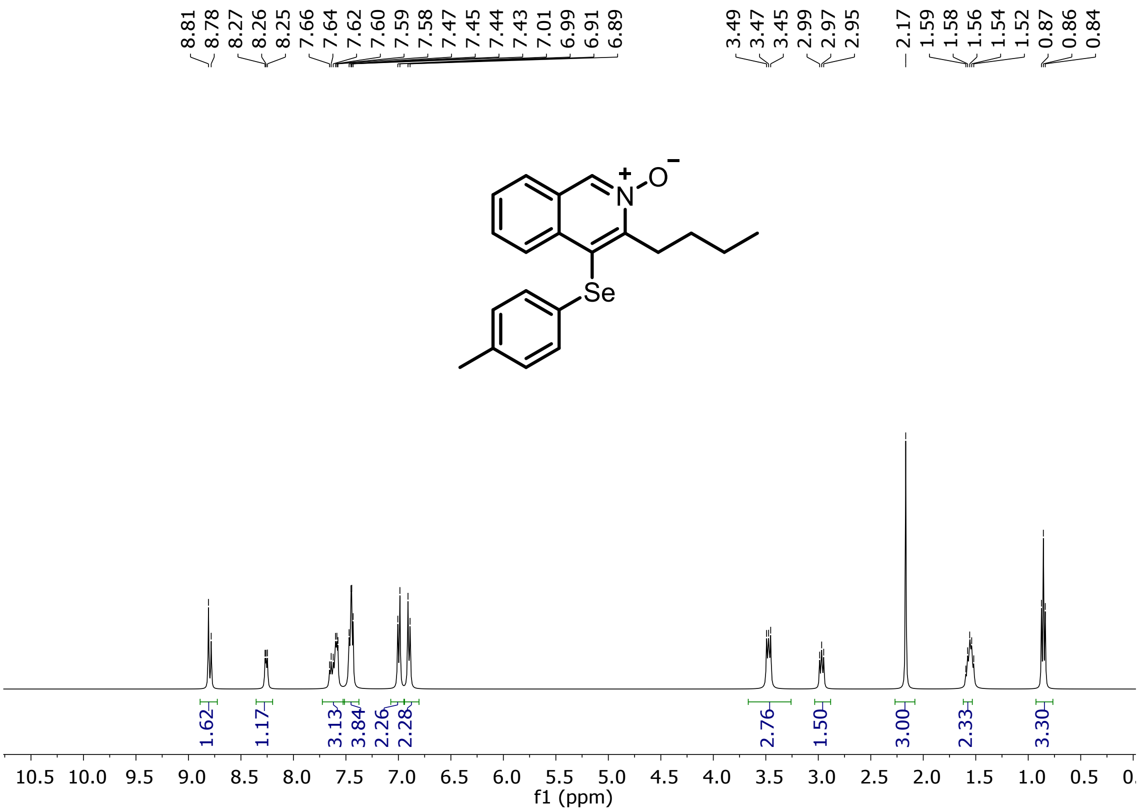

Figure S49. ^{1}H NMR (400 MHz, $CDCl_3$) spectrum of the compound **3q**.

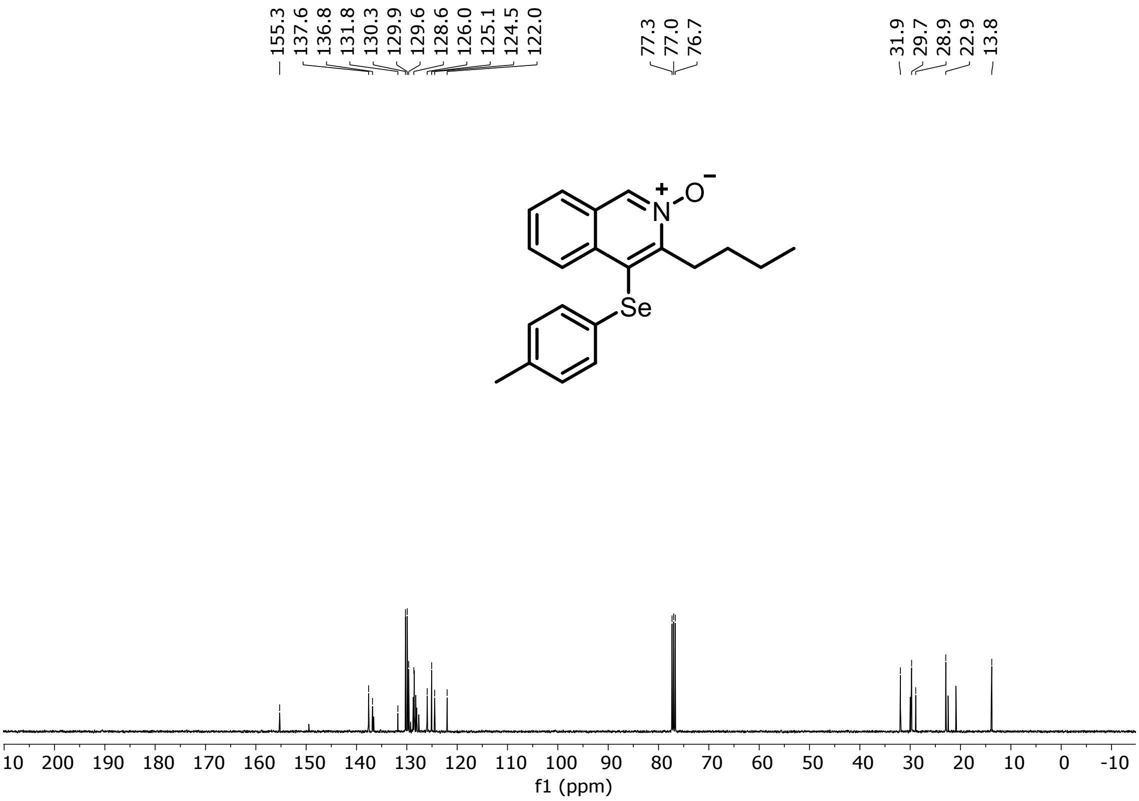

Figure S50. $^{13}C\{^{1}H\}$ NMR (100 MHz, $CDCl_3$) spectrum of the compound **3q**.

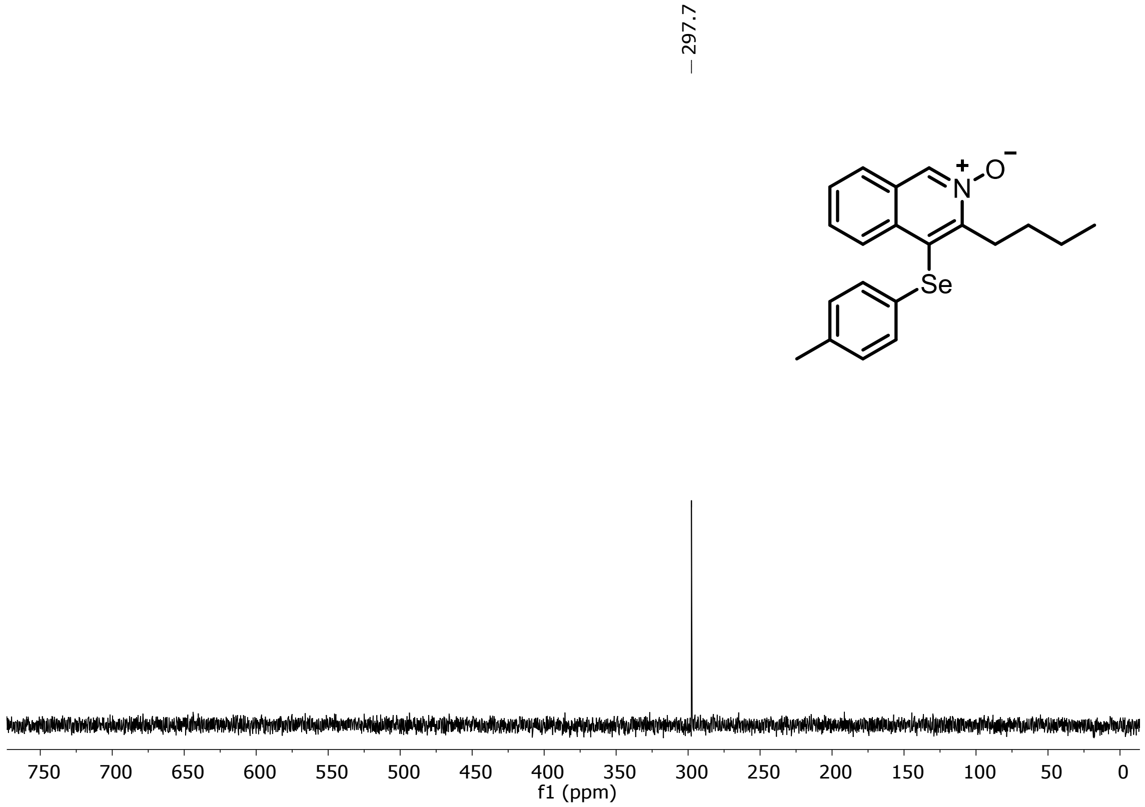

Figure S51. $^{77}Se\{^{1}H\}$ NMR (76 MHz, $CDCl_3$) spectrum of the compound **3q**.

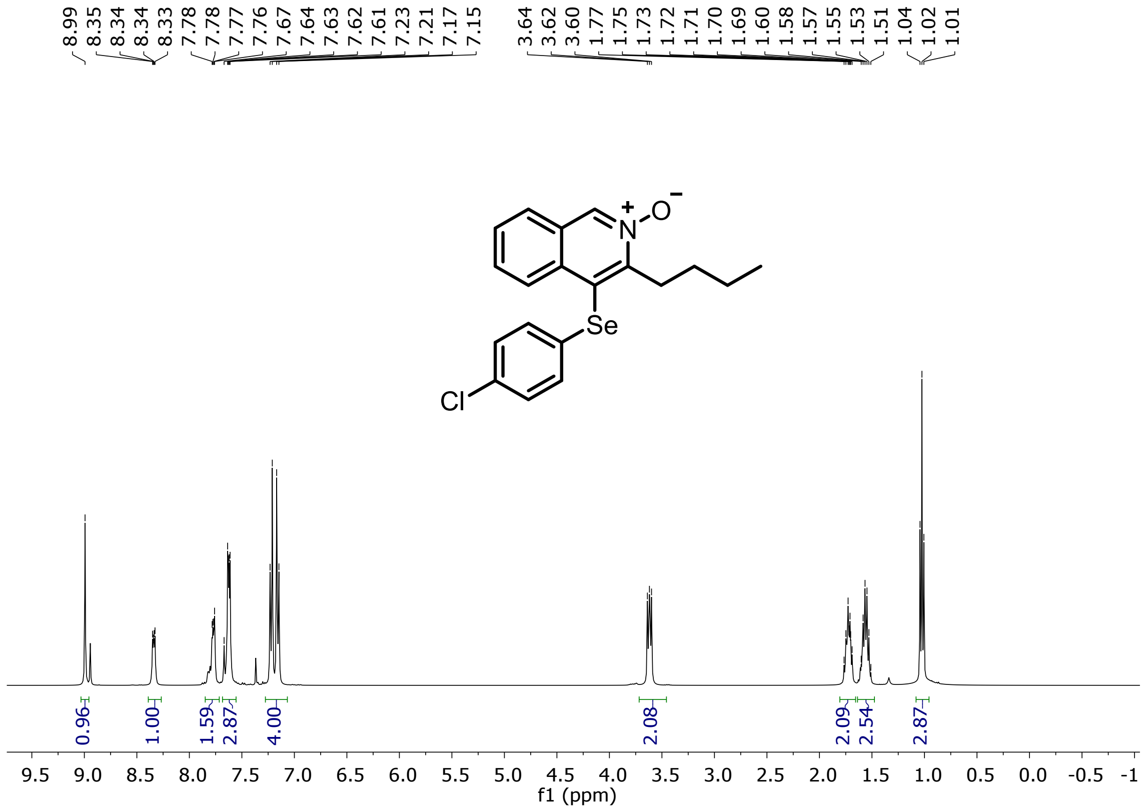

Figure S52. ^{1}H NMR (400 MHz, $CDCl_3$) spectrum of the compound **3r**.

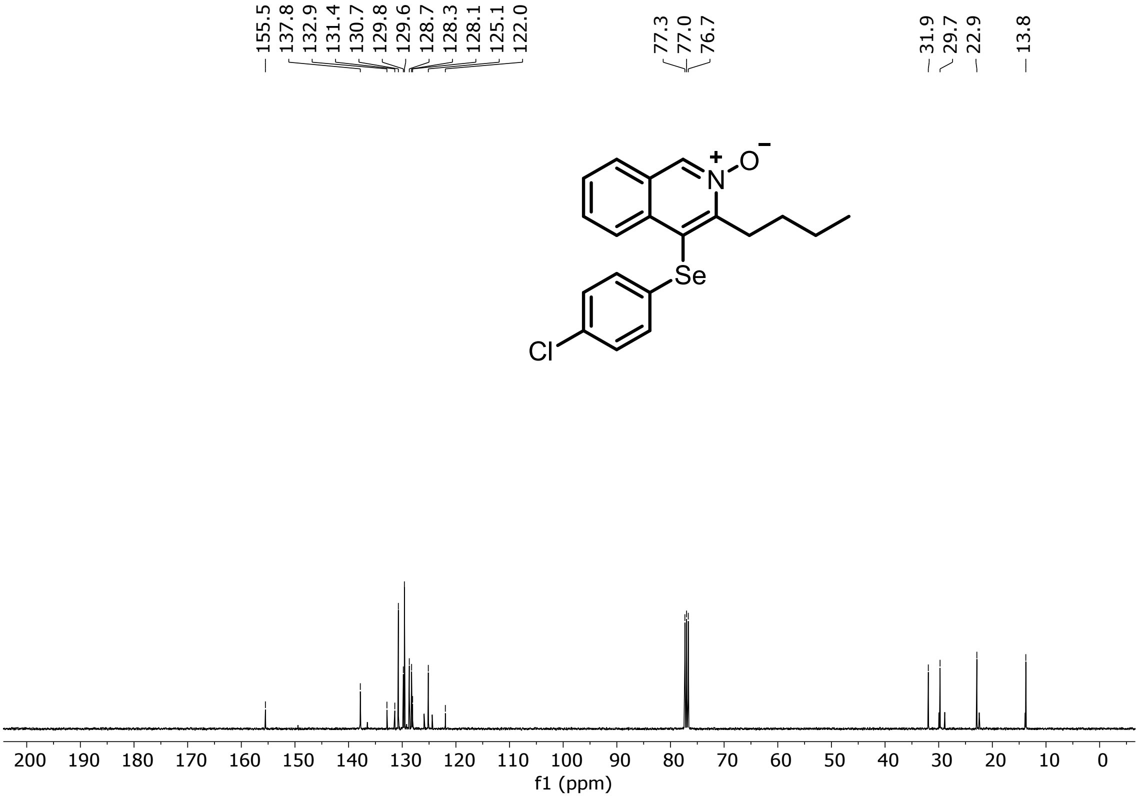

Figure S53. $^{13}C\{^{1}H\}$ NMR (100 MHz, $CDCl_3$) spectrum of the compound **3r**.

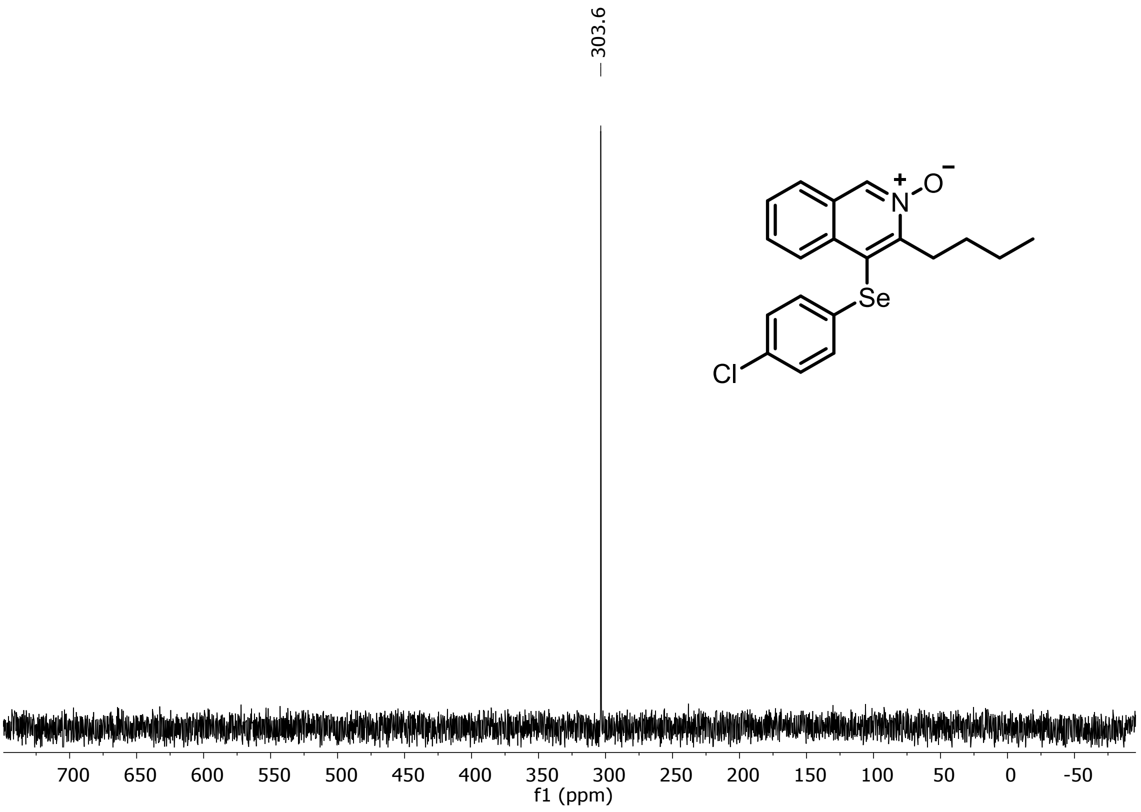

Figure S54. $^{77}Se\{^{1}H\}$ NMR (76 MHz, $CDCl_3$) spectrum of the compound **3r**.

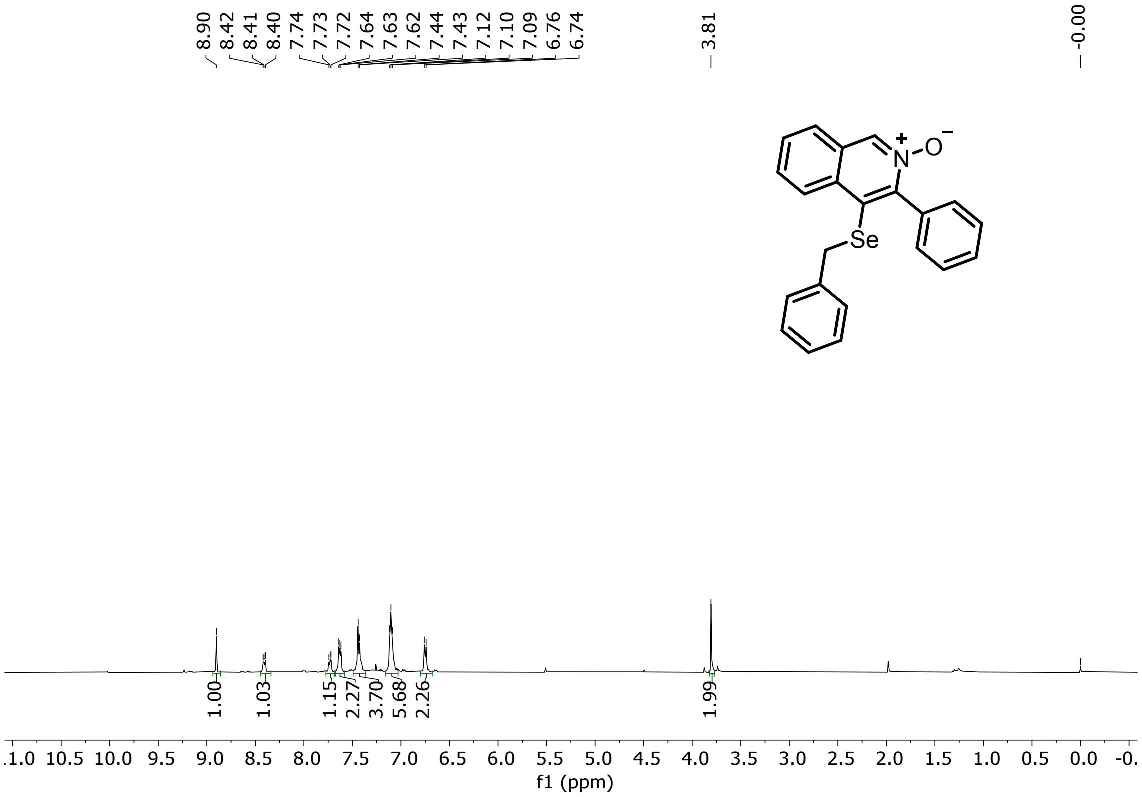

Figure S55. ^{1}H NMR (400 MHz, $CDCl_3$) spectrum of the compound **3s**.

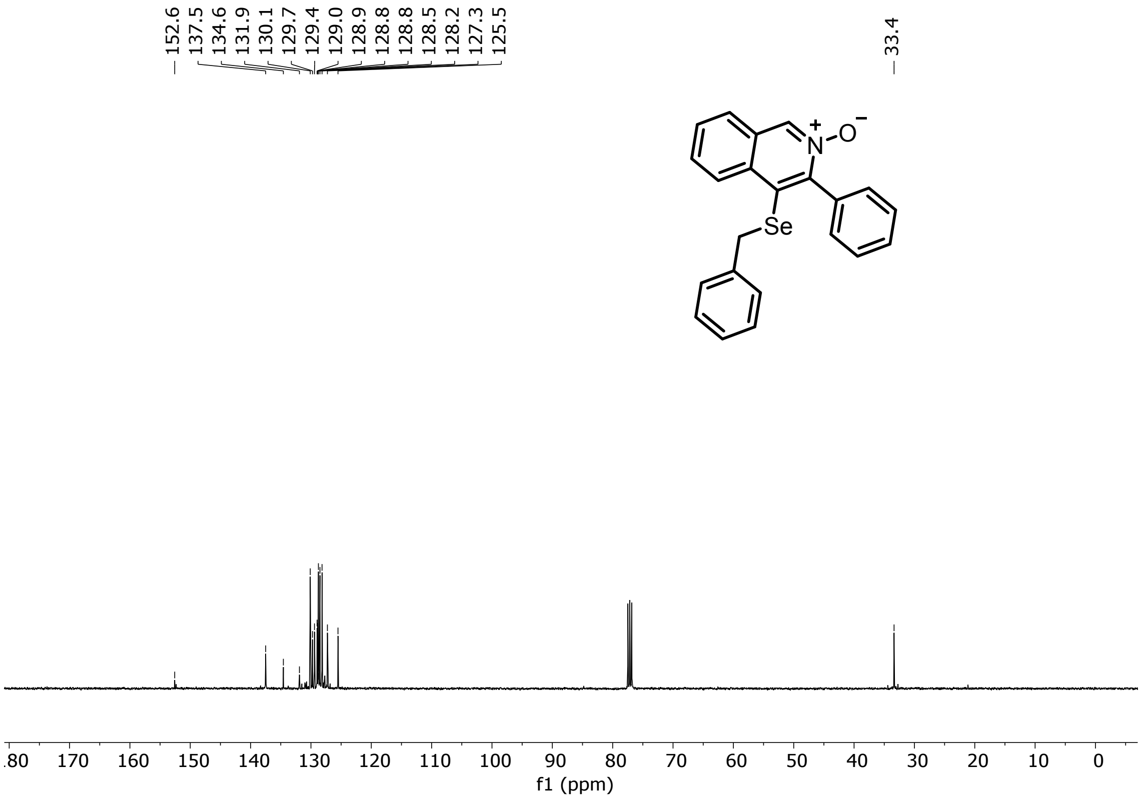

Figure S56. $^{13}C\{^{1}H\}$ NMR (100 MHz, $CDCl_3$) spectrum of the compound **3s**.

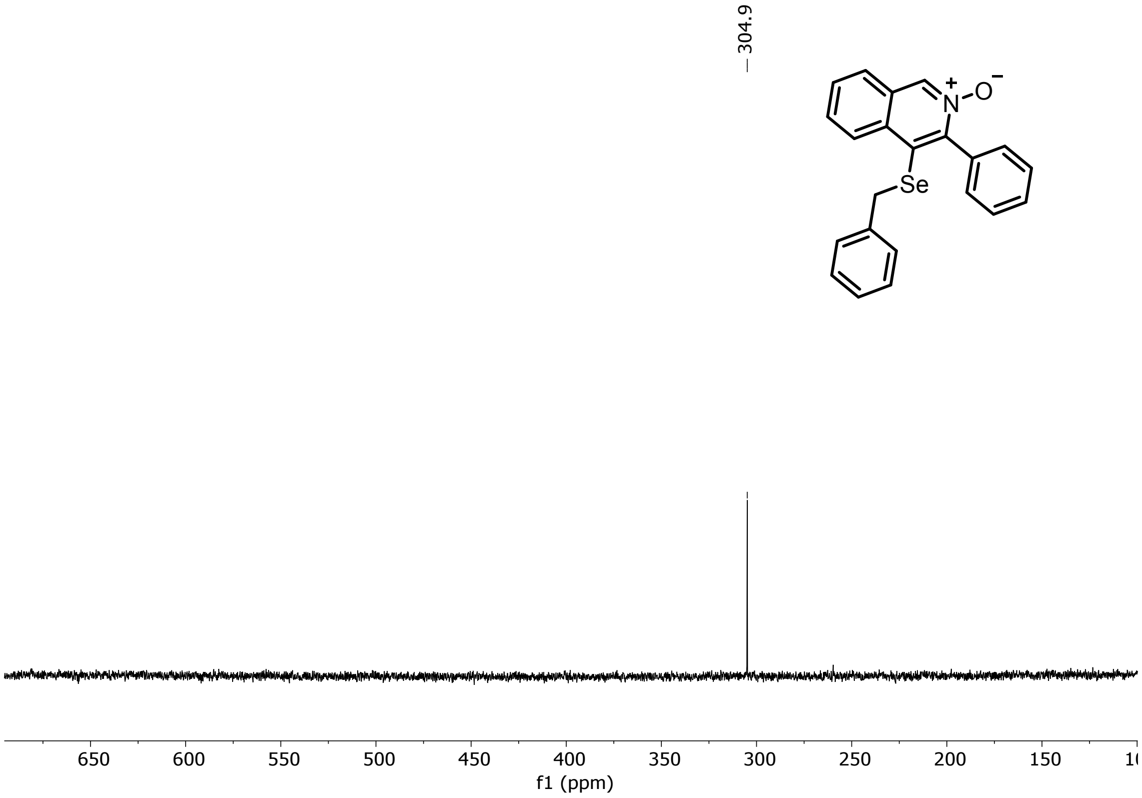

Figure S57. $^{77}Se\{^1H\}$ NMR (76 MHz, $CDCl_3$) spectrum of the compound **3s**.

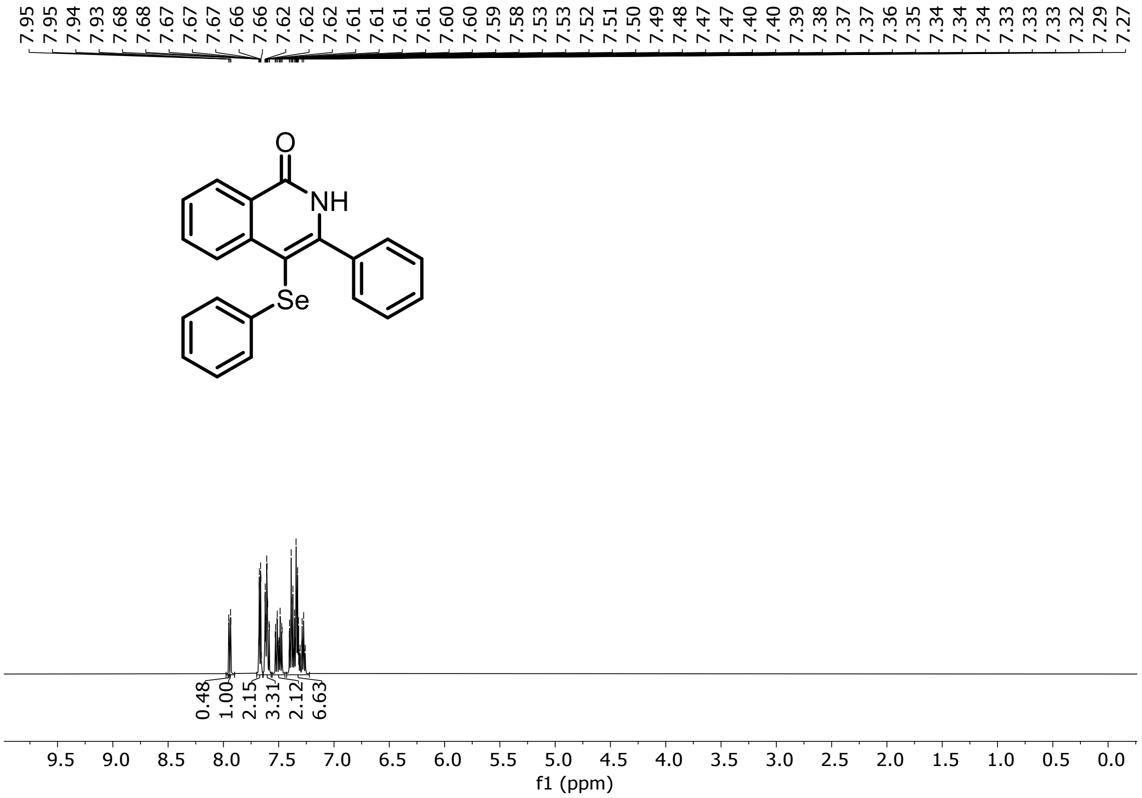

Figure S58. ^1H NMR (400 MHz, $CDCl_3$) spectrum of the compound **4**.

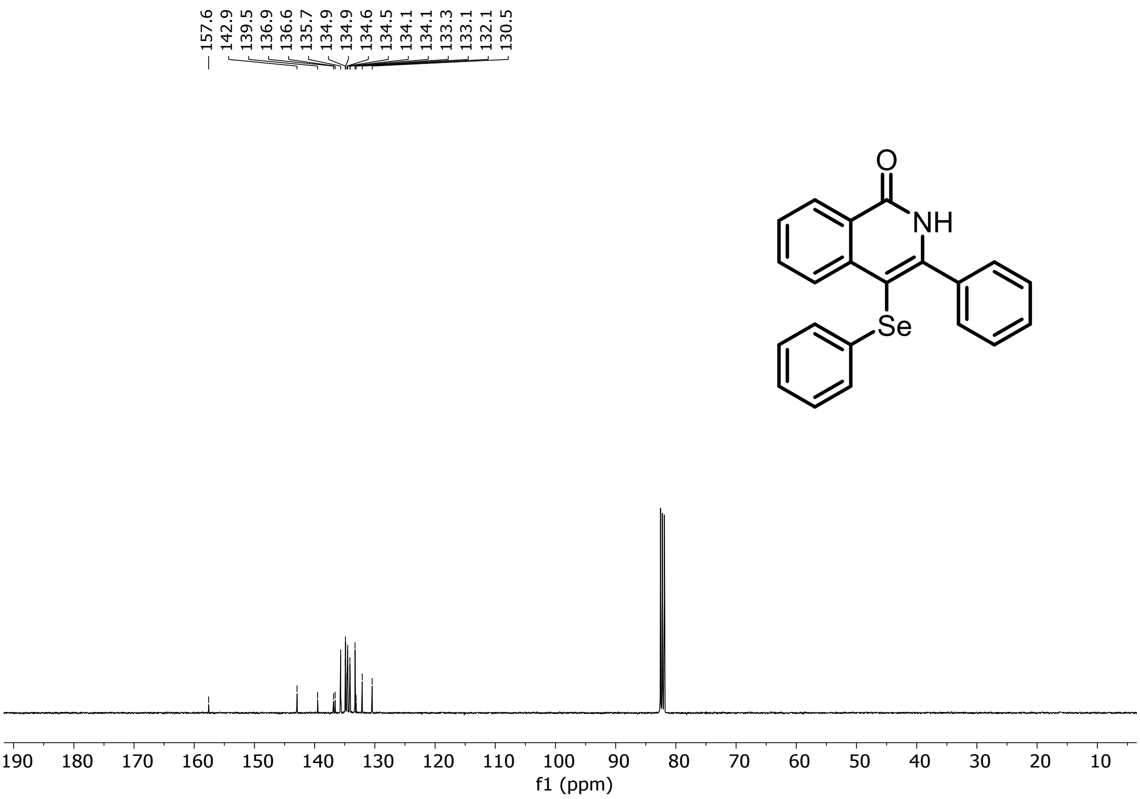

Figure S59. $^{13}C\{^{1}H\}$ NMR (100 MHz, $CDCl_3$) spectrum of the compound **4**.

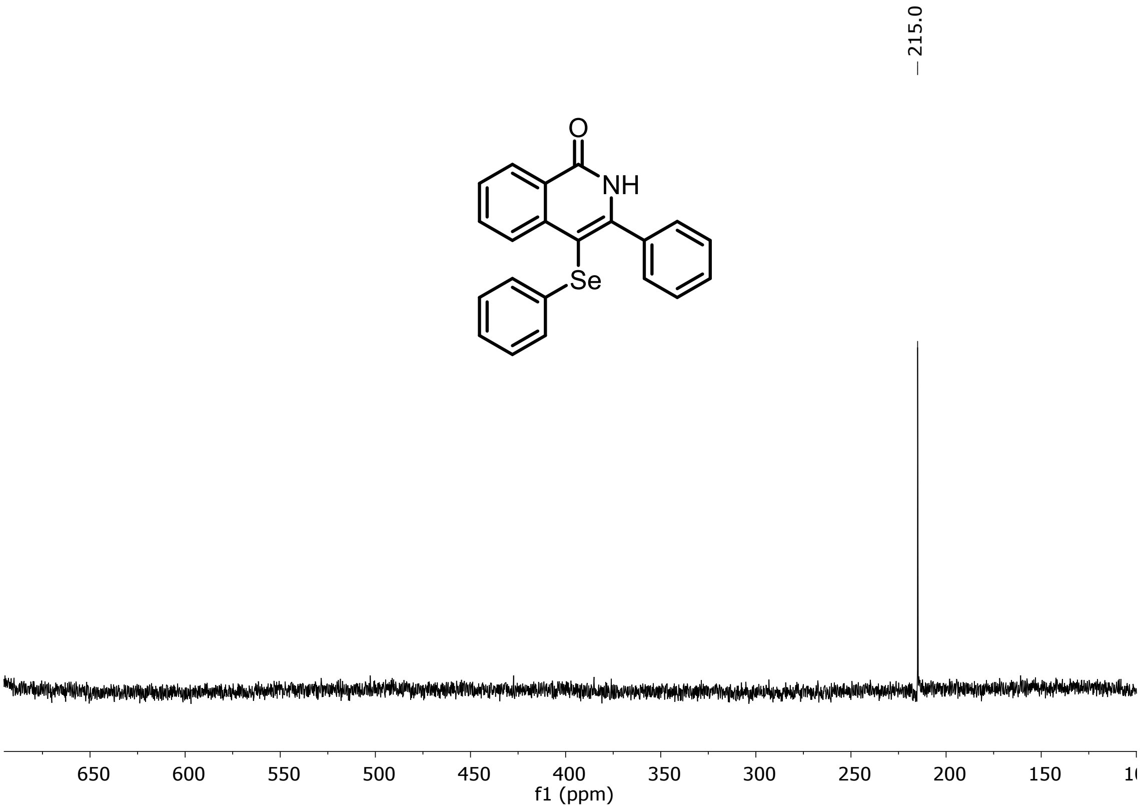

Figure S60. $^{77}Se\{^{1}H\}$ NMR (76 MHz, $CDCl_3$) spectrum of the compound **4.**

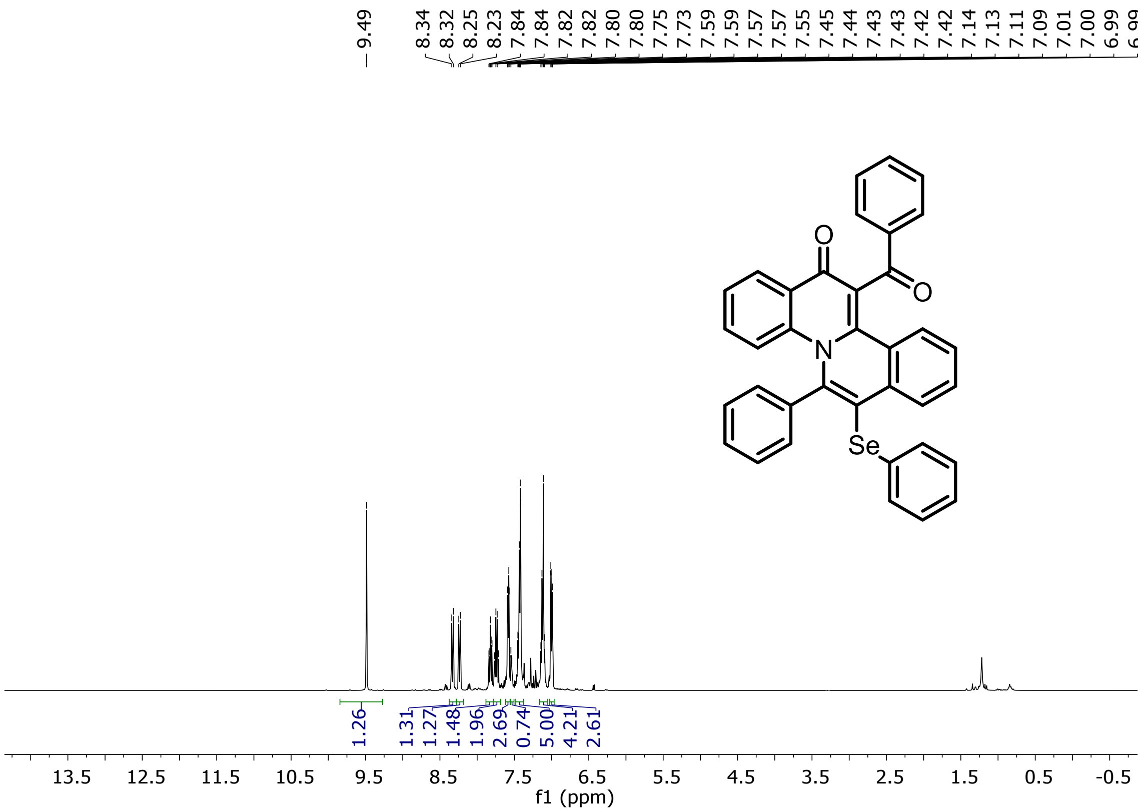

Figure S61. ^{1}H NMR (400 MHz, DMSO-d_6) spectrum of the compound **6**.

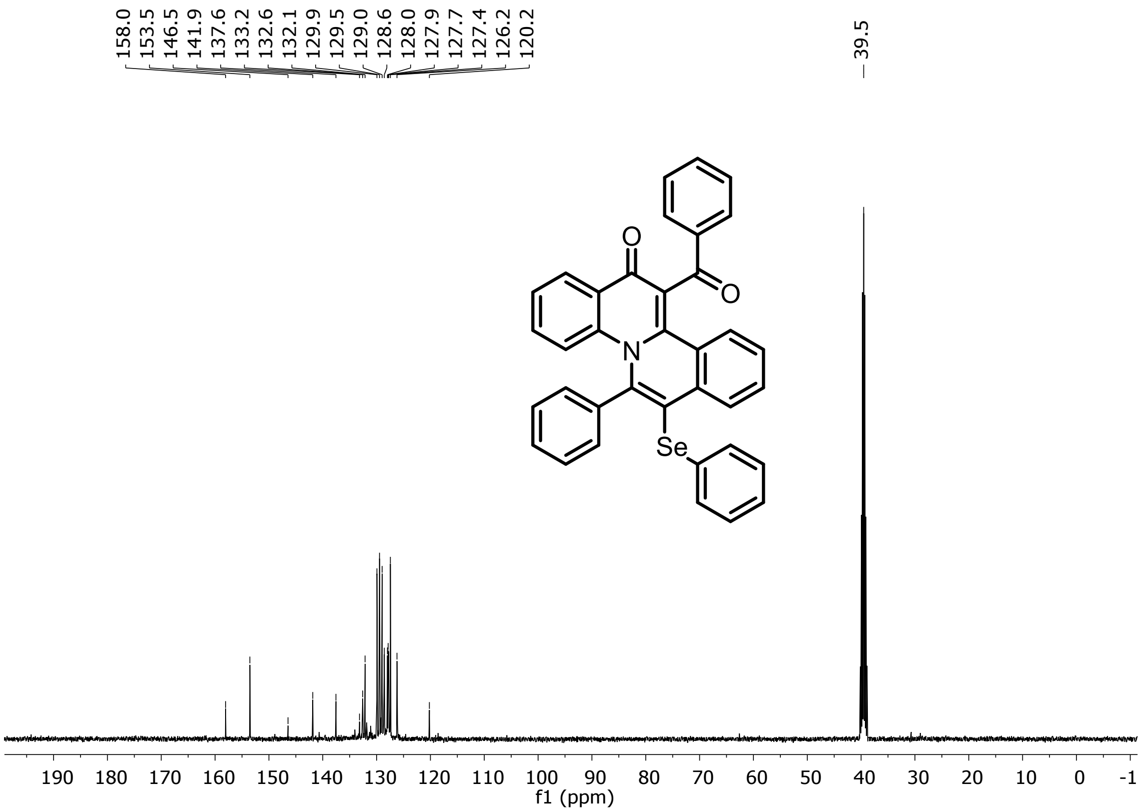

Figure S62. $^{13}C\{^{1}H\}$ NMR (100 MHz, DMSO-d_6) spectrum of the compound **6**.

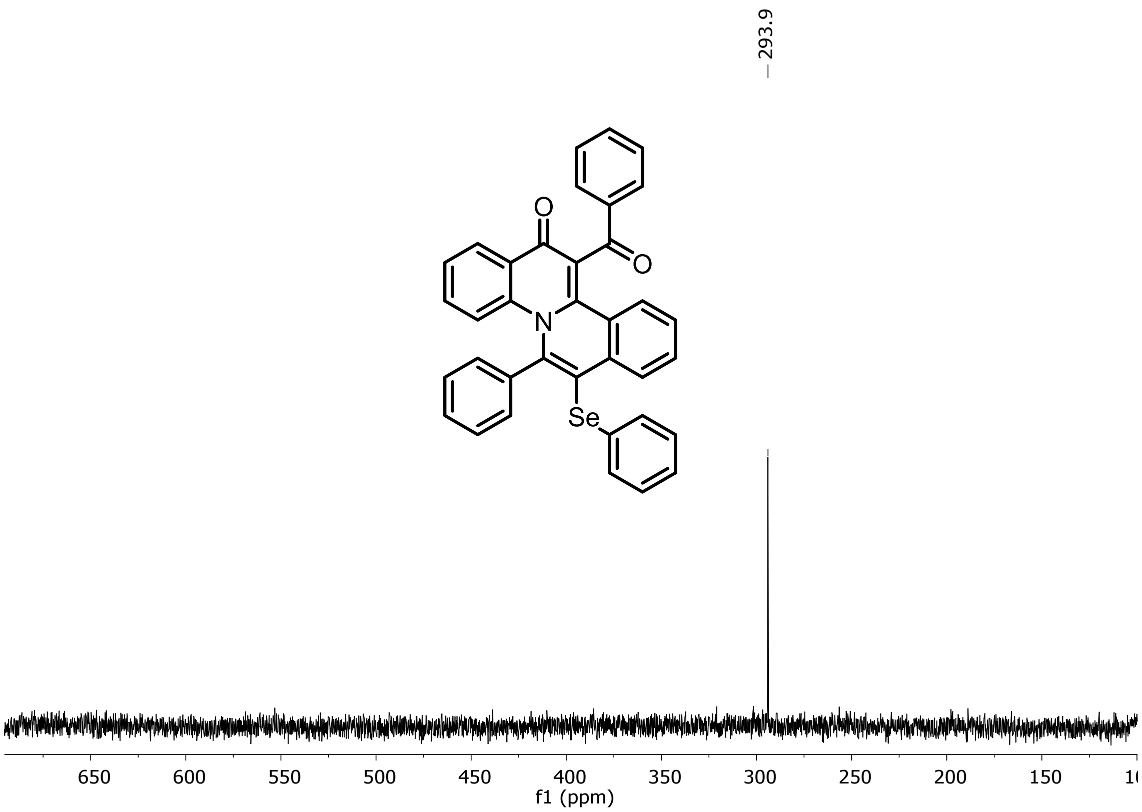

Figure S63. $^{77}Se\{^1H\}$ NMR (76 MHz, DMSO-d_6) spectrum of the compound **6**
